# Development of a cancer metastasis-associated risk model via multi-machine-learning algorithms for prognostic risk evaluation and clinical application in oral squamous cell carcinoma

**DOI:** 10.1186/s12967-025-07336-y

**Published:** 2025-11-24

**Authors:** Xu Han, Tiantian Sun, Yuanyuan Dai, Ruohan Yun, Haiqiang Wang, Junru Jia, Xiangyuan Feng, Mengyun Jiao, Mengwen Hou, Man Yue, Shuo Jiang, Guosen Zhang, Yang An, Dayong Wang

**Affiliations:** 1 Department of Biochemistry and Molecular Biology, School of Basic Medical Sciences, Henan University, Jinming Street, Kaifeng, Henan 475004 China; 2 Department of Nuclear Medicine, The First Affiliated Hospital of Henan University, Henan University, Kaifeng, 475004 China; 3 School of Stomatology, Henan University, Kaifeng, 475004 China; 4 Henan Provincial Engineering Center for Tumor Molecular Medicine, Kaifeng Key Laboratory of Cell Signal Transduction, Henan University, Kaifeng, 475004 China

**Keywords:** Oral squamous cell carcinoma, Machine learning, Prognostic modeling, Mutation landscape, Tumor immune microenvironment, Molecular docking, Epithelial-mesenchymal transition, Anoikis

## Abstract

**Background:**

Oral squamous cell carcinoma (OSCC) represents a highly malignant form of cancer characterized by molecular heterogeneity and unsatisfactory treatment outcomes, with approximately 50% of patients experiencing local recurrence and distant metastasis following therapy. Given that metastasis is the most critical determinant of OSCC prognosis, enhancing the precision of clinical interventions and identifying therapeutic targets are of paramount importance. In view of this, this study is the first to develop a machine-learning-based prognostic model integrating epithelial-mesenchymal transition (EMT), anoikis, and basement membrane remodeling genes.

**Methods:**

We systematically evaluated 78 algorithm and parameter combinations to identify a robust prognostic model, stratifying patients into High- and Low-risk groups. Kaplan-Meier survival curves and receiver operating characteristic (ROC) analyses were employed to evaluate the predictive performance of this model. Functional enrichment of differentially expressed genes (DEGs) between risk groups revealed key OSCC progression mechanisms. We further analyzed tumor mutation burden, immune microenvironment features, and identified candidate drugs through sensitivity prediction and molecular docking.

**Results:**

The identified 13-gene prognostic model effectively stratified patients into high- and low-risk groups, demonstrating strong predictive power for overall survival: the high-risk group exhibited worse prognosis. Mutation landscape demonstrated significant genetic variability within these model genes, which provided insights into the association between elevated tumor mutational burden and adverse prognostic outcomes. Immune landscape revealed a distinct tumor microenvironment: high-risk group exhibited altered immune cell infiltration, along with increased tumor purity, reduced ESTIMATE score and poorer anticipated response to immunotherapy. Finally, seven promising therapeutic candidates were identified through integrated computational drug screening.

**Conclusion:**

We developed and validated a 13-gene prognostic model that integrates metastasis-related processes, improves survival prediction, and identifies therapeutic opportunities in OSCC.

**Supplementary Information:**

The online version contains supplementary material available at 10.1186/s12967-025-07336-y.

## Introduction

Oral squamous cell carcinoma (OSCC) is one of the most common cancers worldwide [1]. According to GLOBOCAN 2022, it causes 389,485 new cases and 188,230 deaths globally [2], with its incidence projected to increase by approximately 40% by 2040 [3]. OSCC is linked to risk factors, including nut chewing, alcohol/tobacco abuse, HPV infection [4], and environmental exposure to dust and heavy metals [3].

The major treatment for OSCC is surgical resection combined with radiotherapy or chemotherapy for the primary tumor [5]. However, surgery impairs the oral function and aesthetics [3, 6], and chemotherapy harms normal rapidly proliferating cells. Moreover, its frequent use at suboptimal doses leads to drug resistance and metastatic disease [5, 6]. Targeted or CAR-T cell therapy has also shown limited success [7]. EGFR-targeted therapy and cetuximab treatment failed to significantly improve outcomes of OSCC patients [6]. In 2016, immune checkpoint inhibitors (anti-PD1/PD-L1) were approved for OSCC treatment. However, response rates remain low (about 20%), and immune-related toxicities limit their application [4, 6]. Even pembrolizumab (combined with chemotherapy or as monotherapy for metastatic/unresectable OSCC) has low response rates. Although much effort has been made to treat OSCC, its 5-year survival rate is only 50–60% [8]. Therefore, it is urgent to explore improved prognostic assessment tools and effective treatment strategies for OSCC.

For OSCC, metastasis is the most critical factor influencing OSCC prognosis [6]. Half of OSCC patients who receive treatment develop local recurrence and distant metastasis [9] to the lymph nodes, lungs, bone or liver [3]. Unlikely, metastasis of OSCC is difficult to treat with traditional surgery [10]. Moreover, numerous studies have indicated that basement membrane (BM), epithelial-mesenchymal transition (EMT) or anoikis resistance is closely associated with tumor metastasis. Yet these processes have rarely been integrated in OSCC research. This gap motivated us to integrate genes related to BM, EMT and anoikis, and apply machine learning to construct a promising prognostic index for OSCC, termed the Tumor Metastasis-related Index (TMI). BM as a natural barrier between the epithelial and the underlying extracellular matrix (ECM) [11], its breakdown enables cancer cells to invade the underlying stroma, marking the start of metastasis [12]. Epithelial-mesenchymal transition (EMT) is a transdifferentiation process. It endows tumor cells with invasion ability, stress resistance, and dissemination potential [13–15], and may play a critical role in the dissemination of cells from the primary tumor to distant metastatic foci [16]. Additionally, EMT also does contribute to chemoresistance [14, 17]. Anoikis is a form of programmed cell death, which is essential for the survival of tumor cells after detachment from the ECM [18, 19]. The generation of anoikis resistance in aggressive tumor cells has been identified as a key factor in tumor progression [16, 20]. It has been proposed that EMT also plays a key role in making cancer cells anoikis resistant [19, 20].

Here, we integrated basement membrane, EMT, and anoikis-related genes to construct a novel prognostic index (TMI) for OSCC using machine learning. As a prognostic risk assessment model, TMI showed significant prognostic ability and demonstrated strong performance in predicting response to immunotherapy and pharmacotherapy. Our findings may provide an important reference point for personalized treatment for OSCC patients.

## Methods

**Fig. 1:**
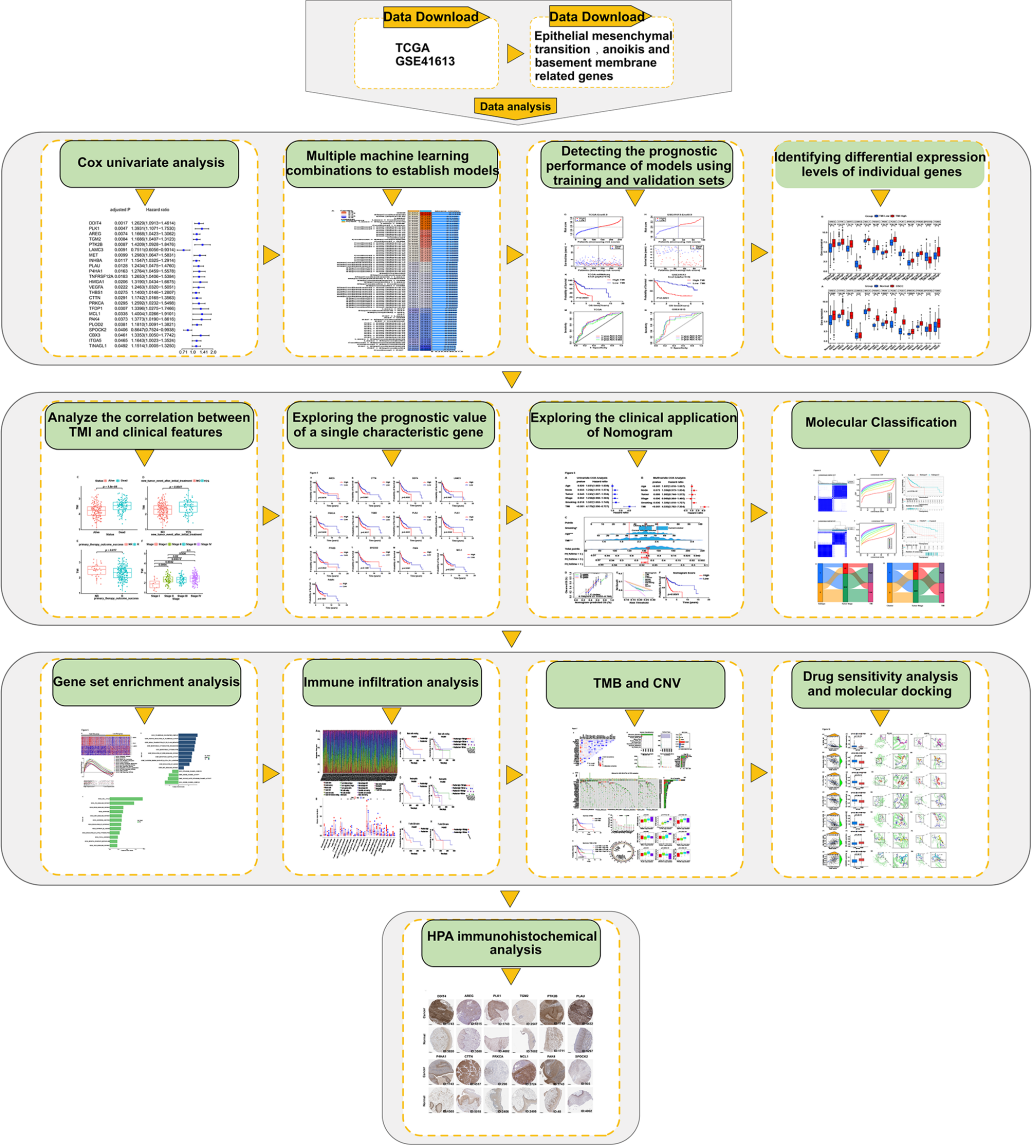
The flow chart of this research

The flow chart of this research was shown in Fig. 1.

### Data collection

Gene expression profiling and survival information of OSCC was obtained from The Cancer Genome Atlas (TCGA, https://portal.gdc.cancer.gov/). The included samples have to meet the following criteria: (**A**) site of the primary tumor belongs to OSCC areas [6, 21] (i.e., tongue, base of tongue, border of tongue, lip, etc.); (**B**) containing data on survival status and survival time; (**C**) frozen sections only (paraffin-embedded samples excluded) Additionally, microarray data from GSE41613 were sourced from the Gene Expression Omnibus (GEO) database. After screening, 262 OSCC and 18 normal samples from TCGA, and 97 OSCC samples from GSE41613 were included (Table 1). HPV or smoking state of OSCC from TCGA was available using PanCancer seq data [22]. Missing rates were 1.5% (4/262) for HPV status and 1.9% (5/262) for smoking history. Both variables were considered for inclusion in the nomogram. Clinical information, CNV and masked somatic mutation data were acquired from UCSC Xena (http://xena.ucsc.edu) and TCGA database, respectively.

**Table 1:** Datasets included in this study

Cohort	TCGA (n)	GSE41613 (n)
**Sample size**		
Cancer	262	97
normal	18	0
**Age**		
< 60	123	50
≥60	139	47
**Gender**		
Male	183	31
Female	79	66
**Stage**		
Stage I-II	71	41
Stage III-IV	181	56
**Drinking**		
Yes	179	NA
No	78	NA
**Smoking**		
Current smoker	89	NA
Current reformed smoker	89	NA
Lifelong non-smoker	76	NA
**HPV**		
Negative	232	97
Positive	26	0

### Summary of basement membrane, anoikis or EMT-related genes

Based on previous researches [23], the basement membrane-related genes were collected. Anoikis-related genes were derived from GSEA (YAN_ESCAPE_FROM_ANOIKIS), GeneCards (relevance score > 0.4), and Harmonizome (GeneRIF Biological Term Annotations, GO Biological Process Annotations and GO Biological Process Annotations 2023). Genes overlapping in at least two sources were retained. The EMT-related genes were compiled from previously reported studies [13, 17, 24–26]. Genes were counted per process independently, allowing multi-functional genes to contribute to each relevant category. This counting strategy yielded a set of 499 genes. Above gene sets were shown in Table S1.

### Establishment of a prognostic signature by integrative machine learning approaches

The TCGA-OSCC cohort (*n* = 262) was used for model training, and the GSE41613 served as an independent external validation cohort. Genes with expression levels below 0.1 were excluded to mitigate the influence of extreme values To ensure the high predictive accuracy, models were constructed according to the following pipeline: 1) Univariate Cox analysis in the TCGA cohort with “survival” package identified 24 genes which were considered to have potential prognostic ability for subsequent variable selection. 2) Following established machine learning frameworks for prognostic modeling [27–29] to select algorithms and parameters, we exclusively used algorithms with intrinsic feature selection capacity, including Lasso, stepwise Cox, CoxBoost, RSF, Enet and GBM, to automatically shrink or eliminate redundant variables, thereby mitigating overfitting. Ridge regression served as a non-feature-selecting comparator. For Enet, the tradeoff parameter α was tuned from 0 to 1 at 0.1 intervals as recommended. In total, 78 combinations of these seven algorithms and parameters were applied to construct models using TCGA cohort. 3) The C-index ranked all models, and the most prognostically accurate and clinically relevant one was selected, and its risk score was termed the TMI [29]. 4) The parameter tuning details were described as follows: models were trained under a uniform 10-fold cross-validation framework to select optimal settings. This process determined: the number of boosting steps in CoxBoost; the optimal λ for Lasso, Enet and Ridge (via “cv.glmnet”); the optimal number of boosting iterations (n.trees) in GBM. Hyperparameter settings and penalization mechanisms were used to control model complexity. Specifically, CoxBoost employed a built-in regularization penalty; RSF applied the “var.select” function to exclude non-informative features; stepwise Cox regression used AIC to penalize overly complex models; Lasso introduced L1 regularization for variable shrinkage and selection; Ridge utilized L2 regularization to shrink coefficients without feature elimination; and Enet combined both L1 and L2 regularization through a trade-off parameter α (ranging from 0 to 1). Together, these strategies balanced model fit with parsimony and substantially mitigated overfitting.

### Survival analysis and potential clinical application demonstrated by nomogram

According to the median value of TMI, OSCC patients were stratified into High-TMI and Low-TMI groups. Kaplan Meier survival curves and ROC analyses were performed using the “survival” and “survivalROC” R packages. To enhance the clinical utility and prognostic accuracy of TMI, it was integrated with clinical data for subsequent analysis. Clinically significant features from univariate Cox analysis and TMI were included in a multivariate Cox proportional hazards model (using the coxph function with standard procedures). Independent prognostic factors identified were incorporated into a nomogram. Validation included calibration curves, C-index, Kaplan Meier survival curves, and decision curve analysis (DCA).

### Identification of differentially expressed genes and enrichment analysis

Using the “limma” package, DEGs were identified from 262 OSCC and 18 normal samples with thresholds of *p* < 0.05 and |log2FC| > 1. Significant DEGs underwent GO enrichment analysis via “clusterProfiler”, with pathways considered significant at *p* < 0.05. GSEA or GSVA was performed on the complete expression matrix. GSEA analysis employed javaGSEA v4.3.3 with the “c2.cp.kegg.v2023.1.Hs.symbols.gmt” gene set, and pathways with |NES| > 1, *p* < 0.05 and FDR < 0.05 were considered significant. GSVA results were deemed significant at *p* < 0.05 and FDR < 0.05.

### Landscape analysis of OSCC mutations and immune microenvironment

In mutation analysis, somatic mutations were analyzed with maftools, CNV-expression correlations were performed by Kruskal-Wallis test, and mutation patterns were examined through Fisher’s exact test.

Immune infiltration was assessed using two methods: CIBERSORT quantified immune cell abundances while ssGSEA evaluated immune activity levels. We further compared immune checkpoint expression and ESTIMATE scores between High-TMI and Low-TMI groups. Immunotherapy response was predicted using TIDE and validated with the GSE35640 dataset.

### Drug sensitivity analysis and molecular docking

Drug sensitivity was predicted with the “oncoPredict” R package. Associations between TMI and drug response were evaluated using T-tests for group comparisons and Pearson correlation for linear relationships. Drugs were selected based on consistent significant correlation (*p* < 0.05) with TMI across IC50 data from GDSC1, GDSC2 and CTRP2 databases. Inclusion required either a negative correlation (*R* < −0.4) or a positive correlation (*R* > 0.35) in at least one database. Molecular docking utilized drug structures from PubChem and protein targets from PDB. Docking simulations employed the Genetic Algorithm in AutoDock with 10 runs under default parameters. Binding energies were computed and interaction patterns visualized using PyMol.

### Statistical analysis and HPA validation

Statistical analyses were performed using R (v4.4.1/4.3.1/4.3.0). When plotting Kaplan Meier survival curves, patients were consistently stratified based on the median cutoff of a certain indicator. Group comparisons used t-test or Wilcoxon test based on data distribution, and correlations were evaluated by Pearson method. Unless otherwise specified, *p* < 0.05 was considered statistically significant. Protein expression was validated using HPA data (Table S4).

## Results

### Establishment and validation of an optimal prognostic signature based on integrative machine learning algorithms

**Fig. 2:**
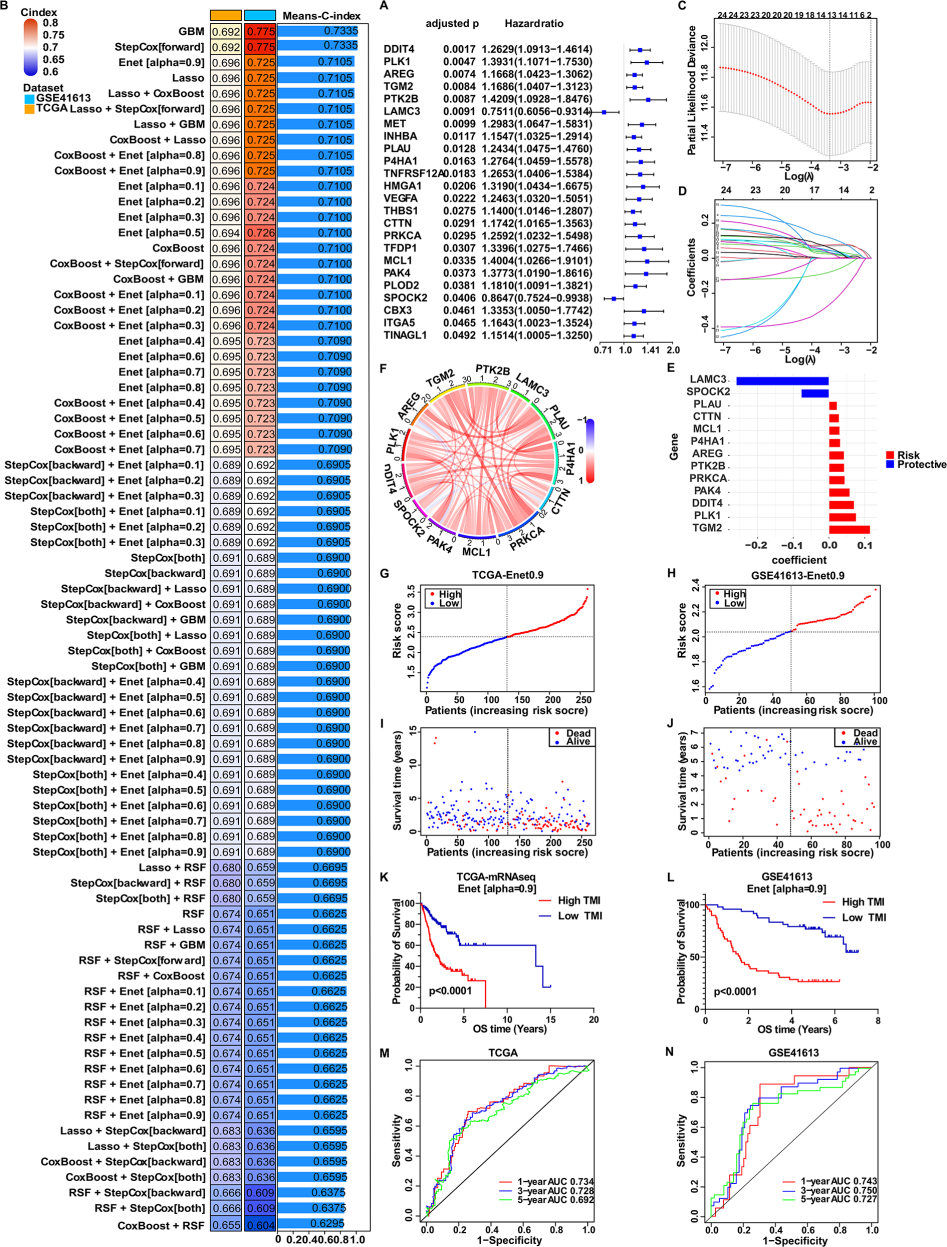
Development and validation of prognostic model based on multiple combination machine learning algorithms. (**A**) Identification of survival-related genes using univariate Cox regression. (**B**) C-index computed across TCGA and GSE41613 cohorts, with models ranked by mean values, representing the ability of models to correctly rank patients by survival risk. (**C**) Parameter tuning of Enet[α = 0.9] model based on tenfold cross validation framework to determine the number of signature genes. The two dashed lines before and after in the figure respectively showed Lambda.Min and lambda.1se, where lambda.Min corresponds to the final 13 signature genes. (**D**) Distribution of Enet[α = 0.9] coefficients for survival-related genes obtained from univariate Cox regression. (**E**) The coefficients of 13 signature genes modeled by Enet[α = 0.9]. (**F**) Correlation analysis of 13 signature genes. (**G-H**) Distribution of TMI in TCGA and GSE41613 datasets, respectively. (**I-J**) Survival status of High-TMI and Low-TMI patients in TCGA and GSE41613 datasets, respectively. (K-L) Kaplan Meier survival curves using median cutoff and log-rank test of High-TMI and Low-TMI patients in TCGA and GSE41613 datasets respectively, which can visualize survival differences between High-TMI and Low-TMI groups. (**M-N**) Time dependent ROC analysis of TMI predicting 1-year, 3-year and 5-year overall survival rates in TCGA and GSE41613 cohorts, respectively. AUC measured discrimination accuracy at each time point. C-index, concordance index; TMI, tumor metastasis index; AUC, area under the ROC curve. C-index accuracy tiers: 0.50 < C-index ≤ 0.70 indicates low accuracy; 0.7 < C-index ≤ 0.90 indicates moderate accuracy; C-index >0.90 indicates high accuracy. AUC accuracy tiers: 0.50 < AUC ≤ 0.70 indicates low accuracy; 0.70 < AUC ≤ 0.90 indicates moderate accuracy; AUC >0.90 indicates high accuracy

As research flowchart shown (Fig. 1), analysis began with the identification of tumor metastasis-related gene set (basement membrane, anoikis and EMT-related genes). Using the ssGSEA algorithm to quantify the expression levels of the tumor metastasis-related gene set, the results showed that the expression of these genes was significantly elevated in OSCC samples and also contribute to the diagnosis of OSCC (Fig. S1). These findings suggest a potential correlation between tumor metastasis-related genes and OSCC. A univariate Cox analysis was performed on the TCGA cohort through screening metastasis-related genes to identify 24 prognostic genes (Fig. 2A). Subsequently, based on these 24 prognostic genes, all the model configurations were fitted through machine learning algorithms, and the C-index was calculated for each model in both the TCGA and GSE41613 cohorts to assist in model selection (Fig. 2B). The Enet [alpha = 0.9] model was ultimately selected for its optimal performance. The detailed model selection process is illustrated in Fig. S1. Based on cross-validation, parameters of the Enet [alpha = 0.9] were adjusted by minimizing the partial likelihood deviance. The optimal λ value (0.0344) and gene coefficients in Enet [alpha = 0.9] were determined (Fig. 2C and D), which included 2 protective genes and 11 risk genes (Fig. 2E). The correlation analysis of these 13 genes was shown in Fig. 2F. Thus, these 13 metastasis-related genes contributed to a prognostic model, named the Tumor Metastasis-related Index (TMI). The TMI of patients can be directly calculated using the following formula:

1 $$\eqalign{ TMI & = - 0.2598*LAMC{3^{exp}} - 0.0775*SPOCK{2^{exp}} \cr & + 0.0227*PLA{U^{exp}} + 0.0284*CTT{N^{exp}} \cr & + 0.0301*MCL{1^{exp}} + 0.0318*P4HA{1^{exp}} \cr & + 0.0425*ARE{G^{exp}} + 0.0430*PTK2{B^{exp}} \cr & + 0.0445*PRKC{A^{exp}} + 0.0585*PAK{4^{exp}} \cr & + 0.0707*DDIT{4^{exp}} + 0.0764*PLK{1^{exp}} \cr & + 0.1157*TGM{2^{exp}} \cr} $$

Based on the median TMI, patients in the TCGA or GSE41613 cohort were divided into High-TMI and Low-TMI groups (Fig. 2G and H). Notably, as TMI increases, the survival time of patients gradually shortens, and the number of deaths increases (Fig. 2I and J). Kaplan Meier survival curves revealed that High-TMI patients had significantly shorter overall survival (OS) than Low-TMI patients (Fig. 2K and L). The ROC analyses showed that the AUC for 1-year, 3-year and 5-year survival in TCGA cohort were 0.734, 0.728 and 0.692, while in GSE41613 cohort, they were 0.743, 0.750 and 0.727, respectively (Fig. 2M and N), demonstrating the strong discriminatory power of TMI. These results establish a 13-gene TMI model with robust survival prediction performance.

### Differential expression analysis of signature genes and exploration of their prognostic abilities

**Fig. 3:**
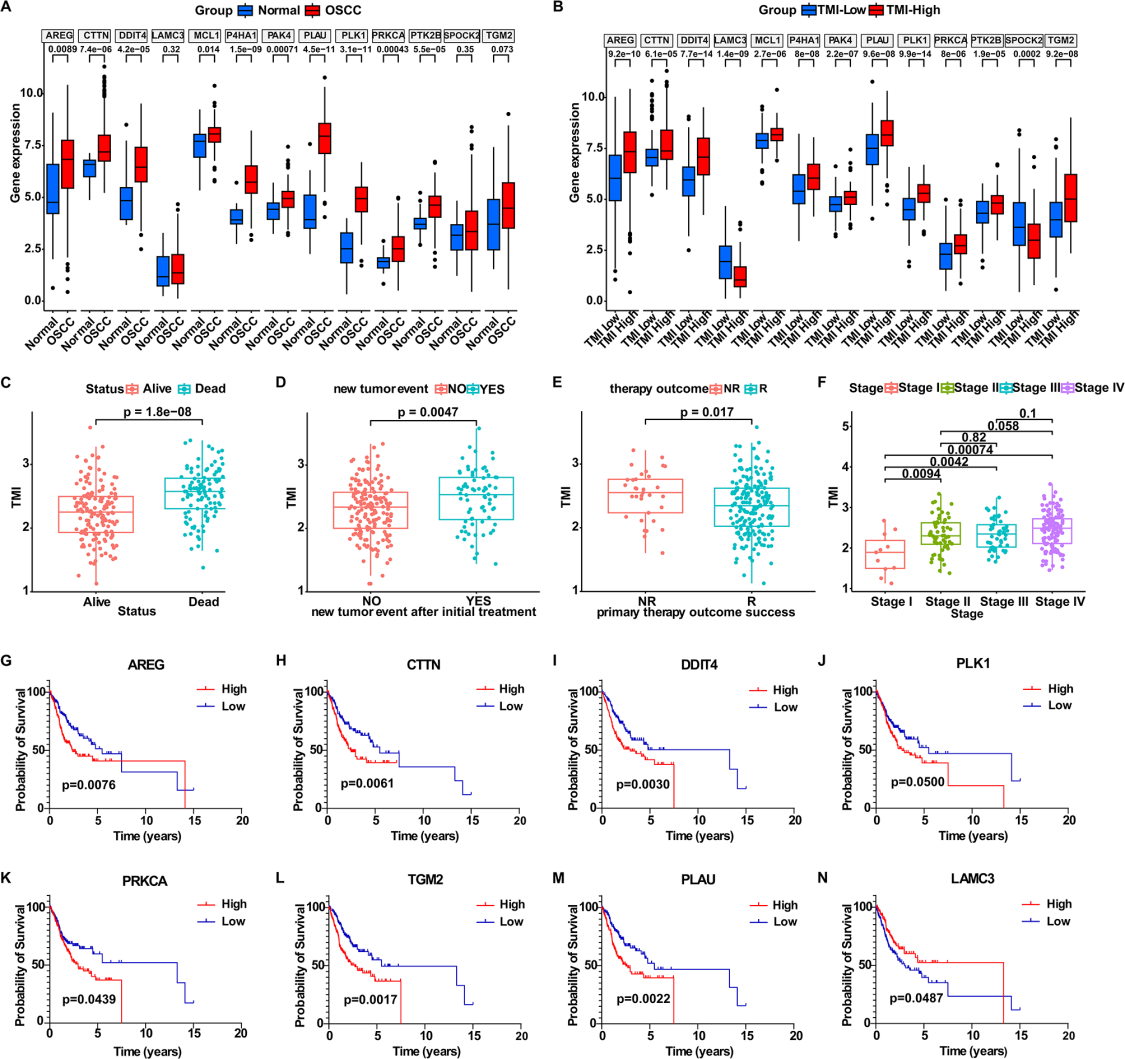
Differential expression and prognostic analysis of signature genes, along with clinical characteristics stratified by TMI. (**A**) Differential expression analysis of signature genes between normal and OSCC tissues, highlighting their tumor-specific expression. (**B**) Differential expression analysis of signature genes between high-TMI and Low-TMI groups, reflecting risk stratification by the TMI model. (**C-F**) Boxplot showing the TMI level between patients with different survival status, postoperative recurrence, treatment outcomes and clinical stages, respectively, demonstrating associations between TMI and clinical outcomes. (**G-N**) Kaplan Meier survival curves of OSCC patients stratified by the expression of signature gene. TMI, tumor metastasis index. Wilcoxon test (**A-F**) and log-rank test with median cutoff (**G-N**) were used, and *p* values were shown

The differential expression analysis of signature genes (Table S2) showed that 10 risk genes were highly expressed in OSCC but down-regulated in normal tissues, suggesting that these genes might play an important role in the occurrence and development of OSCC (Fig. 3A).

Significant results show that risk genes were up-regulated in High-TMI group but down-regulated in Low-TMI group, with opposite patterns for protective genes (Fig. 3B). Visualization of the TMI levels in OSCC patients with different clinicopathological features showed that patients who died, had postoperative recurrence, were non-responsive to treatment or high stage exhibited higher TMI (Fig. 3C–F). This may be consistent with the role of signature genes as biomarkers of cancer progression and prognosis, indicating that TMI might play an important role in clinical management and prognosis evaluation of OSCC patients. Kaplan Meier survival curves for signature genes were individually plotted. As is illustrated, high expression of risk genes was associated with worse overall survival of OSCC patients (Fig. 3G–M), while high expression of protective genes was associated with better OS (Fig. 3N). These findings suggest that these signature genes play an indicative role in the prognosis of OSCC. The Chi-square test of clinicopathological features of 241 primary OSCC patients was shown in Table 2, which revealed significant differences between the High-TMI and Low-TMI groups in terms of OS status, clinical T, clinical N, clinical stage, neoplasm cancer status, perineural invasion, lymphovascular invasion, and followup treatment outcome. As is shown, patients with High TMI Risk exhibited higher incidence of death, more severe clinical pathological staging, with tumor status, more lymphovascular invasion or poorer response to treatment (Table 2), and these results may partly explain the prognosis differences between the High-TMI and Low-TMI groups. The expression patterns and prognostic impacts of signature genes support their values as clinical biomarkers.

**Table 2:** Characteristic chi-square test results of 241 clinical cases of primary OSCC

Characteristic	patients (n)	n%	High TMI Risk	Low TMI Risk	Chi-square	p value
**total number**	241	100.00%	119	122		
**OS status**					63.72	< 0.0001****
Dead	84	34.85%	71	13		
Alive	157	65.15%	48	109		
**Clinical_T**					13.55	0.0036**
T1	19	7.88%	6	13		
T2	73	30.29%	27	46		
T3	69	28.63%	35	34		
T4	80	33.20%	51	29		
**Clinical_N**					14.62	0.0056**
N0	123	51.04%	53	70		
N1	45	18.67%	24	21		
N2	58	24.07%	41	17		
N3	2	0.83%	0	2		
NX	3	1.24%	1	2		
**Clinical stage**					10.9	0.0123*
Stage I	11	4.56%	2	9		
Stage II	55	22.82%	22	33		
Stage III	52	21.58%	23	29		
Stage IV	123	51.04%	72	51		
**Neoplasm cancer status**					18.29	< 0.0001****
Tumor free	145	61.96%	56	89		
With tumor	89	38.04%	60	29		
**Perineural invasion**					11.16	0.0038**
Yes	87	36.10%	40	47		
No	96	39.83%	59	37		
**Lymphovascular invasion**					9.617	0.0082**
Yes	55	22.82%	35	20		
No	121	50.21%	61	60		
**Followup treatment outcome**					17.12	0.0089**
Complete remission/Response	109	45.23%	41	68		
Partial remission/Response	1	0.41%	1	0		
Persistent disease	5	2.07%	5	0		
Progressive disease	43	17.84%	27	16		
Stable disease	3	1.24%	2	1		

### Construction and evaluation of a nomogram for survival prediction

**Fig. 4:**
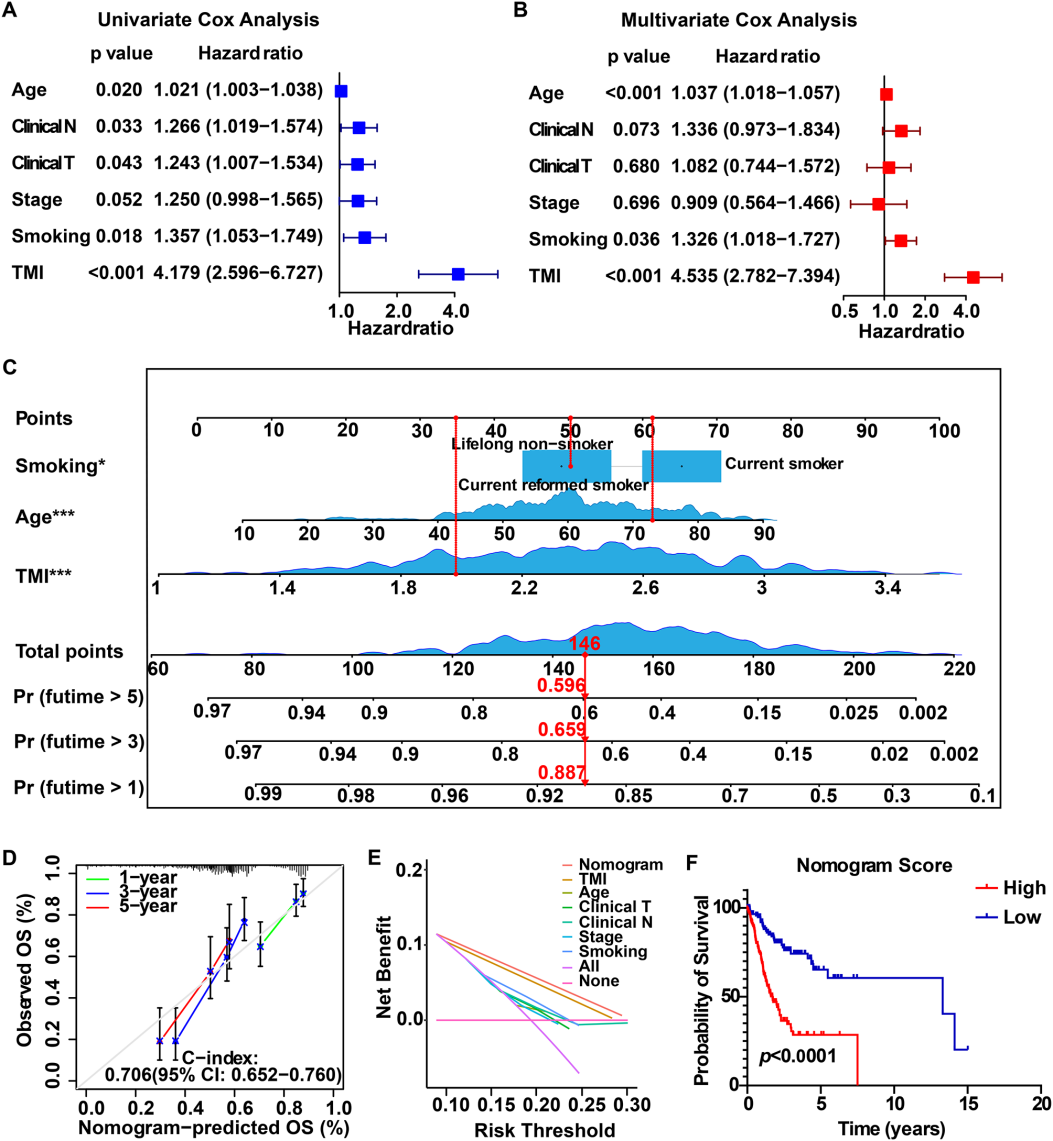
Establishment and evaluation of the nomogram for survival prediction. (**A**) Univariate Cox regression analysis of clinical characteristics and TMI. (**B**) Multivariate Cox regression analysis of clinical characteristics and TMI in the TCGA cohort. (**C**) Nomogram for predicting 1-year, 3-year and 5-year overall survival rates. (**D**) Calibration plots showing the reliability of nomogram in predicting 1-year, 3-year and 5-year overall survival rates. (**E**) DCA assessing the clinical utility of clinic characteristics, TMI and nomogram. The “none” line (no patient treated, net benefit = 0) and the “all” line (all patients treated, net benefit declines with threshold due to overtreatment) serve as reference curves. A model demonstrates clinical application when its curve exceeds both benchmarks. (**F**) Kaplan Meier survival curves of OSCC patients based on nomogram score (median cutoff; log-rank test). TMI, tumor metastasis index; DCA, decision curve analysis

To enhance the applicability and convenience in clinical practice of this model, TMI along with clinicopathological features (including smoking, drinking alcohol, HPV, clinical_N, clinical_M, clinical_T, stage and neoplasm histologic grade) were incorporated as candidates for subsequent analysis. The combination of univariate and multivariate Cox regression was employed to identify robust and independent prognostic factors, rather than for numerical performance assessment, which was instead quantified using the C-index. The statistically significant results of the univariate Cox regression were shown in Fig. 4A. With only five variables analyzed in > 200 samples (Fig. 4B), the EPV ratio exceeded 10, ensuring model stability and minimal overfitting risk. Ultimately, variables including age (*p* < 0.001), smoking (*p* < 0.05), and TMI (*p* < 0.001) were independently predictive and applied to develop a nomogram (Fig. 4A–C). The calibration curve demonstrated the accuracy of this nomogram in predicting 1-year, 3-year and 5-year survival rates, yielding a C-index of 0.706 (Fig. 4D). Furthermore, decision curve analysis (DCA) revealed that this nomogram outperformed all other predictive factors considered here, demonstrating its superior net clinical benefit and potential to guide individualized treatment decisions. Figure 4E based on the nomogram score, significant survival differences were observed between the high-score and low-score groups (Fig. 4F). The TMI-based nomogram offers accurate survival prediction and practical risk stratification.

### Exploration of potential molecular mechanisms for OSCC progression through multiple enrichment analyses and unsupervised clustering

**Fig. 5:**
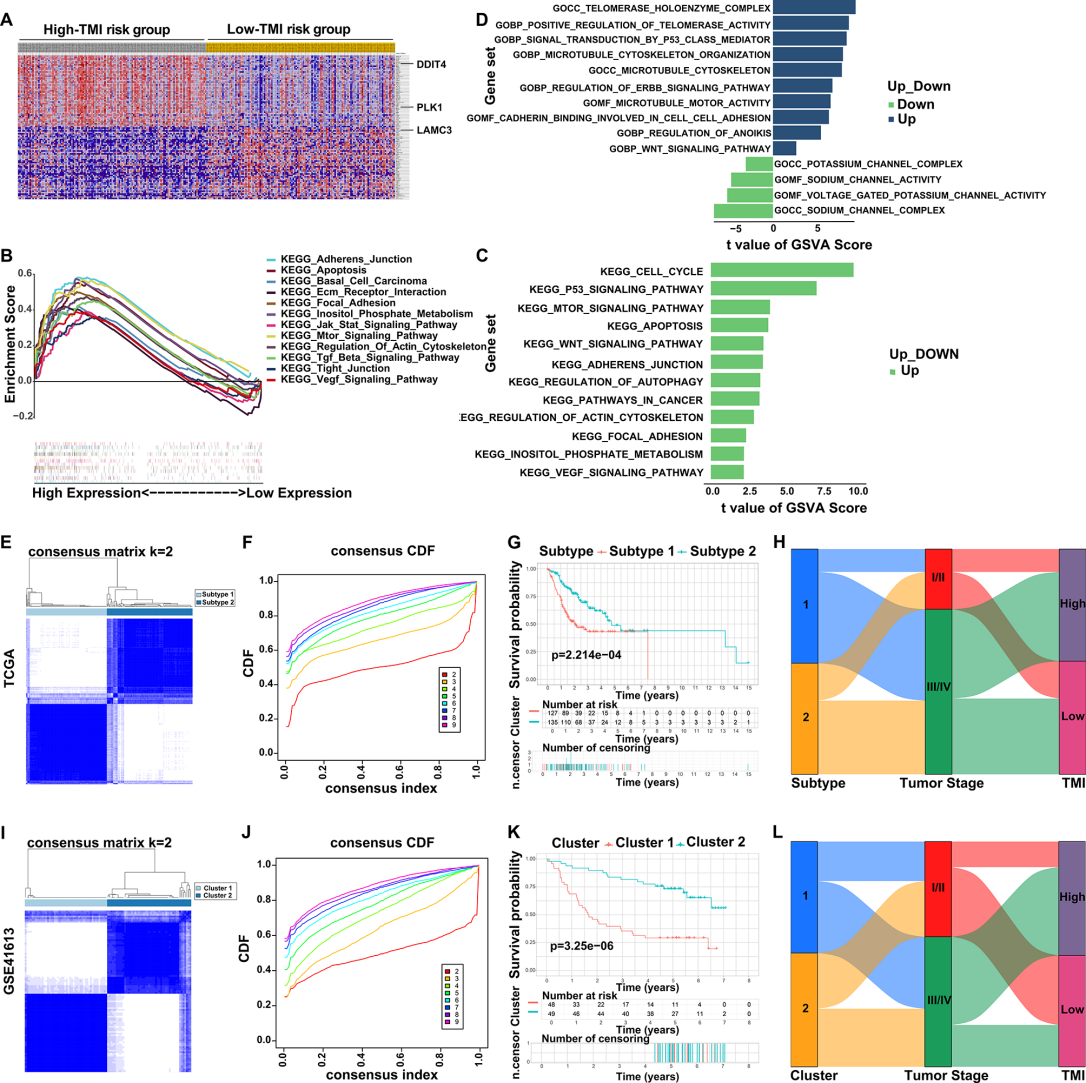
Enrichment and unsupervised clustering analysis based on TMI model genes. (**A**) GSEA heatmap illustrated by different TMI groups. (**B**) Enrichment score for each enriched pathway. (**C-D**) KEGG and GO analysis of high-TMI and Low-TMI groups based on GSVA. (**E-L**) TMI-specified molecular subtyping of OSCC. (**E**) Unsupervised clustering analysis of TCGA cohort at k = 2 revealed two molecular subtypes. (**F**) Empirical cumulative distribution function plot displayed consensus distributions across k-values (ranging from 2 to 10). (**G**) Kaplan Meier survival curves (log-rank test and median cutoff) of two molecular subtypes. (**H**) Alluvial diagram visualizing the relationships among molecular subtypes, tumor stage and TMI groups in OSCC patients. (**I-L**) Unsupervised clustering analysis of GSE41613 cohort to validate the results of TCGA cohort. GSEA, gene set enrichment analysis; GSVA, gene set variation analysis; KEGG, kyoto encyclopedia of genes and genomes; GO, gene ontology; TMI, tumor metastasis index

The mRNAsi-based stemness index reflects the gene expression characteristics of cancer cells and is associated with tumor stem cell activity, dedifferentiation and invasiveness [30]. The analysis showed that the High-TMI group has a higher stemness index, which to some extent validated the reliability of the tumor metastasis-related gene set (Fig. S3). Moreover, in dataset GSE275870, lymph node metastases exhibited higher TMI scores than primary tumors, further confirming the predictive value of TMI to OSCC metastatic potential (Fig. S4). To further explore the potential molecular mechanisms underlying the impact of TMI on OSCC progression, GSEA and GSVA enrichment analyses were respectively performed for both the High-TMI and Low-TMI groups (Table S3). Through GSEA, pathways enriched in the High-TMI risk group were strongly associated with tumor development and metastasis, including VEGF, TGF-β and JAK-STAT pathway (Fig. 5A and B). The results of GSVA-based KEGG enrichment were generally consistent with that of GSEA, with some pathways showing enrichment in both analyses (Fig. 5B–D). This concordance highlights the reliability of both approaches in assessing biological pathway activity differences between High-TMI and Low-TMI group, thereby enhancing the robustness of these findings. Additionally, GO analysis using GSVA showed that ion channel-related pathways were more enriched in the Low-TMI group compared to the High-TMI group (Fig. 5D). Based on DEGs between the High-TMI and Low-TMI groups, GO enrichment analysis revealed that the identified pathways were predominantly associated with ion channels and the cytoskeleton, further reinforcing these above findings (Fig. S5). In short, these identified pathways enriched in High-TMI or Low-TMI group may contribute to the progression of OSCC and thus act as therapeutic targets for patients with different prognostic risk levels, offering potential perspectives for clinical treatment strategies in a TMI risk-specific manner.

To further explore the molecular mechanisms of TMI-mediated OSCC progression and molecular heterogeneity, molecular subtyping based on TMI model genes was performed and validated. In the TCGA cohort, unsupervised clustering analysis (ConsensusClusterPlus) identified two groups (Fig. 5E). k = 2 was selected because the consensus CDF curve became relatively flat and the delta area showed minimal gain beyond k = 2 (Fig. 5F). Therefore, we defined these two groups as Subtype 1 and Subtype 2, which displayed significantly different overall survival (*p* < 0.001, Fig. 5G). Subtype 1 patients had worse prognosis, higher proportion in Stage III/IV and higher TMI risk level (Fig. 5H). For validation, the same approach was applied to the GSE41613 cohort, which also identified two groups (Fig. 5I–J). To distinguish this finding from that of the TCGA cohort, these groups were named Cluster 1 and Cluster 2. Cluster 1 showed worse prognosis, higher Stage III/IV proportion and higher TMI risk level (Fig. 5K–L). Enrichment and clustering analyses revealed TMI-linked pathways and prognostic subtypes.

### Mutation landscape characterization of OSCC patients

**Fig. 6:**
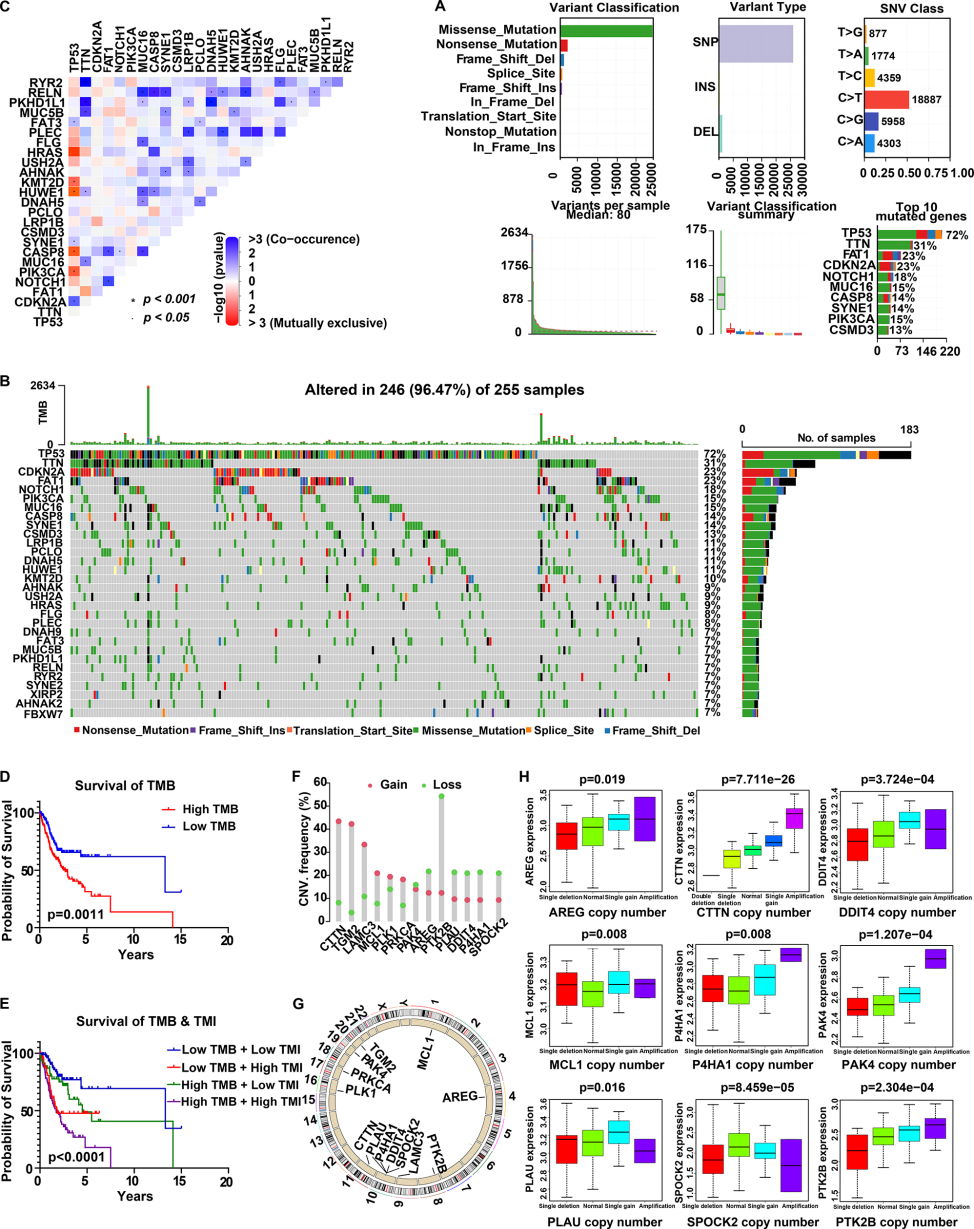
TMI-related mutation landscape of OSCC patients. (**A**) Distribution and frequency analysis of mutation types across genes. (**B**) Waterfall plot illustrating gene mutation patterns in OSCC patients. (**C**) Heatmap displaying the co-occurrence and mutual exclusivity of gene mutations. (**D**) Kaplan-meier survival curves of OSCC patients based on TMB. (**E**) Kaplan-meier survival curves of OSCC patients stratified by TMB and TMI level. (**F**) Frequency of CNVs in signature genes. (**G**) Chromosomal localization of signature genes. (**H**) Correlation between signature gene expression levels and CNVs. TMI, tumor metastasis index; TMB, tumor mutational burden; CNVs, copy number variations. Fisher’s exact test (**C**), log-rank test (**D-E**), and kruskal-wallis test (**H**) were used; *p* values were shown

To further explore potential molecular mechanisms for the pathogenesis of OSCC, mutation characteristic of OSCC was estimated (Fig. 6A). In TCGA cohort, approximately 96.47% (246/255) of OSCC patients exhibited mutations, with the majority being missense mutations (Fig. 6B). Notably, the top four genes in terms of mutation frequency, including *TP53* (1st), *CDKN2A* (3rd), *PIK3CA* (6th) and *CASP8* (8th), are all implicated in the anoikis-related genes. Additionally, *USH2A*, ranked 17th in mutation frequency, is a gene associated with the basement membrane (Fig. 6B). A heatmap of the co-occurrence and mutual exclusivity of gene mutation revealed a mutually exclusive relationship between *TP53* and *CASP8*, while *TP53* and *CDKN2A* showed a co-occurrence relationship (Fig. 6C). These findings suggest that disruptions in the anoikis mechanism and alterations in basement membrane integrity, driven by specific genetic mutations, may play a pivotal role in OSCC pathogenesis/progression. Moreover, high tumor mutation burden (TMB) predicted worse survival, and combined analysis of TMB with TMI further stratified prognosis (Fig. 6D–E). In contrast, CNV profiling of TMI signature genes highlighted recurrent genomic alterations: the EMT-related gene *CTTN* on chromosome 11 exhibited amplification in nearly 50% of samples, whereas the anoikis-related gene *PTK2B* on chromosome 8 displayed deletion in over 50% of samples (Fig. 6F–G). Notably, expression of nine signature genes was significantly correlated with their respective CNVs (*p* < 0.05, Fig. 6H). Taken together, these results indicate that mutations in *TP53*, *CDKN2A*, *PIK3CA* and *CASP8*, along with CNV-driven alterations in *CTTN* and *PTK2B*, represent key driver events that may contribute to OSCC progression.

### Tumor immune microenvironment profiling of OSCC patients

**Fig. 7:**
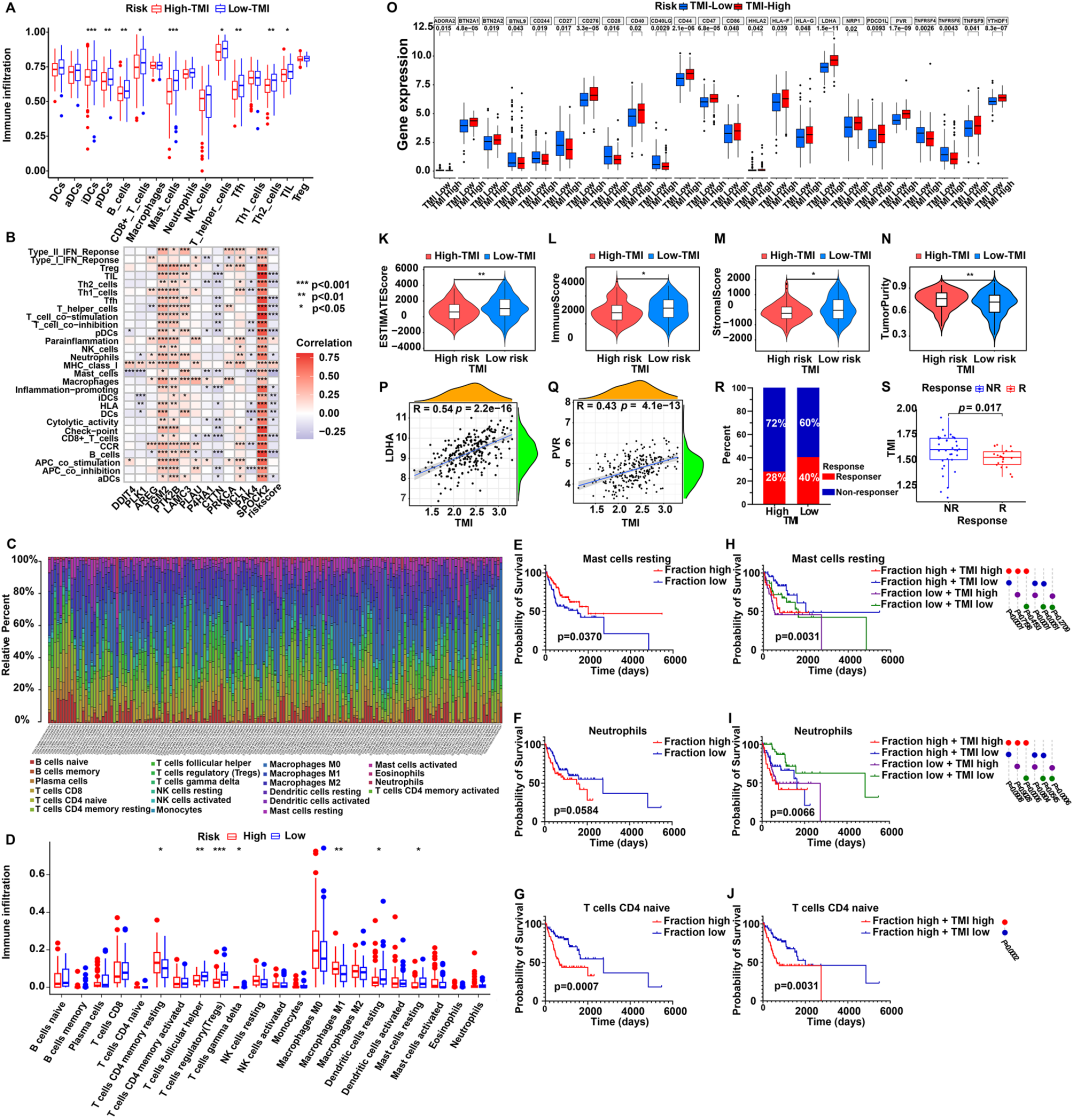
TMI-related tumor immune microenvironment profiling of OSCC patients. (**A**) Boxplot showing differences in infiltration levels of 16 immune cell types between high-TMI and Low-TMI groups based on ssGSEA analysis. (**B**) Correlation analysis of signature genes, TMI, 16 immune cell types and 13 immune functions. (**C**) Proportions of immune cells infiltration for each patient. (**D**) Boxplot showing infiltration levels of 22 immune cell types in high-TMI and Low-TMI groups based on CIBERSORT analysis. (**E-G**) Kaplan-meier survival curves elucidate the prognostic values of the proportion of mast cells resting, neutrophils and T cells CD4 naive in the OSCC microenvironment, respectively. (**H-J**) Survival analysis of OSCC patients based on comprehensive immune cell abundance and TMI level. (**K-N**) Comparisons of estimate score, immune score, stromal score and tumor purity between high-TMI and Low-TMI groups. (**O**) Differential expression analysis of immune checkpoints between high-TMI and Low-TMI groups. (**P-Q**) Correlation analysis of TMI with immune checkpoints, LDHA and PVR, respectively. (**R**) TIDE analysis showing the percentage of immunotherapy responders and non-responders in high-TMI and Low-TMI groups within the GSE35640 cohort. (**S**) Comparison of TMI level between immunotherapy response groups in the GSE35640 cohort. ssGSEA, single sample gene set enrichment analysis; estimate, estimation of stromal and immune cells in malignant tumor tissues using expression data; TMI, tumor metastasis index. Wilcoxon rank-sum test (**A, D, K-O**), Pearson correlation (**B, P-Q**), and log-rank test (**E-J**) were used; *p* values are shown

To explore the tumor immune microenvironment (TME) of OSCC patients and the potential application of TMI in guiding immunotherapy, immune infiltration analyses were performed. Using ssGSEA, the Low-TMI group showed enriched activation of dendritic cells (iDCs and pDCs), B cells, mast cells, and multiple T-cell subsets (including CD8+ T cells, T helper cells, Tfhs, TILs and Th2 cells), whereas the High-TMI group displayed significantly reduced immune activation (Fig. 7A), and this pattern was validated in the GSE41613 cohort (Fig. S6A). Correlation analysis further demonstrated that some model genes (e.g. PTK2B, SPOCK2) were positively associated with immune cells and functions, while CTTN or PAK4 exhibited negative correlation, consistent with the overall negative correlation between TMI and immune activity (Fig. 7B; Fig. S6B-D).

CIBERSORT analysis confirmed these contrasts: Low-TMI tumors harbored higher proportions of dendritic cells resting, mast cells resting, T cells follicular helper, Tregs and T cells gamma delta, while High-TMI tumors were enriched in T cells CD4 memory resting and macrophages M1 (Fig. 7C–D). Prognostic analysis revealed that abundant mast cells resting predicted favorable survival, whereas elevated neutrophils or T cells CD4+ naive were linked to adverse outcomes (Fig. 7E–G). Integrating these features with TMI further refined prognosis: patients with low TMI and high mast cells resting survived significantly longer, while those with high TMI combined with neutrophils or T cells CD4+ naive had the worst prognosis (Fig. 7H–J).

Consistently, ESTIMATE analysis showed that Low-TMI tumors exhibited lower purity and higher immune/stromal scores, supporting an immune-rich phenotype, while High-TMI tumors were more inclined to immune-desert (Fig. 7K–N). Immune checkpoint analysis further highlighted differential expression patterns between High- and Low-TMI, namely LDHA or PVR positively correlated with TMI (*R* = 0.54; *R* = 0.43), suggesting a potential synergy of immune checkpoints with TMI in predicting immunotherapy response (Fig. 7O–Q). Intriguingly, immunotherapy response analysis revealed that Low-TMI patients had greater benefits than High-TMI patients, a finding validated in GSE35640 cohort and supported by TIDE analysis in TCGA and GSE41613 cohorts (Fig. 7R–S; Fig. S6E-F).

In summary, Low-TMI tumors represent an immune-rich, checkpoint-responsive microenvironment associated with better immunotherapy outcomes, whereas High-TMI tumors display immune suppression and worse prognosis.

### Potential significances of tumor metastasis-related index (TMI) in predicting drug sensitivity

**Fig. 8:**
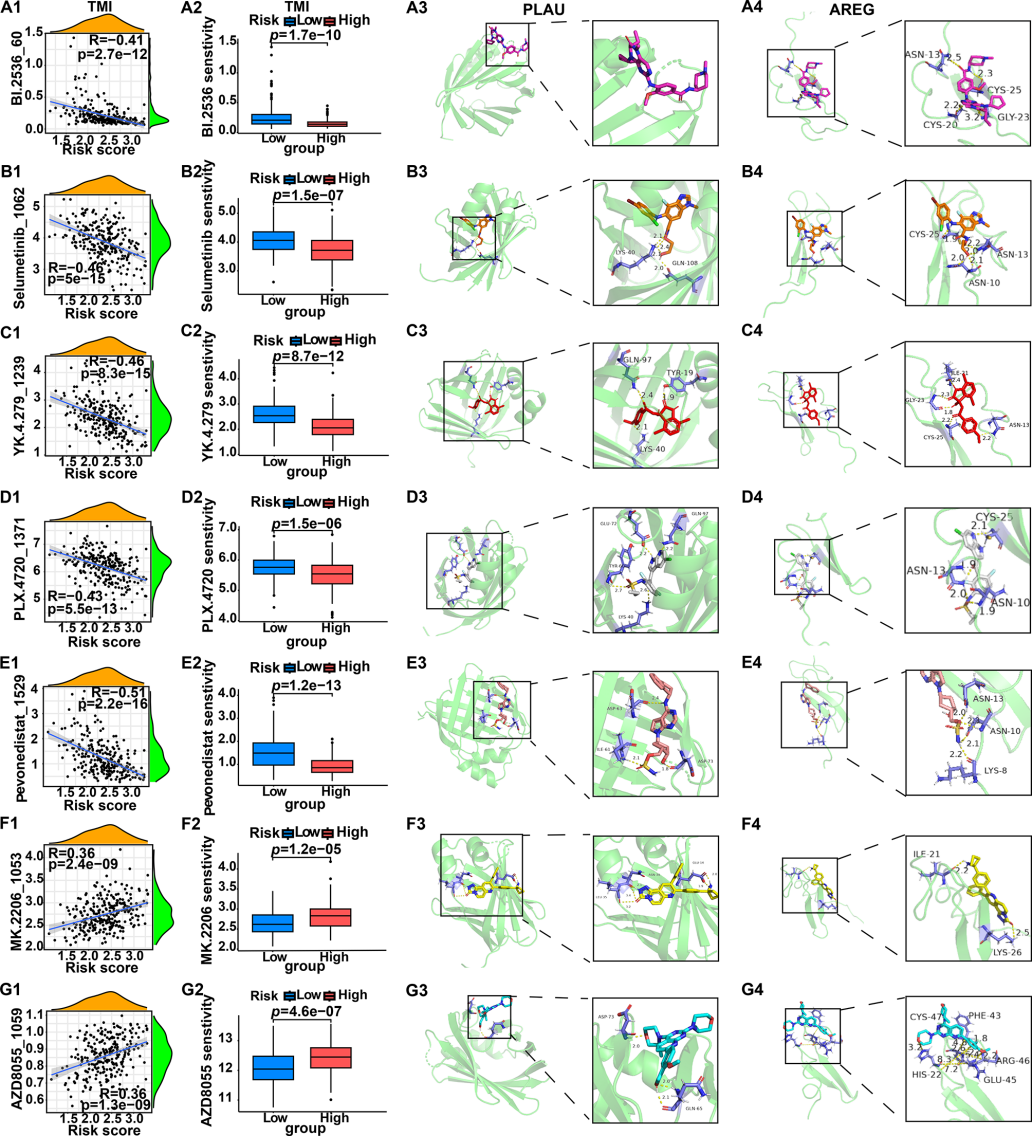
TMI-related drug sensitivity analysis and molecular docking. (**A1–G1**) correlation analysis between drug sensitivity and TMI. (**A2–G2**) differences of drug sensitivity between high-TMI and Low-TMI groups. (**A3–G3**) molecular docking results for PLAU with seven drugs. (**A4–G4**) molecular docking results for AREG with seven drugs. TMI, tumor metastasis index. Pearson correlation (**A1–G1**) and T-test (**A2–G2**) were used; *p* values were shown

To investigate the relationship between TMI and drug sensitivity, the drug response of OSCC samples from the GDSC1, GDSC2, and CTRP2 datasets was analyzed, where drug sensitivity was evaluated using IC50 values. Correlation analyses between TMI and drug sensitivity were conducted (Fig. 8A1-G1), and drug response distributions were compared between High- and Low-TMI groups (Fig. 8A2-G2). Five drugs were preferentially selected because their IC50 values were consistently negatively correlated with TMI across all three databases (Fig. 8A1-E1 and A2–E2), indicating that patients in the High-TMI group were more sensitive to these agents. Two additional drugs showed positive correlations with TMI in all databases (Fig. 8F1-G1 and F2–G2), suggesting greater sensitivity in Low-TMI patients. Thus, these seven agents were identified as candidate compounds for OSCC therapy, which might be potentially more effective to OSCC patients stratified by TMI.

The proteins encoded by two TMI signature genes (*PLAU* and *AREG*) were selected for visualization and analysis with the seven candidate drugs (Fig. 8A3-G3 and A4–G4) based on: 1) differential expression between normal and OSCC tissues (Fig. 3A); 2) significant prognostic value illustrated by Kaplan Meier survival curves (Figs. 3G and M); and 3) optimal binding energy under the above conditions. Docking analysis reveals that the binding energies of most drugs and genes are less than −5 kcal/mol (Table 3). In molecular docking, −5 kcal/mol suggests potential binding, while −7 kcal/mol indicates the stronger affinity. These findings provide quantitative evidences that the selected drugs not only correlate with TMI-defined sensitivity patterns but also exhibit strong binding to TMI-related proteins, thereby supporting their potential for therapeutic prioritization in OSCC.

**Table 3:** PDB IDs of 10 proteins encoded by risk genes and their binding energies with drugs

Gene symbol	PDB ID	Binding energy with drugs (kcal/mol)						
		BI.2536_60	Selumetinib_1062	YK.4.279_1239	PLX.4720_1371	Pevonedistat_1529	MK.2206_1053	AZD8055_1059
PLK1	8XB9	−7.05	−5.28	−6.76	−7.25	−6.41	−8.58	−6.27
PRKCA	4RA4	−8.41	−5.83	−7.87	−7.38	−7.34	−9.6	−8.02
MCL1	6O4U	−5.98	−6.51	−7.49	−7.76	−8.79	−7.62	−8.02
CTTN	2D1X	−7.26	−4.62	−6.06	−6.16	−6.57	−7.72	−6.62
PAK4	5VEF	−6.72	−6.07	−8.13	−7.2	−6.91	−8.64	−6.95
TGM2	2Q3Z	−8.05	−5.82	−6.91	−6.91	−7.36	−10.08	−7.69
PTK2B	8YGX	−8.29	−6.5	−6.83	−6.75	−6.55	−9.24	−7.56
PLAU	8GEM	−7.55	−7.2	−7.34	−7.41	−7.14	−7.9	−7.48
P4HA1	1TGC	−7.71	−4.04	−6.13	−5.43	−5.74	−7.94	−6.07
AREG	2RNL	−7.54	−5.47	−6.35	−6.57	−7.46	−8.19	−6.48

### Immunohistochemical analysis of signature genes

Based on immunohistochemistry (IHC) staining data from the Human Protein Atlas (HPA) database, the protein expression of TMI model genes was examined in normal oral tissues and head and neck squamous cell carcinoma (HNSCC) tissue samples (Fig. S6). This analysis confirmed that risk genes, including DDIT4, AREG, PLK1, PTK2B, PLAU, P4HA1, CTTN, MCL1 and PAK4, exhibited higher protein expression in OSCC tissues but lower expression in normal tissues (Fig. S6). Conversely, the protective gene SPOCK2 showed the elevated expression in normal tissues and reduced expression in OSCC tissues (Fig. S6). Consistent with our transcriptomic findings (Fig. 3A), these IHC results provide protein-level validation of this gene signature, which support the roles of risk genes in tumor progression and the potential tumor-suppressive effect of SPOCK2, thereby highlighting their relevance to OSCC prognosis and therapeutic stratification.

### Comparison of prognostic signatures in OSCC

**Fig. 9:**
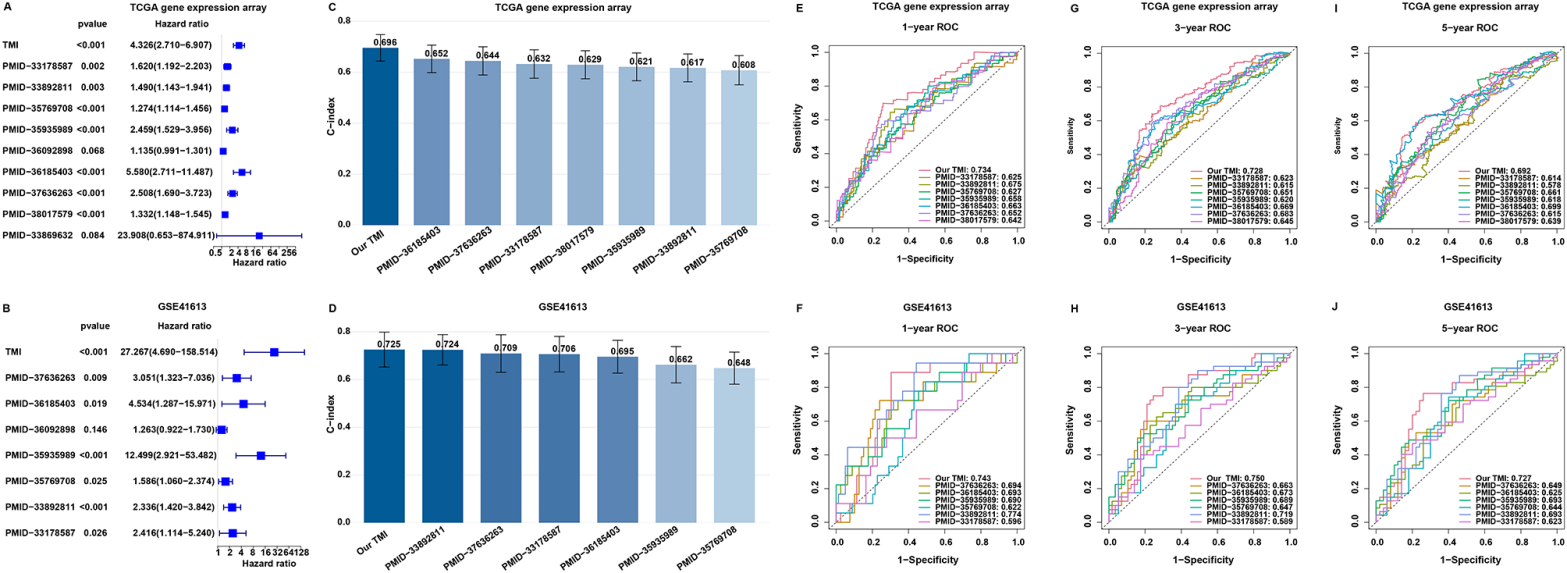
Comparison of prognostic models in TCGA and GSE41613 cohorts. (**A-B**) Forest plots showing HR and p-values of our TMI model and previously published prognostic signatures across the TCGA and GSE41613 datasets. (**C-D**) C-index values comparing predictive accuracy of each model in TCGA and GSE41613. (**E-J**) Time-dependent ROC curves evaluating the predictive performance of different models for 1-year (**E-F**), 3-year (**G-H**), and 5-year (**I-J**) overall survival in TCGA (**E, G, I**) and GSE41613 (**F, H, J**) datasets. HR, hazard ratios; C-index, concordance index

To demonstrate the superiority of TMI, we retrieved prognostic signatures for OSCC from published studies. For comparison with studies focused specifically on metastasis mechanisms, we included an anoikis-based signature [18, 31] derived from HNSCC samples; all other signatures were based on OSCC. Additionally, we incorporated an EMT-derived signature [32] along with others based on different mechanisms. Risk scores for these signatures were calculated strictly according to their published coefficients. Only signatures with significant univariate prognostic value were compared (Fig. 9A–B). TMI demonstrated better performance, higher C-index (Fig. 9C–D) and superior ROC results (Fig. 9E–J).

## Discussion

Oral squamous cell carcinoma (OSCC) is an aggressive and common cancer that leads to death. Cancerous metastasis is the most risky prognostic factor of OSCC as in other carcinomas [6, 11, 14, 33, 34]. This study integrated BM, EMT, and anoikis-related genes to construct TMI for OSCC using machine learning.

TMI outperforms the existing OSCC or HNSCC prognostic models in mechanism and predictive performance. Existing OSCC or HNSCC prognostic signatures, which often focus on only one or two biological processes [18, 32]. To capture the multi-step complexity of tumor metastasis, TMI was identified. By contrast, TMI targets three interconnected metastatic mechanisms, reflecting the sequential nature of OSCC dissemination: tumor cells break through the basement membrane [11], undergo EMT to acquire invasive ability [13–15], and resist anoikis to survive in the circulation [18, 19]. This metastatic mechanism endows TMI unique advantages in predicting metastatic risk. In terms of predictive performance, our model displayed more robust and beneficial performance than previous studies [32, 35–39]: AUC values at 1-year, 3-year and 5-year and C-index of our TMI were much higher than that in other models (Fig. 9). Decision curve analysis (DCA) further indicated that our TMI offered a greater net clinical benefit compared to clinical factors alone (Fig. 4E). Kaplan-Meier survival curves plotted by stratifying patients based on TNM, stage and neoplasm histologic grade can also illustrate the superiority of our TMI (Fig. S7).

Intriguingly, the signature genes of TMI (Fig. 2E–F), cooperatively regulate tumor metastasis (Fig. S8). Basement membrane and ECM degradation: PLAU (plasminogen activator urokinase) facilitates the degradation of extracellular matrix (ECM) and basement membrane [40], while PLK1 promotes cytoskeletal reorganization and ECM degradation [41]. EMT induction and invasive ability acquisition: EMT enables OSCC cells to shift from an epithelial to a mesenchymal phenotype, enhancing their migratory capacity and facilitating detachment from the primary tumor [42]. Hypoxia-induced P4HA1 activates the TGF-β/Smad pathway and up-regulates EMT-related transcription factors [43]. Furthermore, the TGF-β pathway regulates the alternative splicing of *CTTN* to contribute to EMT [44]. The *CTTN*-encoded protein cortactin promotes actin polymerization, which enhances cancer cell migration and invasion [45]. Furthermore, TGM2 can modulate EMT through activation of the NF-κB pathway [46], and PTK2B is also involved in regulating EMT in HNSCC (including OSCC) [47]. Anoikis resistance: to survive after detaching from the primary site, cancer cells must evade anoikis [18, 19]. In HNSCC (including OSCC), AREG is frequently up-regulated and binds to EGFR, thereby activating the MAPK/ERK and PI3K/Akt pathways [48, 49]. Similarly, PRKCA and PAK4 activate these pathways [50, 51], leading to up-regulation of anti-apoptotic proteins (such as MCL1) and suppression of pro-apoptotic proteins, thereby conferring anoikis resistance [49, 52]. Meanwhile, under hypoxic conditions, *DDIT4* expression is up-regulated to inhibit the mTOR pathway in HNSCC, leading to the reduced protein synthesis and cell proliferation. This metabolic adaptation helps cancer cells survive under stress conditions during metastasis, thereby facilitating subsequent proliferation and invasion [53]. It has been reported that sustained *DDIT4* overexpression, inhibits mTORC1, but activates mTORC2-mediated Akt phosphorylation, a key survival signal, ultimately promoting tumor cell survival and chemoresistance [54]. This explains why these signature genes can synergistically predict metastasis. They target critical steps in the process of tumor dissemination (Fig. S8). Studies have shown that AREG, CTTN, PRKCA, MCL1, PAK4, PLAU and PLK1 are all implicated in OSCC or HNSCC progression and inclined to poor prognosis [40, 41, 48, 51, 55–57], suggesting that these genes may also serve as promising targets for molecular targeted therapy. The mutually exclusive mutations between *TP53* and *CASP8*, and co-occurring mutations between *TP53* and *CDKN2A*, further support the genetic basis of tumor metastasis: *TP53* mutation disrupts cell cycle checkpoints [58, 59], while *CDKN2A* loss enhances cell proliferation [60]. Additionally, high-frequency amplification of *CTTN* ( > 50%) and copy number loss of *PTK2B* ( > 40%) (Fig. 6F–G) suggest that genetic variations drive abnormal gene expression to reinforce metastasis.

GSEA and GSVA enrichment analyses identified VEGF, TGF-β and JAK-STAT signaling pathways were enriched in the High-TMI group. This finding provided a mechanistic explanation for its malignant phenotype. Activation of the VEGF pathway can accelerate the colonization of metastatic foci by promoting tumor angiogenesis [61]. P4HA1 can regulate the expression of vascular endothelial growth factor A (VEGF-A), suggesting that P4HA1 may be involved in the VEGF signaling pathway [62]. As a core regulator of EMT, TGF-β directly drives the enhancement of the invasive ability of tumor cells [63, 64]. The TGF-β pathway can also participate in the EMT process by regulating the alternative splicing of *CTTN* (cortactin) [44]. Under hypoxic conditions, *P4HA1* expression is markedly up-regulated. This up-regulation promotes EMT and enhances invasiveness in OSCC cells through activation of the TGF-β/Smad pathway [43]. The abnormal activation of the JAK-STAT pathway promotes the metastasis process by regulating cell proliferation and the inflammatory microenvironment [65]. PTK2B (Pyk2) can be activated by CCR7 in HNSCC. Meanwhile, CCR7 relies on JAK2/STAT3 to regulate the metastasis and survival of HNSCC. Therefore, it is speculated that PTK2B and the JAK-STAT pathway may be in the same signaling network mediated by CCR7, working together to promote tumor progression [66]. Besides, correlation analysis between risk genes and pathways showed that *P4HA1* and *PTK2B* were significantly positively correlated with the TGF-β and JAK-STAT signaling pathways, respectively (*R* > 0.4, *p* < 0.05) (Fig. S9), suggesting their potential roles in the regulation of these signaling pathways and related biological processes (Fig. S8). The effects of these three pathways may confirm the phenotypic characteristic of “high metastasis risk in the High-TMI group” and further support the rationality of TMI as a molecular indicator for OSCC metastasis. Notably, the ion channel-related pathways, such as potassium and sodium channels, enriched in the Low-TMI group might provide a perspective for OSCC treatment. Abnormal functions of ion channels can affect tumor progression by regulating cell migration ability [67]. Their specific enrichment in the Low-TMI group suggests that targeting ion channels may become a potential strategy to inhibit OSCC metastasis.

This study also revealed a close link between TMI and tumor immune infiltration in OSCC. This helps explain how metastasis and immune escape cooperate, potentially improving immunotherapy. Tumor immune microenvironment analysis showed major differences between High- and Low-TMI groups. In particular, the Low-TMI group was enriched with immune cells, including mast cells, follicular helper T cells, resting dendritic cells and Tregs. In the Low-TMI group, high levels of mast cells resting were associated with better prognosis, consistent with previous reports that mast cells fight tumors via histamine and IL-6 [68]. They may strengthen immune surveillance in OSCC [69]. T cells follicular assists B cells to boost anti-tumor immunity [70]. Existing studies have confirmed that in HNSCC, the enrichment of follicular helper T cells is significantly associated with the formation of intratumoral germinal centers, the production of high-affinity anti-tumor antibodies, and the prolonged patient survival [71, 72]. Dendritic cells resting preserves antigen presentation capacity, enabling uptake and presentation of new tumor antigens to naive T cells. This sustains long-term immune memory and reduces recurrence risk [73]. Tregs typically exhibit immunosuppressive properties, however, studies have demonstrated that the function of Tregs in OSCC is dependent on tumor stage and microenvironmental context [74]. Specifically, in early-stage or HPV-positive OSCC, Tregs can reduce immune exhaustion either by suppressing excessive inflammation or synergizing with follicular helper T cells to regulate B-cell production of anti-tumor antibodies [75]. Together, these cells form a tumor-restraining microenvironment. In contrast, High-TMI group had high purity, low ESTIMATE scores, and were dominated by T cells CD4 memory resting and macrophages M1. The enriched resting memory CD4^+^ T cells might be functionally exhausted, failing to effectively initiate anti-tumor immunity. Although high infiltration of M1 macrophages is traditionally considered to have anti-tumor activity [76, 77], their dominance in the High-TMI group may be linked to tumor cell-induced phenotypic switching, specifically, by secreting pro-inflammatory factors to promote angiogenesis or stromal remodeling, thereby accelerating metastatic progression [78]. This may be one of the key reasons for the higher malignancy of the High-TMI group. Moreover, two immune checkpoint molecules, PVR and LDHA, were up-regulated in High-TMI patients and positively correlated with TMI (*R* = 0.43; *R* = 0.54). PVR binds TIGIT on T and NK cells to suppress T-cell activation, impair NK cytotoxicity, alter cytokine secretion, and promote immune escape [79, 80]. LDHA is a key enzyme in aerobic glycolysis. Its overexpression in High-TMI tumors may lead to lactate accumulation in the microenvironment, directly suppressing the proliferation and cytotoxic function of CD8^+^ T cells and NK cells [81]. Besides, the acidic & lactate-rich TME may reduce response to immune checkpoint inhibitors (ICIs) in High-TMI group. These results suggest that TMI could stratify patients for immunotherapy in a TMI-specific manner: Low-TMI patients may benefit more from ICIs; High-TMI patients may require combination therapies, such as anti-PD-1/PD-L1 together with LDHA or PVR inhibitors.

Currently, chemotherapeutic drugs such as doxorubicin (Dox) and 5-fluorouracil (5-FU) are commonly used in the clinical treatment of oral squamous cell carcinoma (OSCC). However, these drugs are associated with severe side effects and drug resistance. Meanwhile, newly developed drugs such as sanguinarine are still far from clinical application [82]. Therefore, the development of promising drugs targeting OSCC is imperative to address the challenges related to chemotherapy. Furthermore, the relationship between TMI and drug sensitivity was also explored, and then 7 drugs that were significantly with TMI were selected. It is worth noting that AZD8055, as an mTOR inhibitor, has shown anti-tumor potential by inhibiting mTORC1 and mTORC2, and its application research involves a variety of cancers such as bladder cancer, colon cancer and cervical cancer [83], but has not yet involved OSCC. Current research and development of anti-tumor drugs would focus on “multi-target synergy”. For instance, Sorafenib (a multi-target inhibitor acting on Raf, VEGFR and PDGFR) and Dasatinib (a multi-target inhibitor targeting Bcr-Abl and Src) both improve therapeutic efficacy by interfering with signaling pathway networks [84, 85]. Moreover, MCL1 exhibits critical crosstalk with the mTOR pathway: mTORC1 regulates MCL1 synthesis via 4E-BP1 [86], and high MCL1 expression in turn leads to resistance to mTOR inhibitors [87], forming an “mTOR-MCL1 axis”. This provides a theoretical support for the possibility that high-risk patients may respond to AZD8055 well. The pathogenic mechanism of the High-TMI group included the mTOR signaling pathway, and the High-TMI patients were more sensitive to AZD8055, which indicates that AZD8055 may have a better potential therapeutic effect in OSCC patients with High-TMI. *PLAU* and *AREG* are highly expressed in OSCC tissues and regulate key metastatic processes: PLAU can promote basement membrane degradation [40], while AREG can activate the EGFR-MAPK/PI3K pathway to induce anoikis resistance [48]. The seven candidate drugs exhibited strong binding affinity with proteins encoded by these two genes, which supports their potential as candidate regimens for targeted therapy of OSCC.

External validation consistently confirmed the reliability of TMI. In the GSE41613 cohort, TMI stratified patients into two groups with significant survival differences (*p* < 0.001) and recapitulate the immune infiltration patterns of the TCGA cohort. Immunohistochemical results based on the HPA database validated the differential expression patterns of signature genes at the protein level: risk genes (such as *DDIT4* and *AREG*) were highly expressed in cancer tissues and weakly expressed in normal tissues, whereas protective genes, particularly *LAMC3*, showed the opposite trend. And these results were consistent with above findings at the transcriptome level, reinforcing their potential roles in cancer/tumor progression: the high expression of risk genes may promote the development of OSCC through their functions in enhancing cell proliferation and invasion, while the low expression of protective genes may participate in carcinogenesis by disrupting basement membrane integrity and other mechanisms.

Here, we integrated basement membrane, EMT and anoikis-related genes to construct TMI model for OSCC risk prediction using machine learning. It provides multi-dimensional application value for the clinical management of OSCC. A nomogram integrating TMI, age and smoking status can predict 1-, 3- and 5-year survival rates. Patients with low nomogram score have low tumor metastasis potential and favorable prognosis, so overtreatment would be avoided. Patients with high nomogram score show high metastasis risk and worse prognosis, requiring intensive treatment. The TMI also guides targeted drug selection based on TMI stratification. The High-TMI group is sensitive to drugs such as AZD8055. The Low-TMI group is more sensitive to drugs such as BI.2536_60. Additionally, the TMI might optimize immunotherapy strategies. The Low-TMI group has an immune-enriched microenvironment and high response rate to immune checkpoint inhibitors (ICIs). The High-TMI group has high expression of LDHA and PVR, which reduces its response to ICIs. Combined therapy may be beneficial for OSCC patients stratified by TMI, such as anti-PD-1/PD-L1 combined with LDHA or PVR inhibitors. For clinicians and researchers, TMI will be detected by qPCR or IHC to validate this model and stratify OSCC patients. The association between TMI and treatment response will be analyzed to lay the foundation for multi-center randomized controlled trials (RCTs). And combined therapy will be conducted in a TMI-specific manner.

## Conclusion

We have established TMI associated with OSCC metastasis based on a combination of machine learning algorithms for evaluating the progression, prognostic risk, immunotherapy and treatment response of OSCC, which exhibited strong prediction performance. Thus, it may provide potential biomarkers for the clinical evaluation and molecular targeted therapy of OSCC patients.

## Electronic supplementary material

Below is the link to the electronic supplementary material.

Supplementary Material 1

Supplementary Material 2

Supplementary Material 3

Supplementary Material 4

Supplementary Material 5

Supplementary Material 6

Supplementary Material 7

Supplementary Material 8

Supplementary Material 9

Supplementary Material 10

Supplementary Material 11

Supplementary Material 12

Supplementary Material 13

Supplementary Material 14

Supplementary Material 15

## Data Availability

Gene expression profiling and survival information of OSCC was obtained from The Cancer Genome Atlas (TCGA, https://portal.gdc.cancer.gov/) and the Gene Expression Omnibus (GEO) database (https://www.ncbi.nlm.nih.gov/gds/?term=) (GSE41613).
