# Supplementary figures and images for: Development of a cancer metastasis-associated risk model via multi-machine-learning algorithms for prognostic risk evaluation and clinical application in oral squamous cell carcinoma
Source: J Transl Med. 2025 Nov 24;23:1344. doi: 10.1186/s12967-025-07336-y (PMC12645686; doi:10.1186/s12967-025-07336-y)

**A**Type ■ Normal ■ Cancer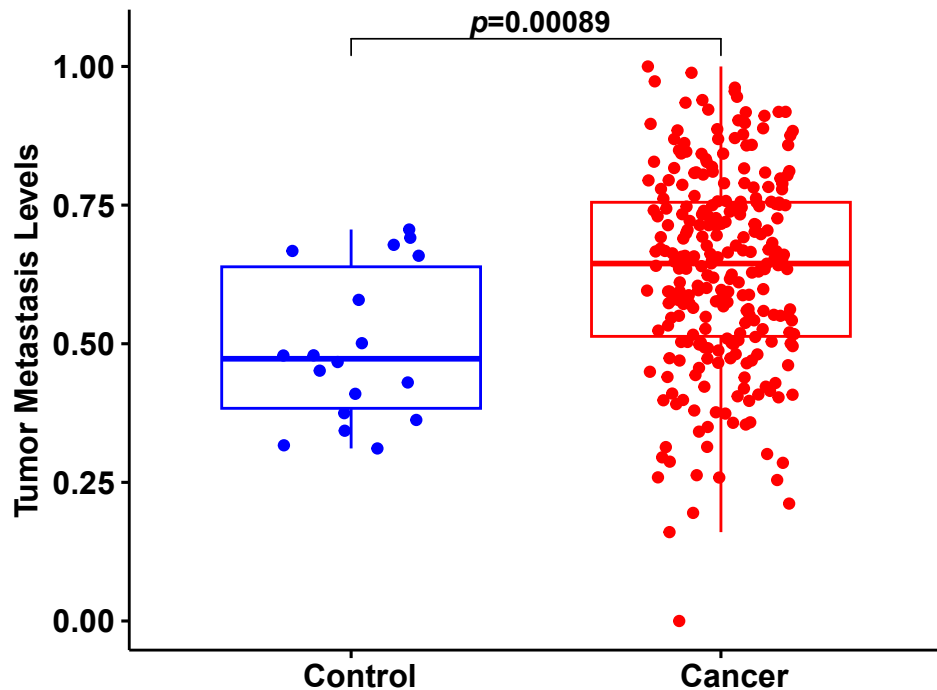**B**

ssGSEA-ROC

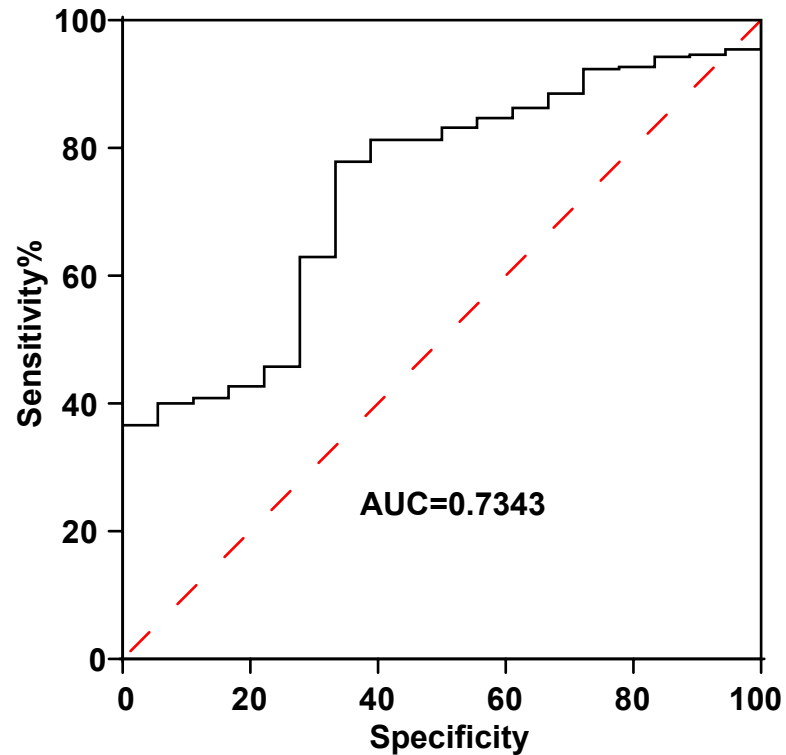

Supplement: Supplementary file 1 — Supplementary Material 1 [file 12967_2025_7336_MOESM1_ESM.pdf]

## Genes selected by each model

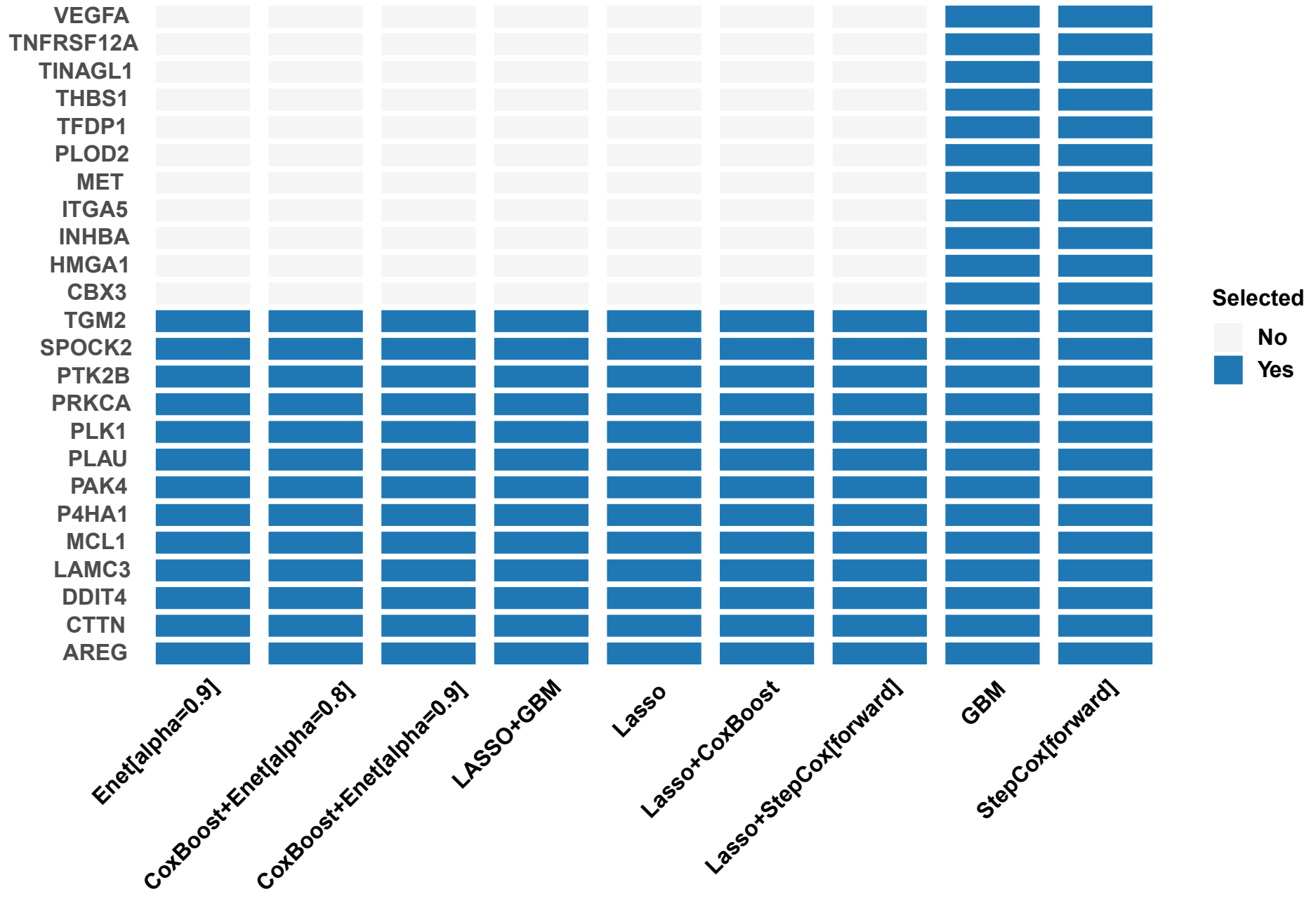

Supplement: Supplementary file 2 — Supplementary Material 2 [file 12967_2025_7336_MOESM2_ESM.pdf]

Risk    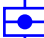 High    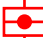 Low

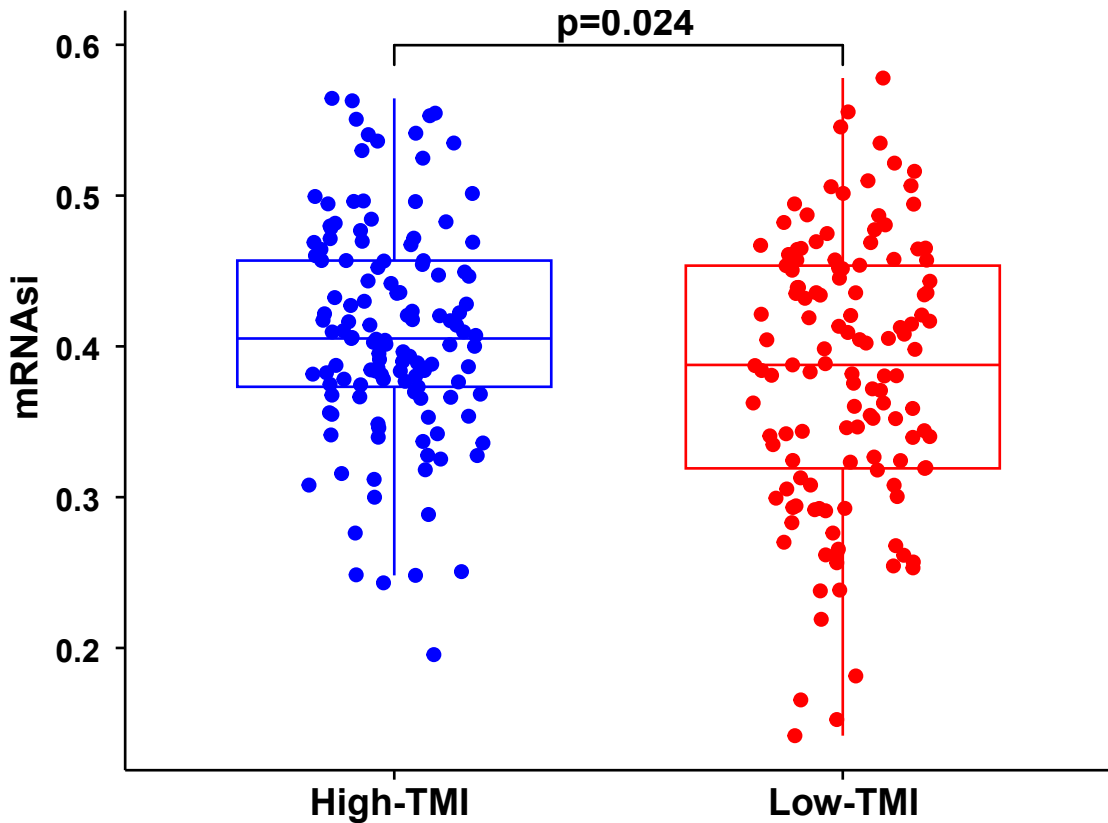

Supplement: Supplementary file 3 — Supplementary Material 3 [file 12967_2025_7336_MOESM3_ESM.pdf]

# GSE275870

Tissue ▢ metastasis of the lymph node ▢ primary tumor

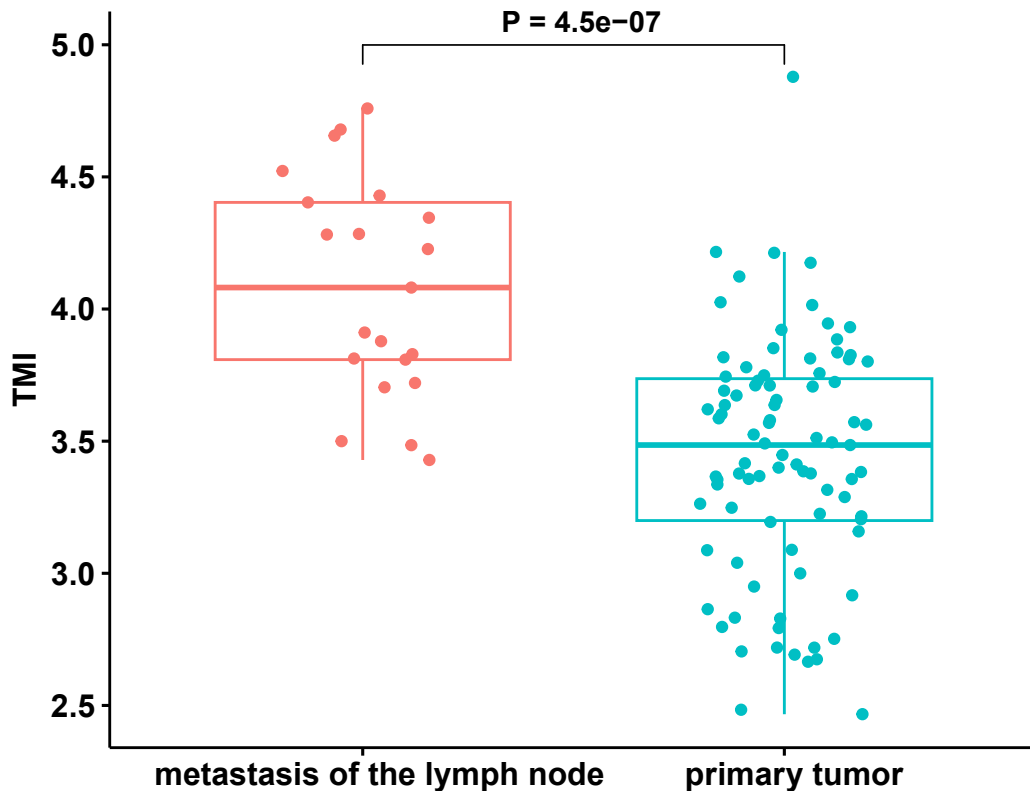

Supplement: Supplementary file 4 — Supplementary Material 4 [file 12967_2025_7336_MOESM4_ESM.pdf]

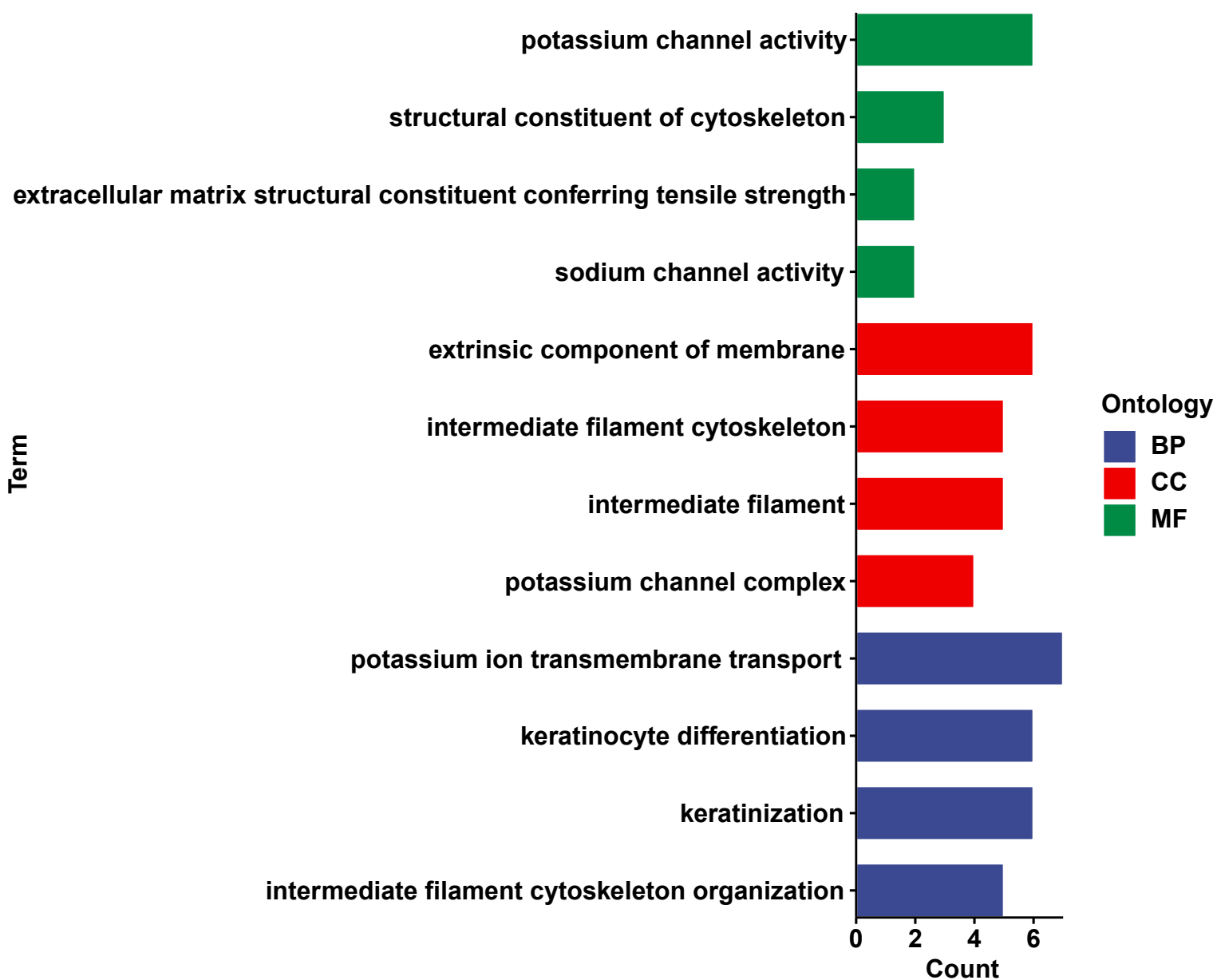

Supplement: Supplementary file 5 — Supplementary Material 5 [file 12967_2025_7336_MOESM5_ESM.pdf]

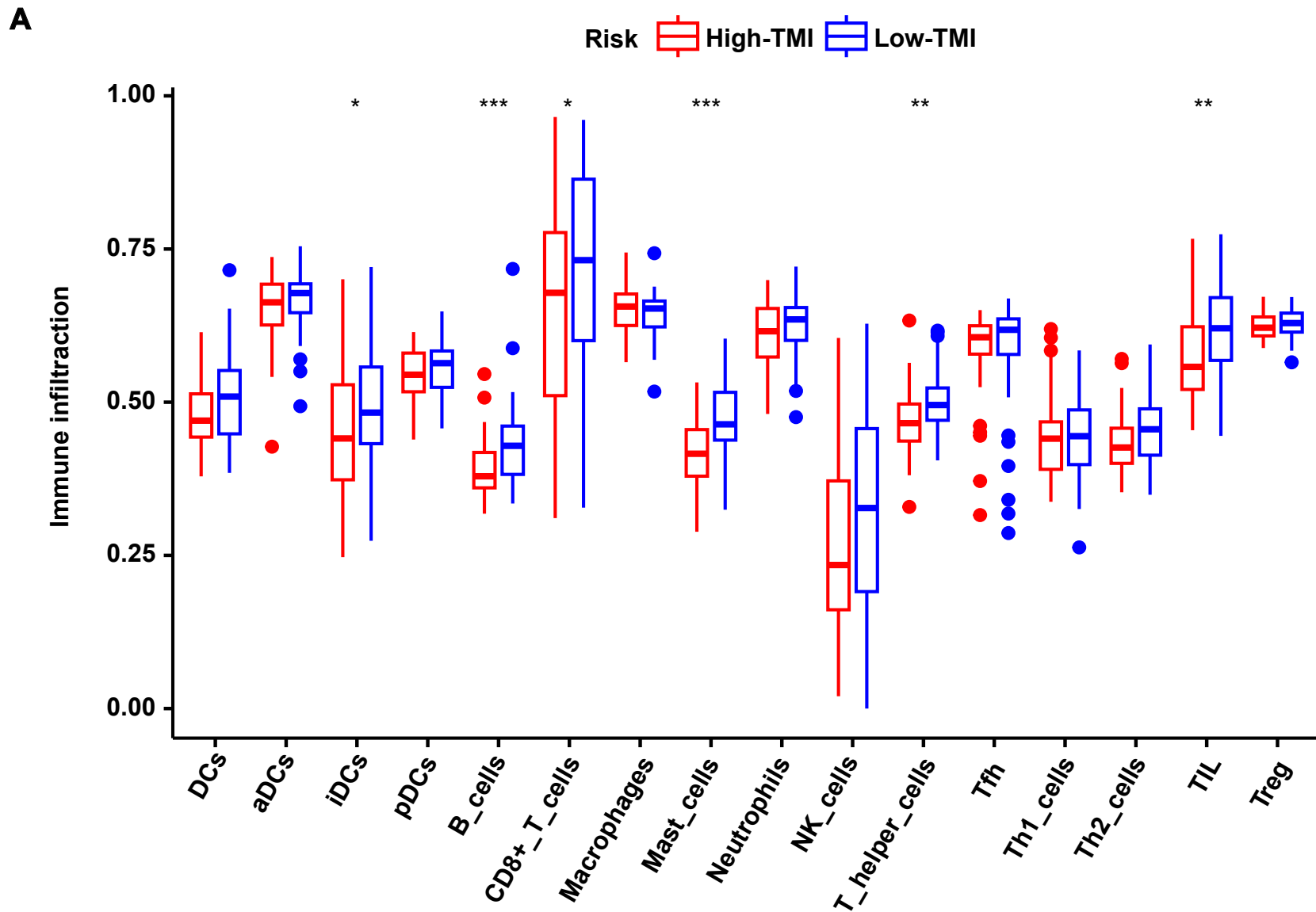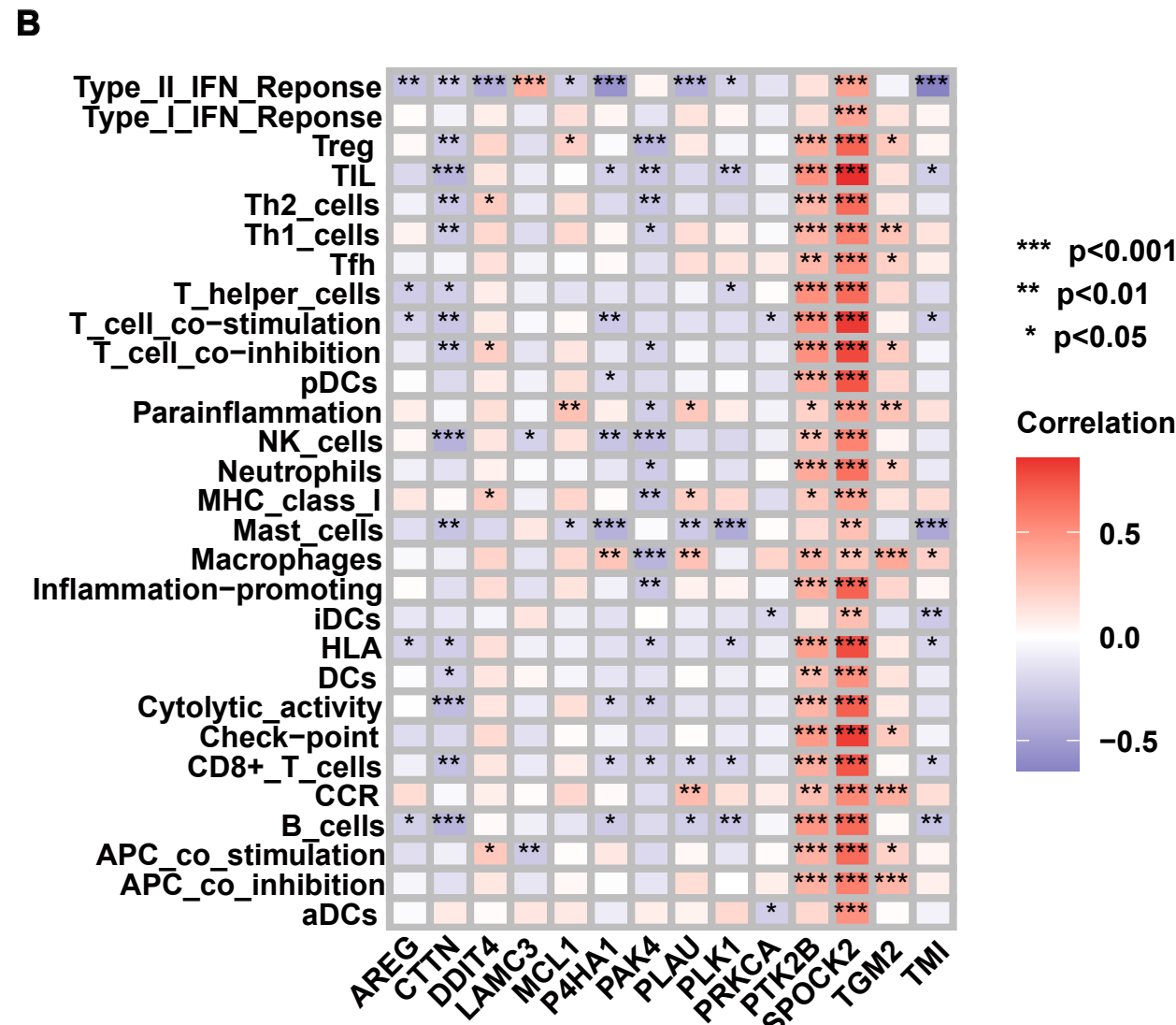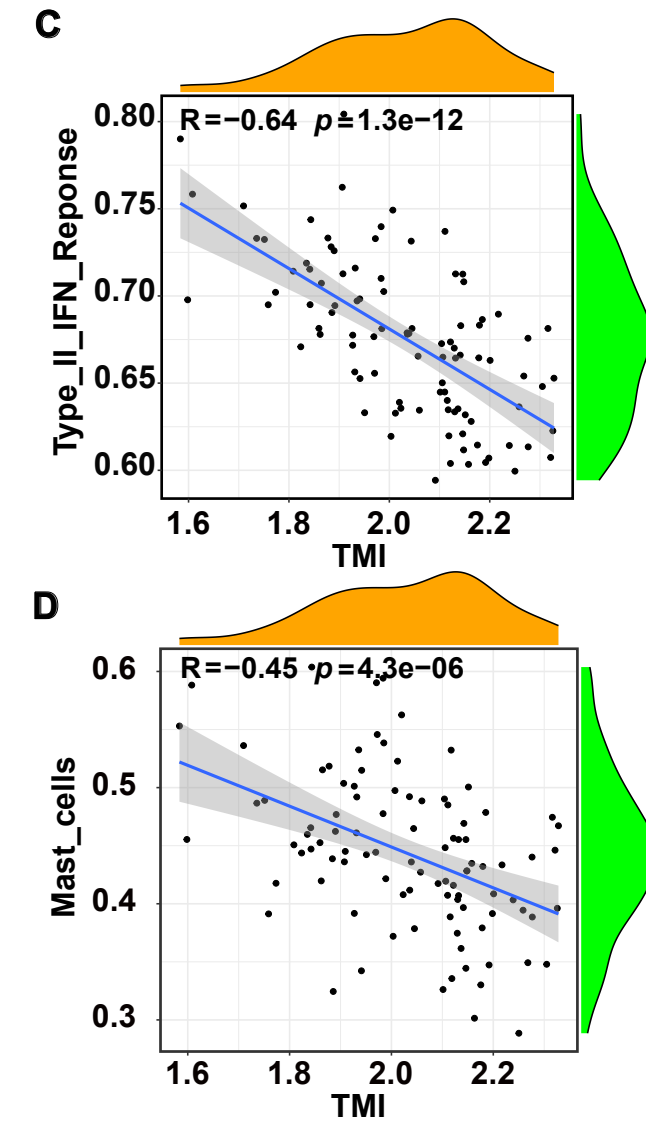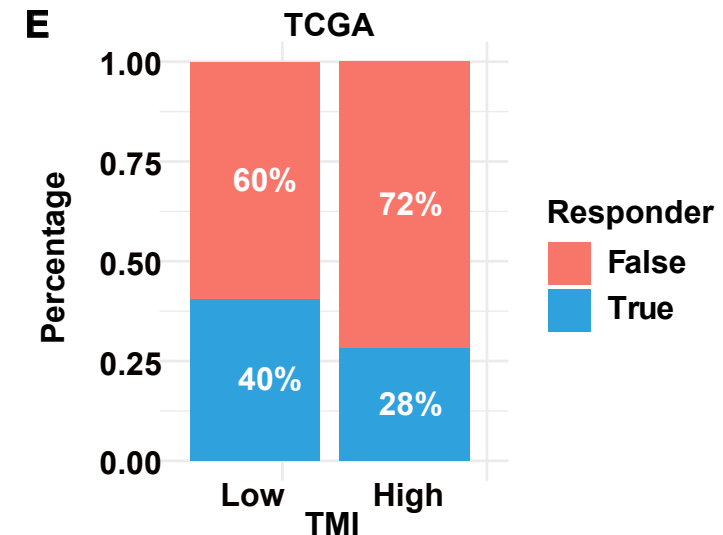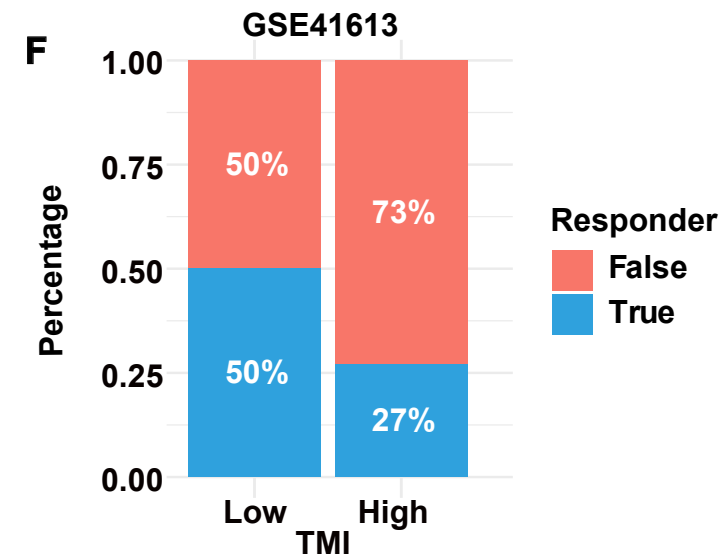

Supplement: Supplementary file 6 — Supplementary Material 6 [file 12967_2025_7336_MOESM6_ESM.pdf]

Cancer

DDIT4

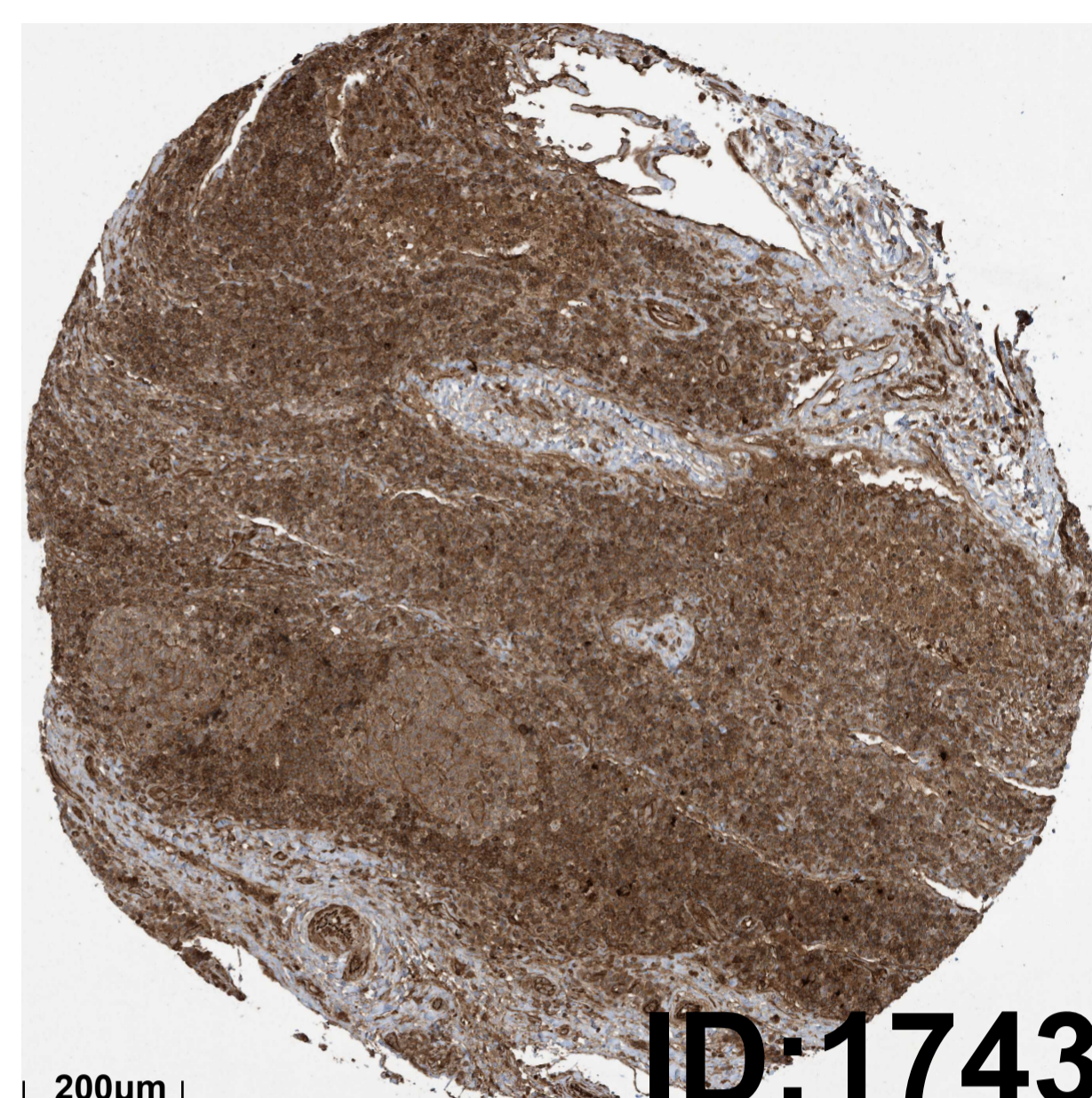

AREG

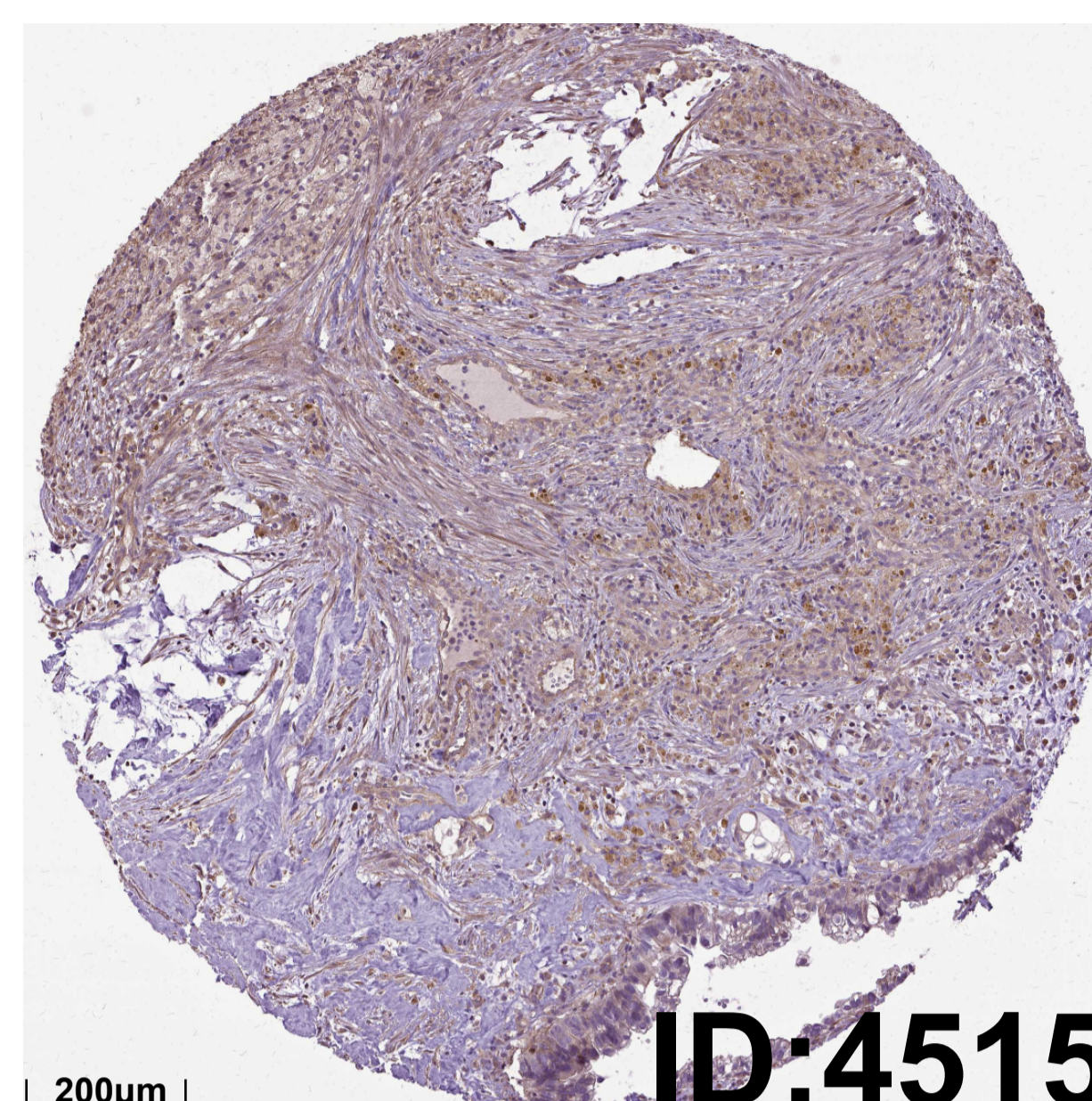

PLK1

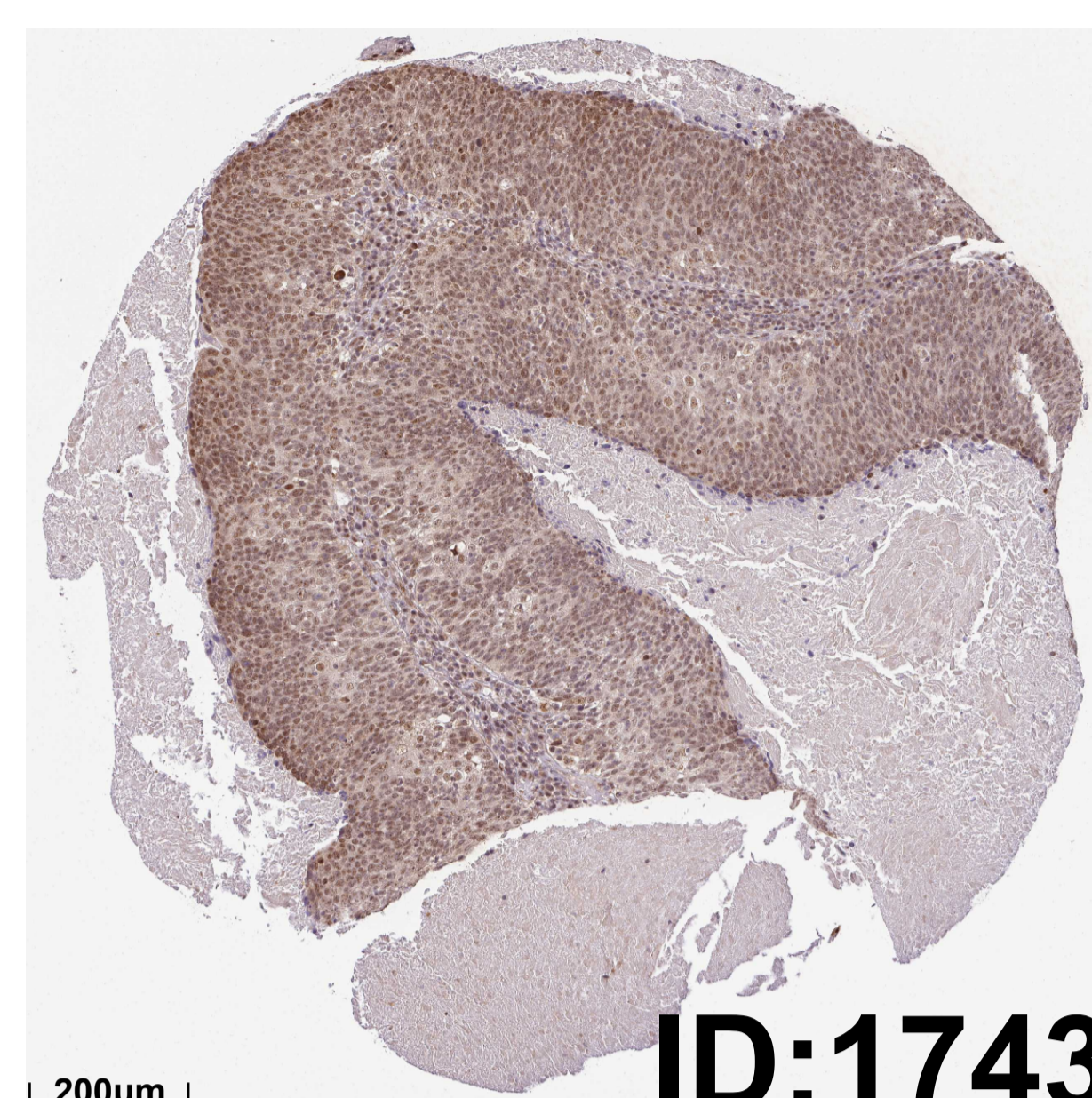

TGM2

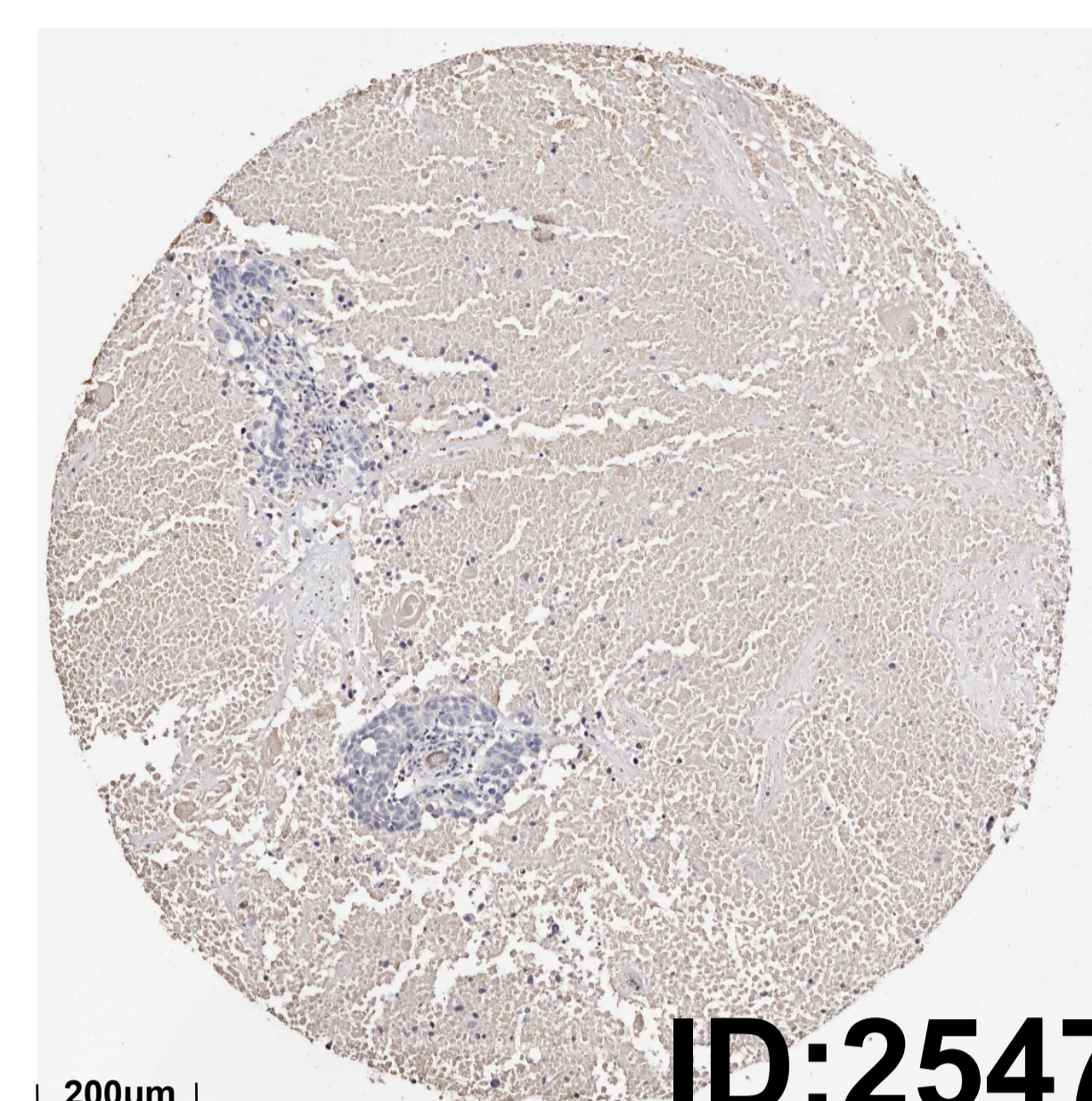

PTK2B

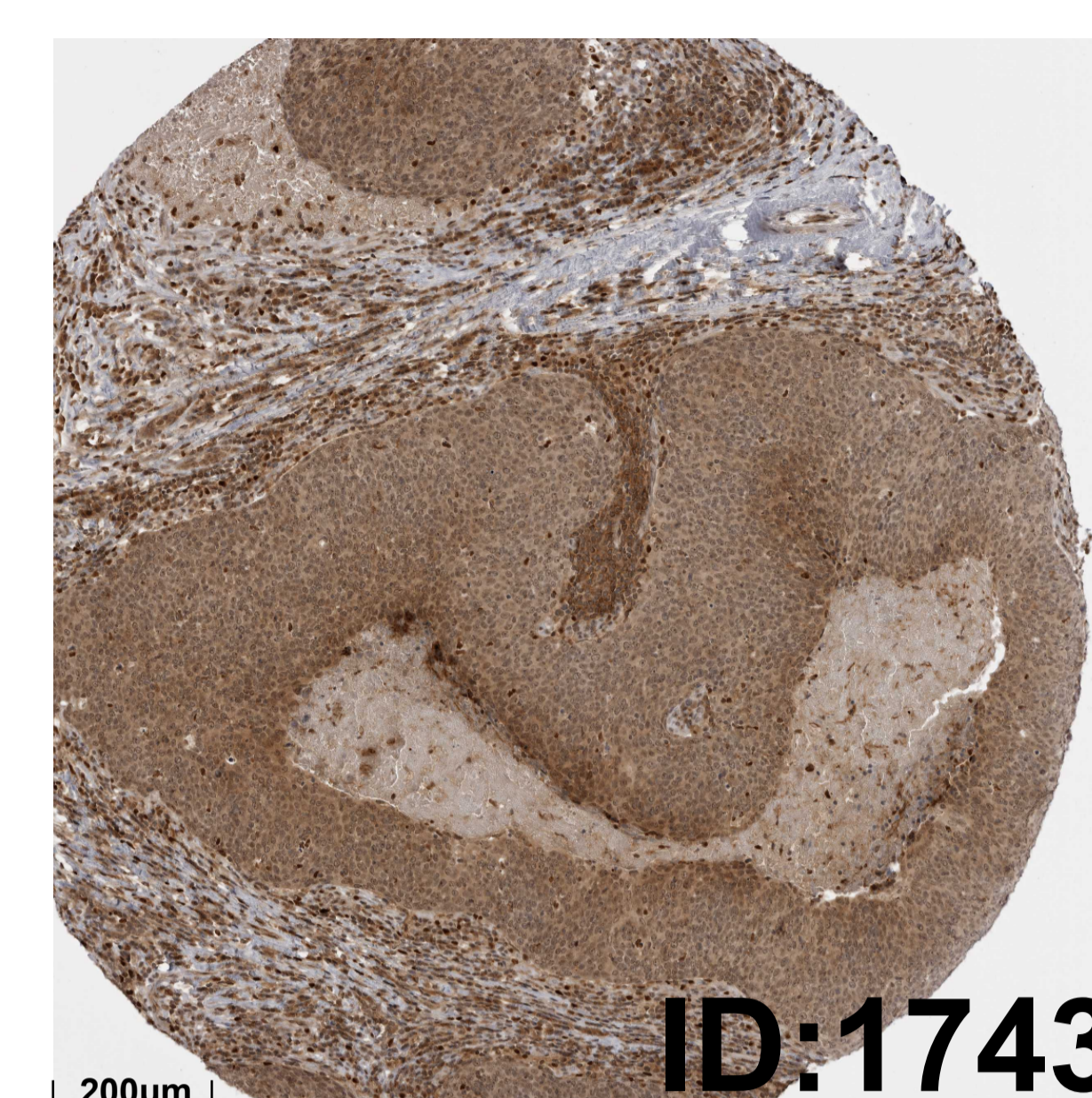

PLAU

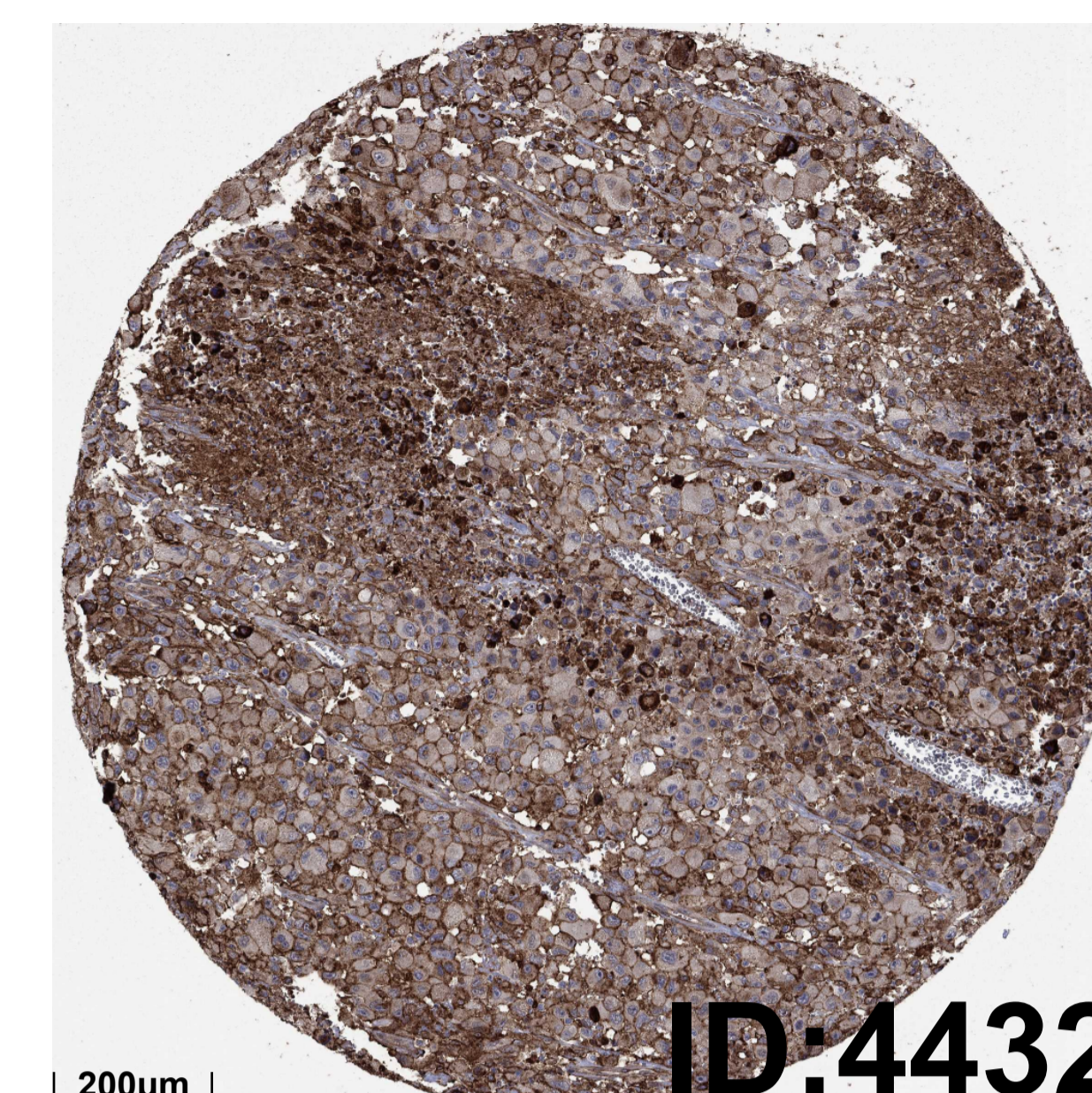

Normal

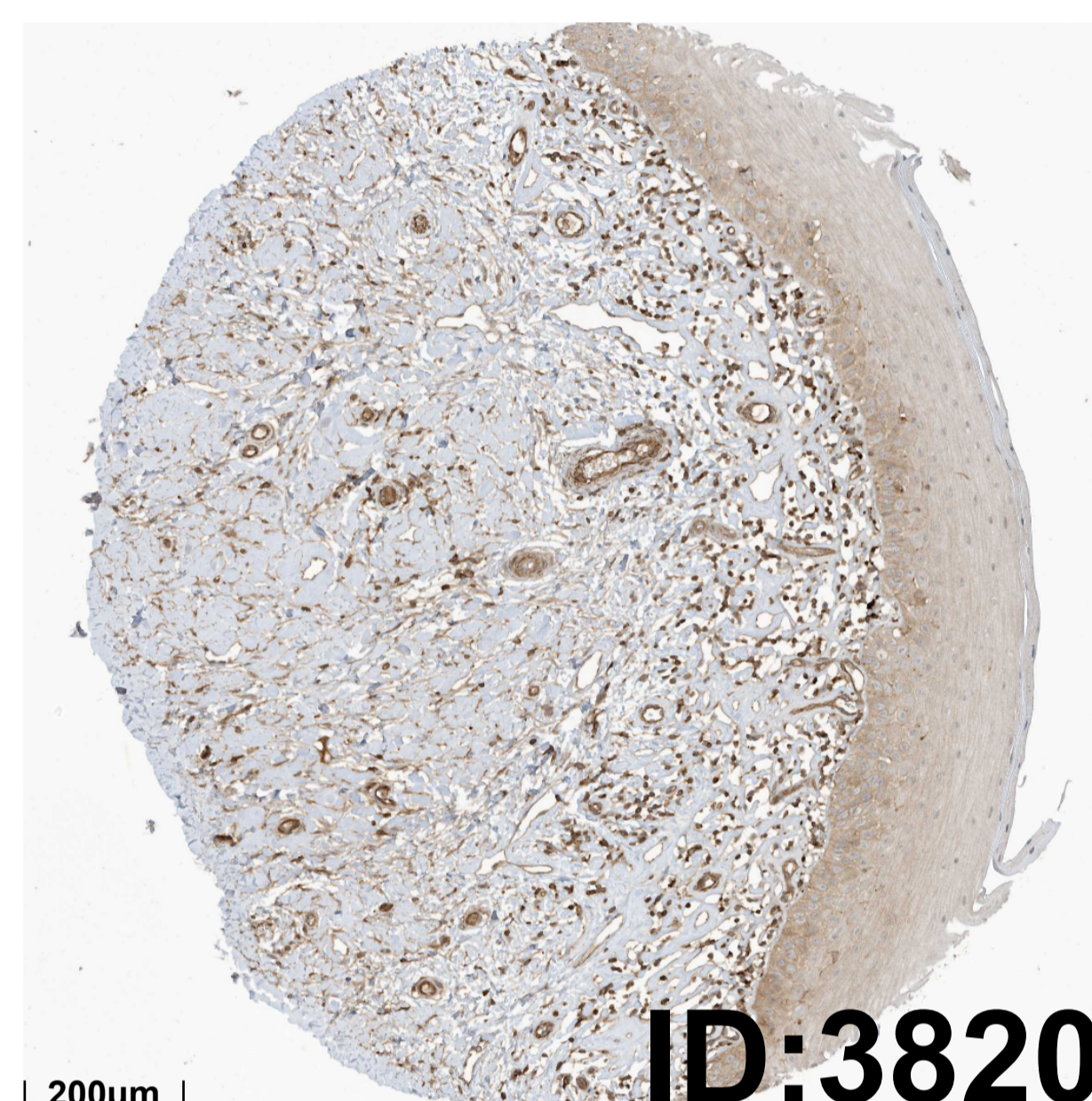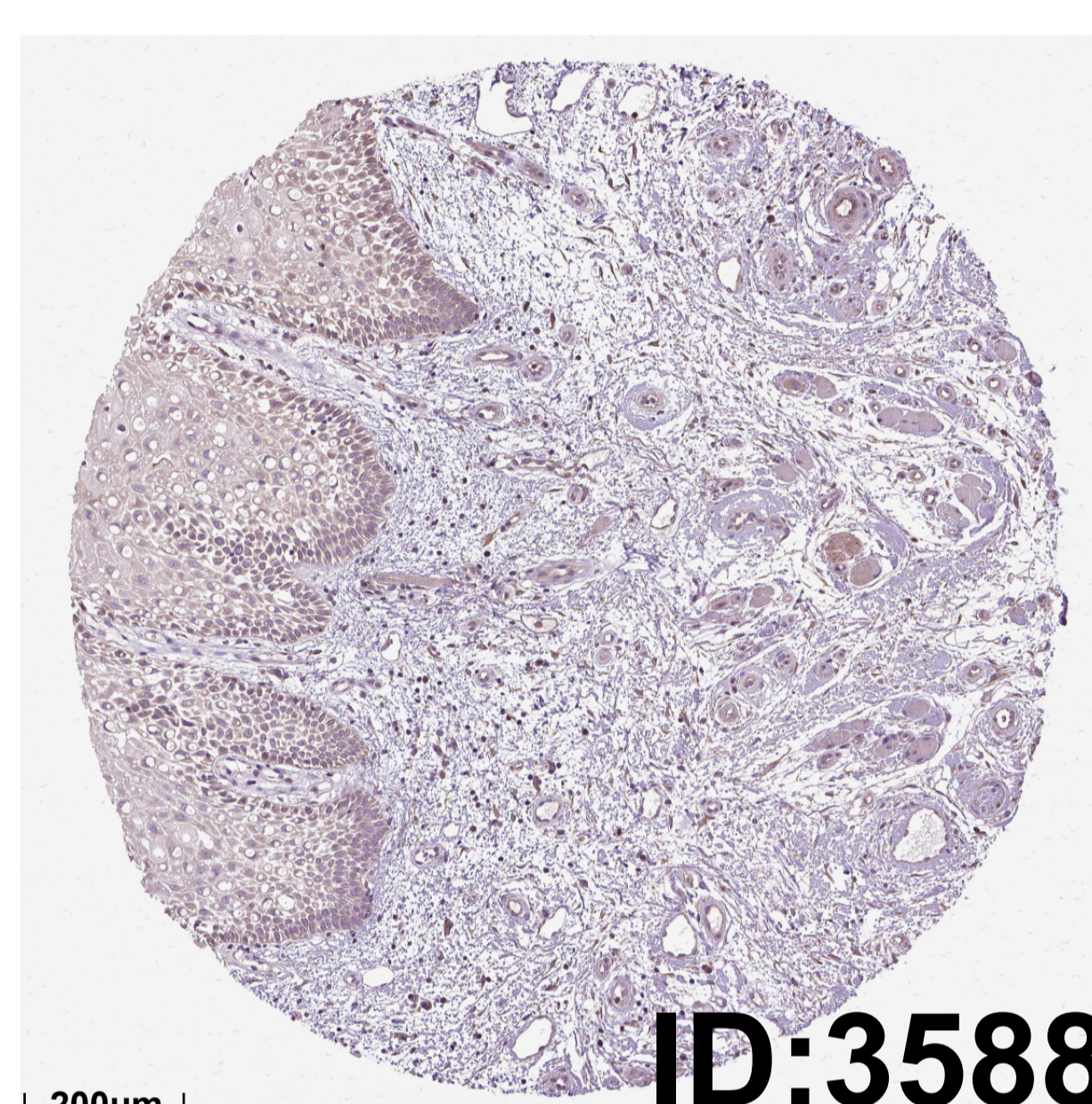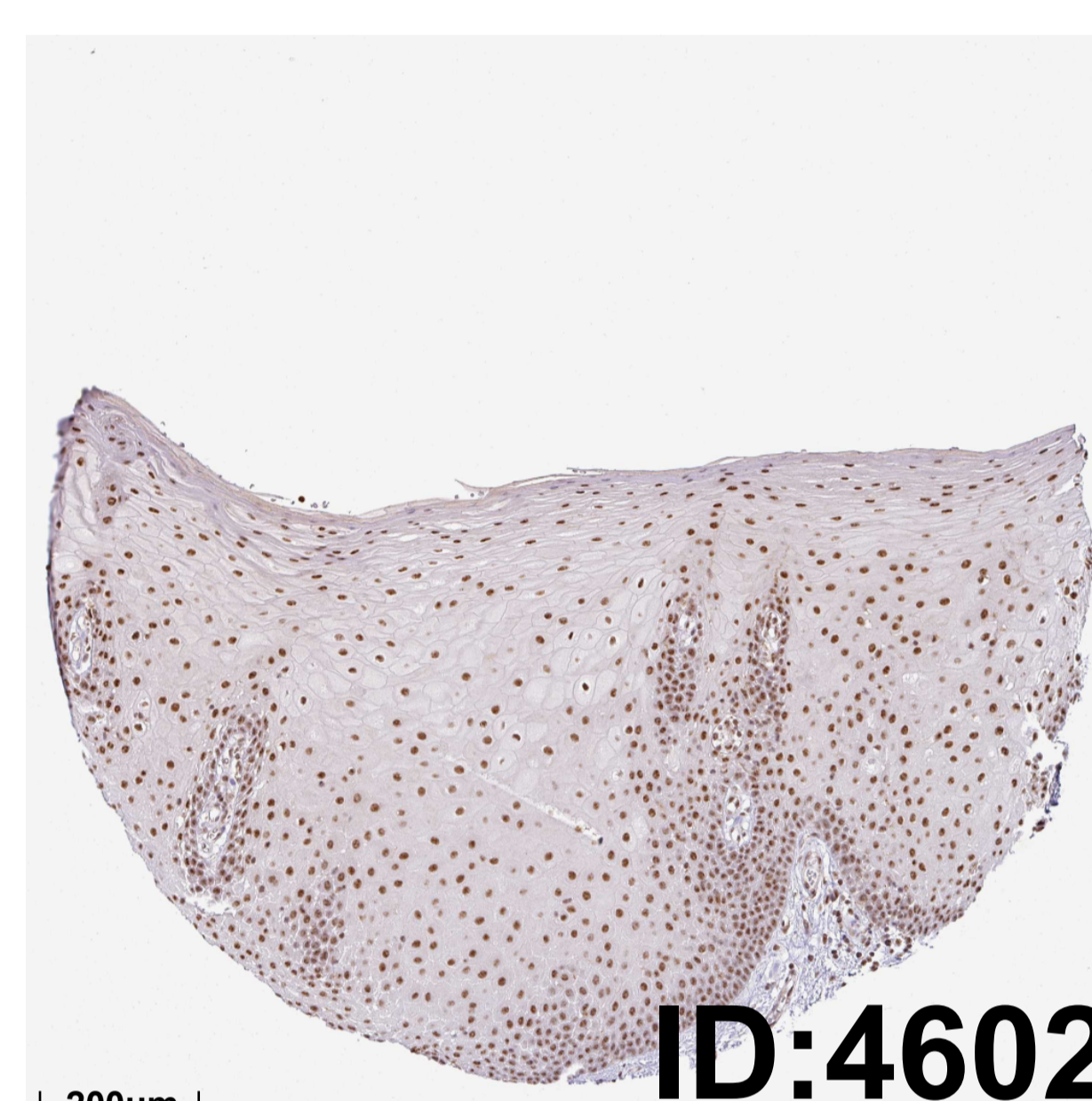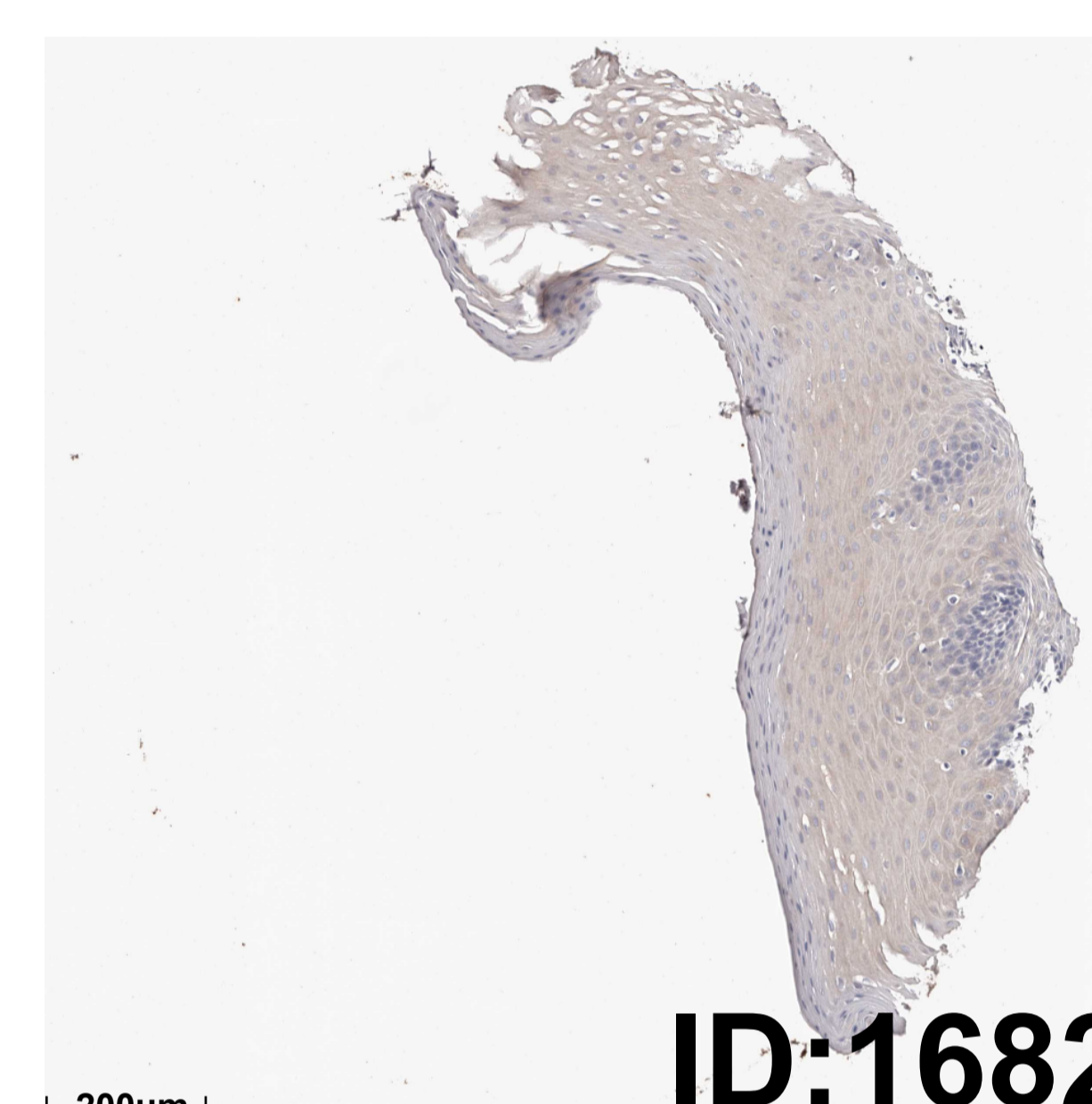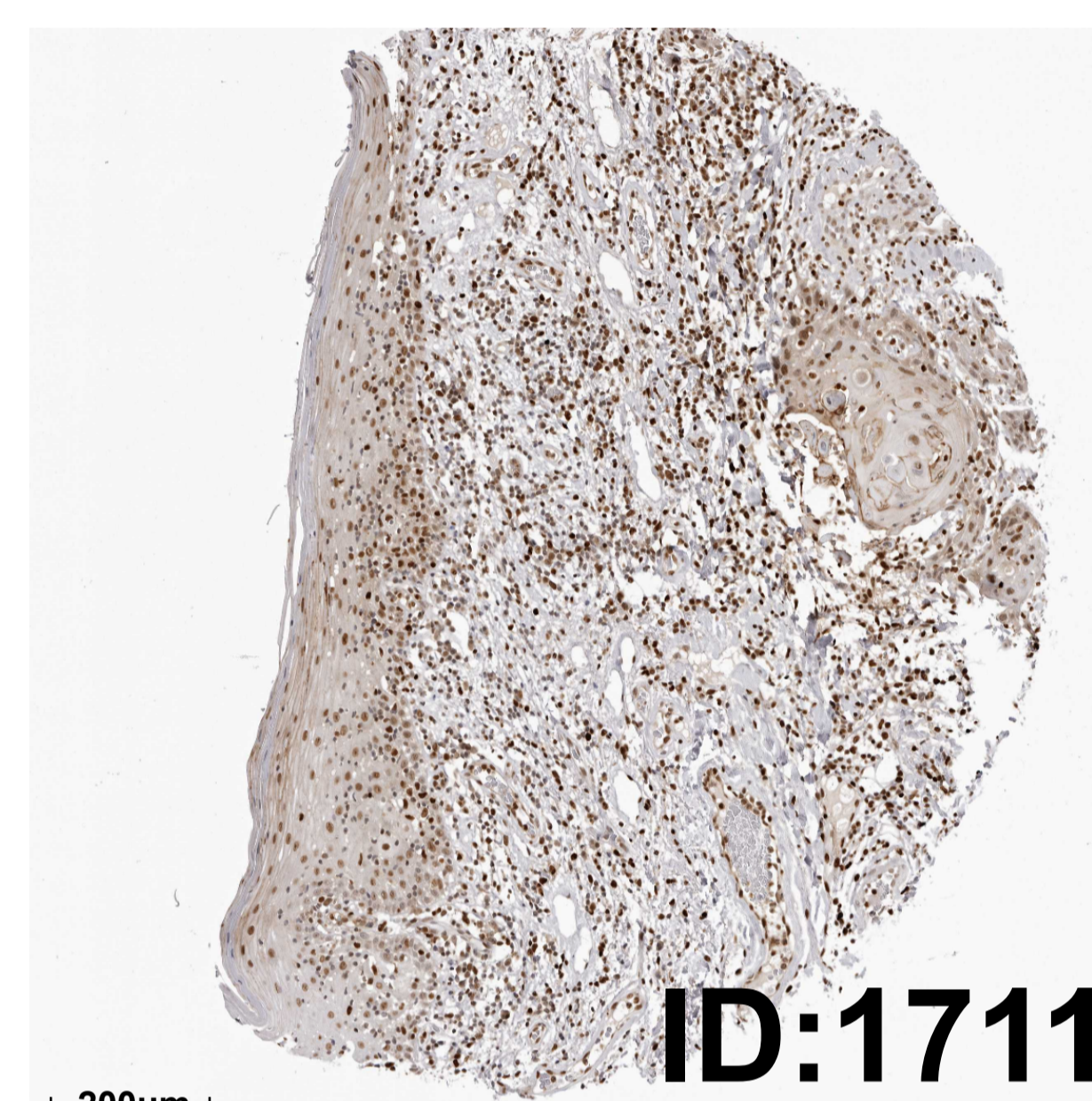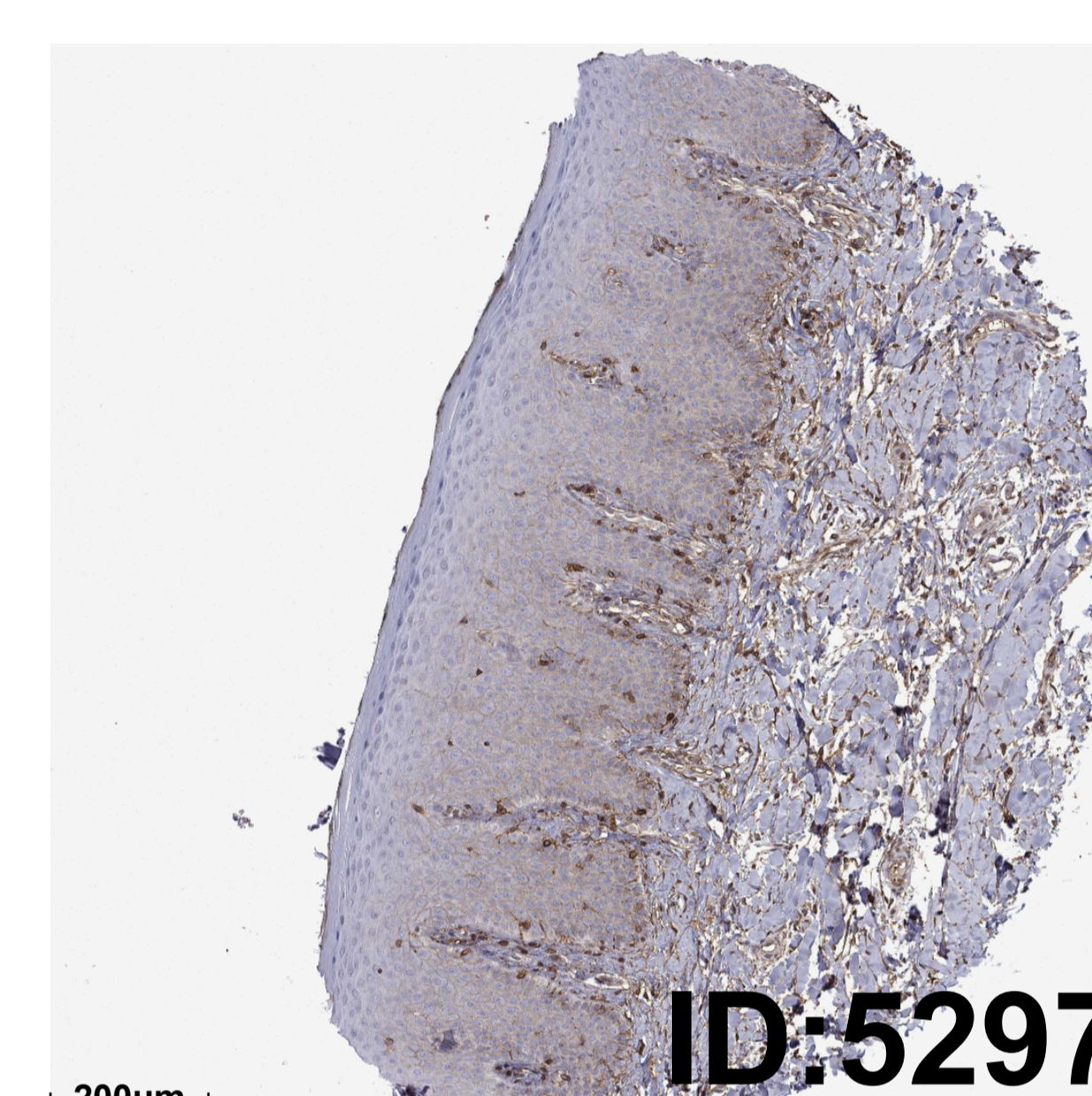

Cancer

P4HA1

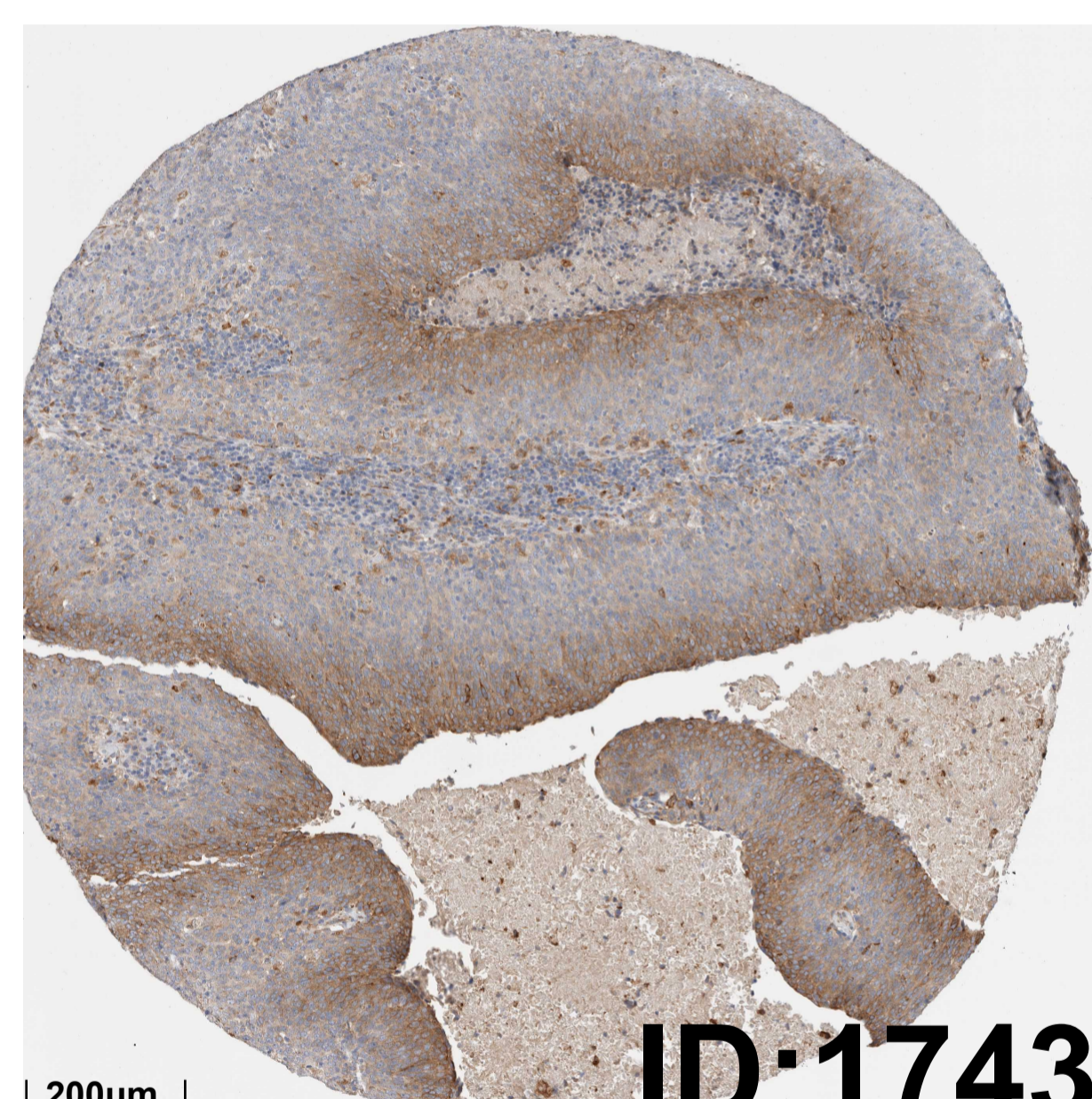

CTTN

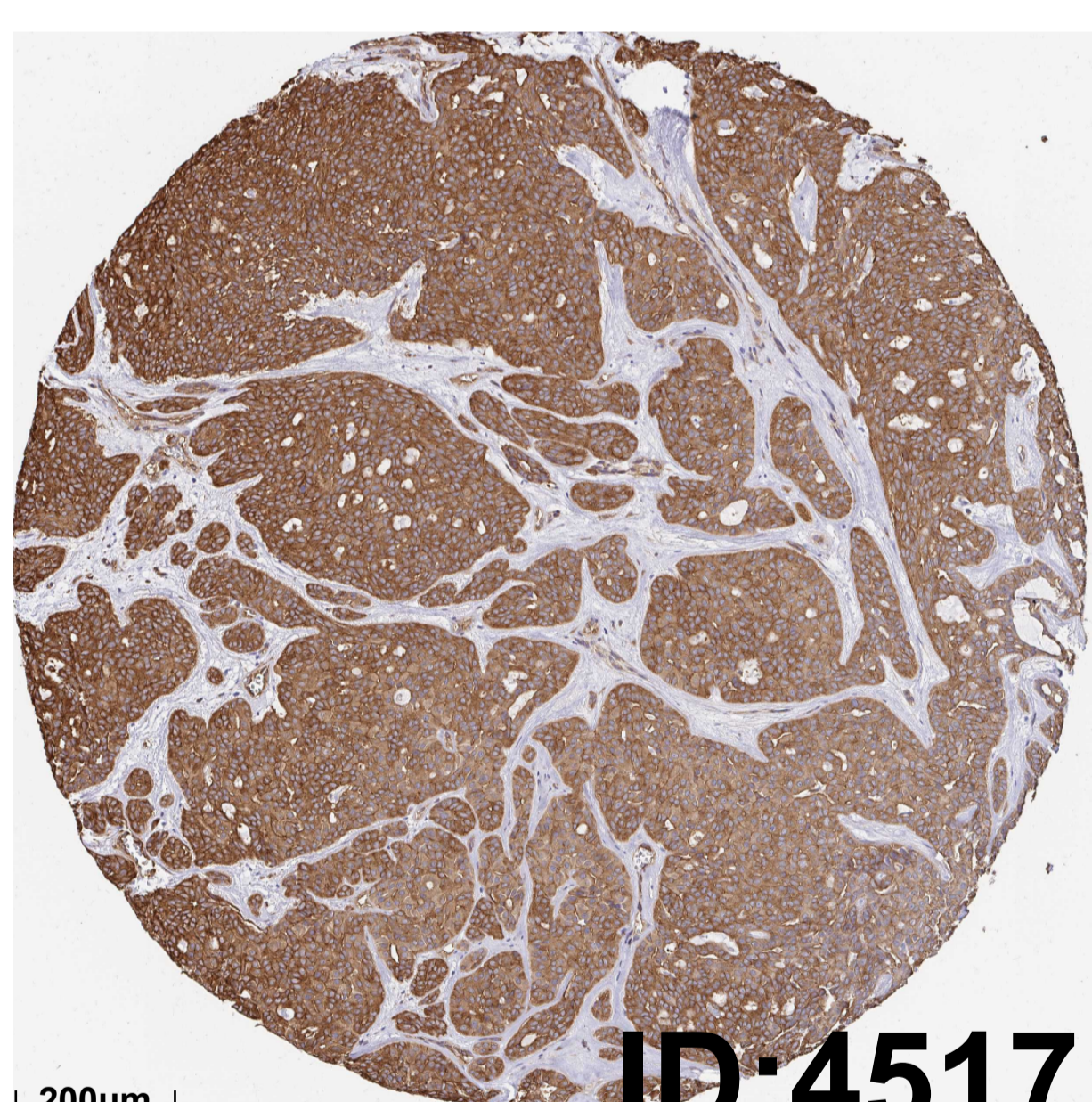

PRKCA

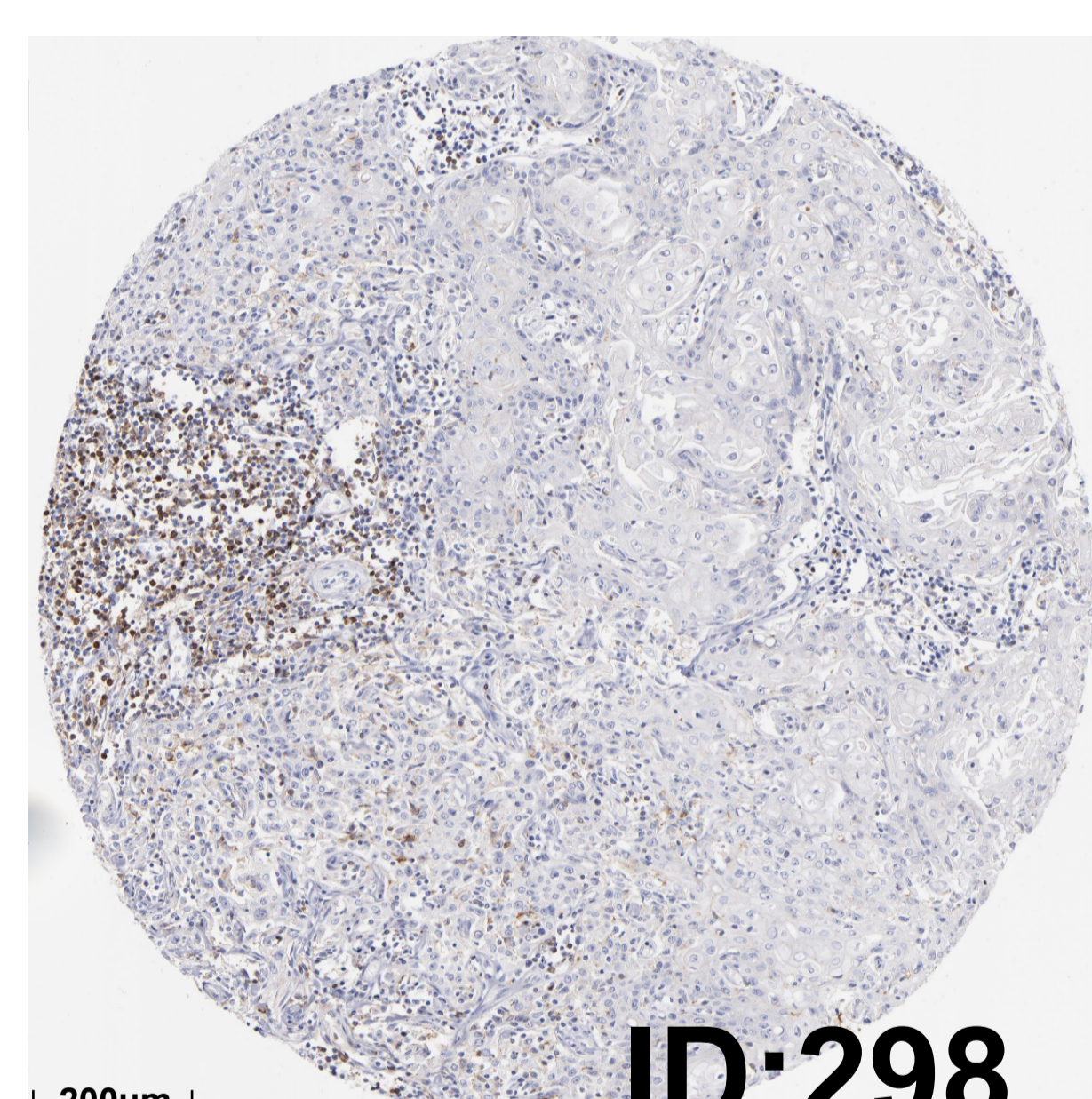

MCL1

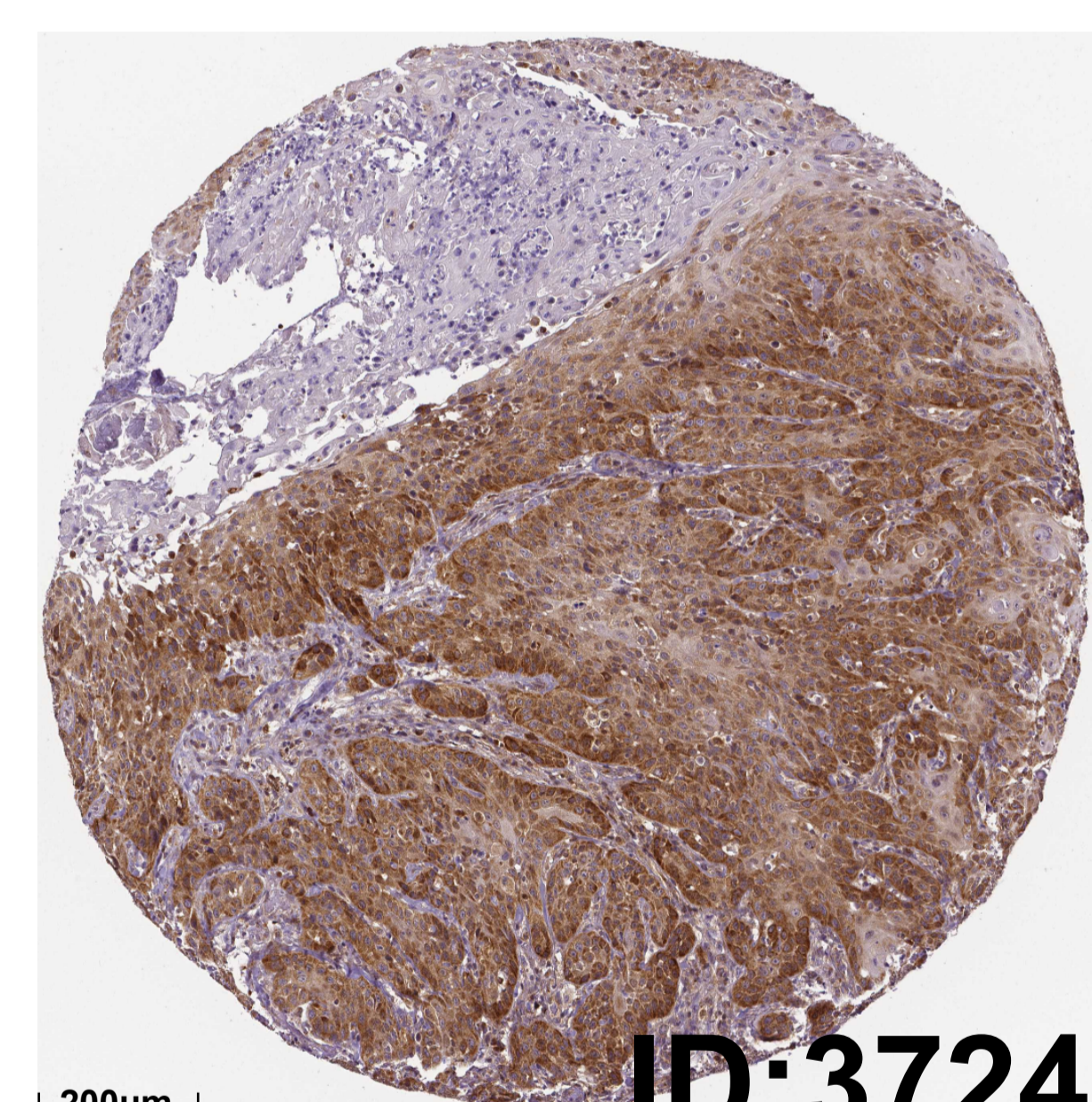

PAK4

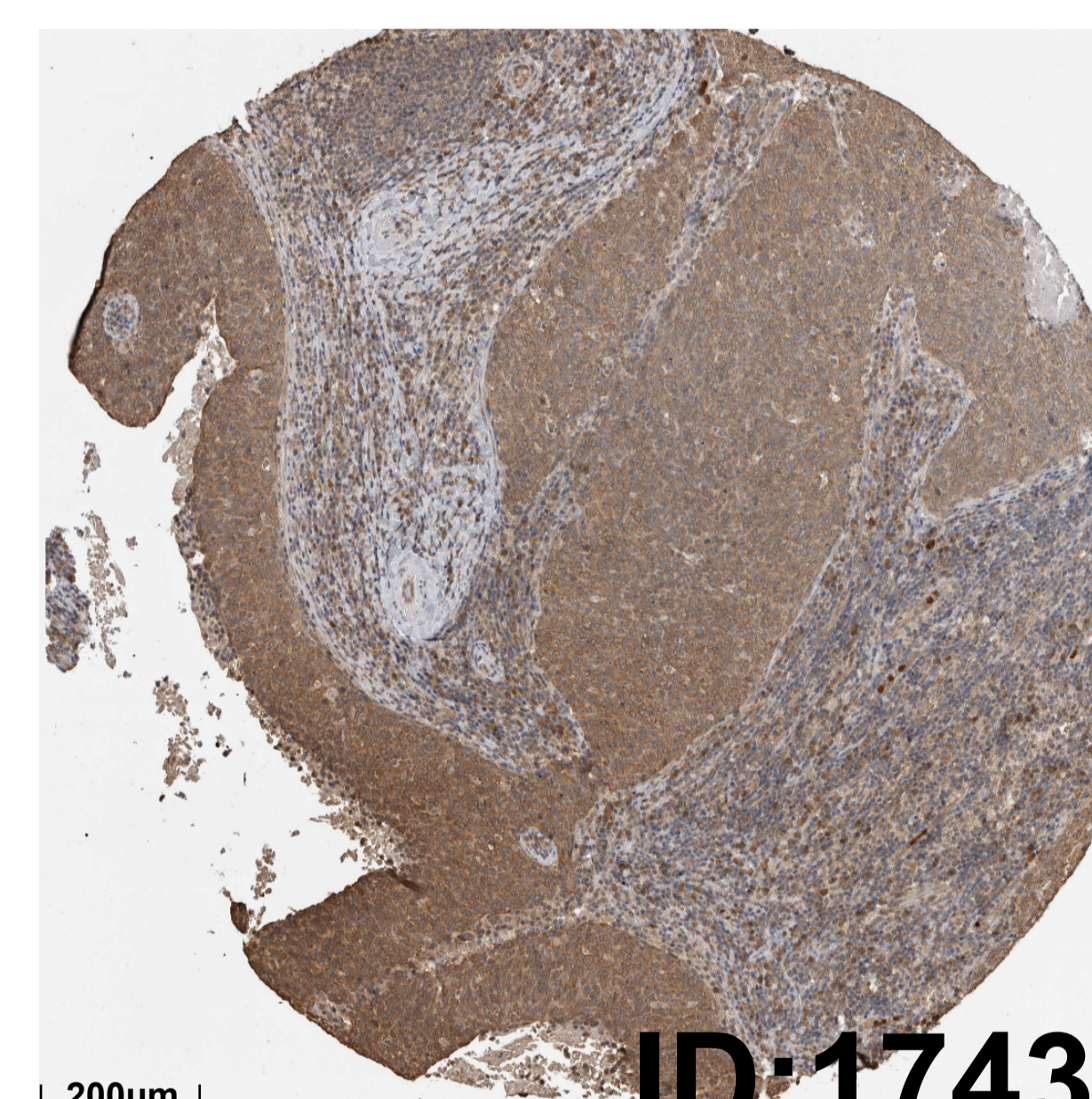

SPOCK2

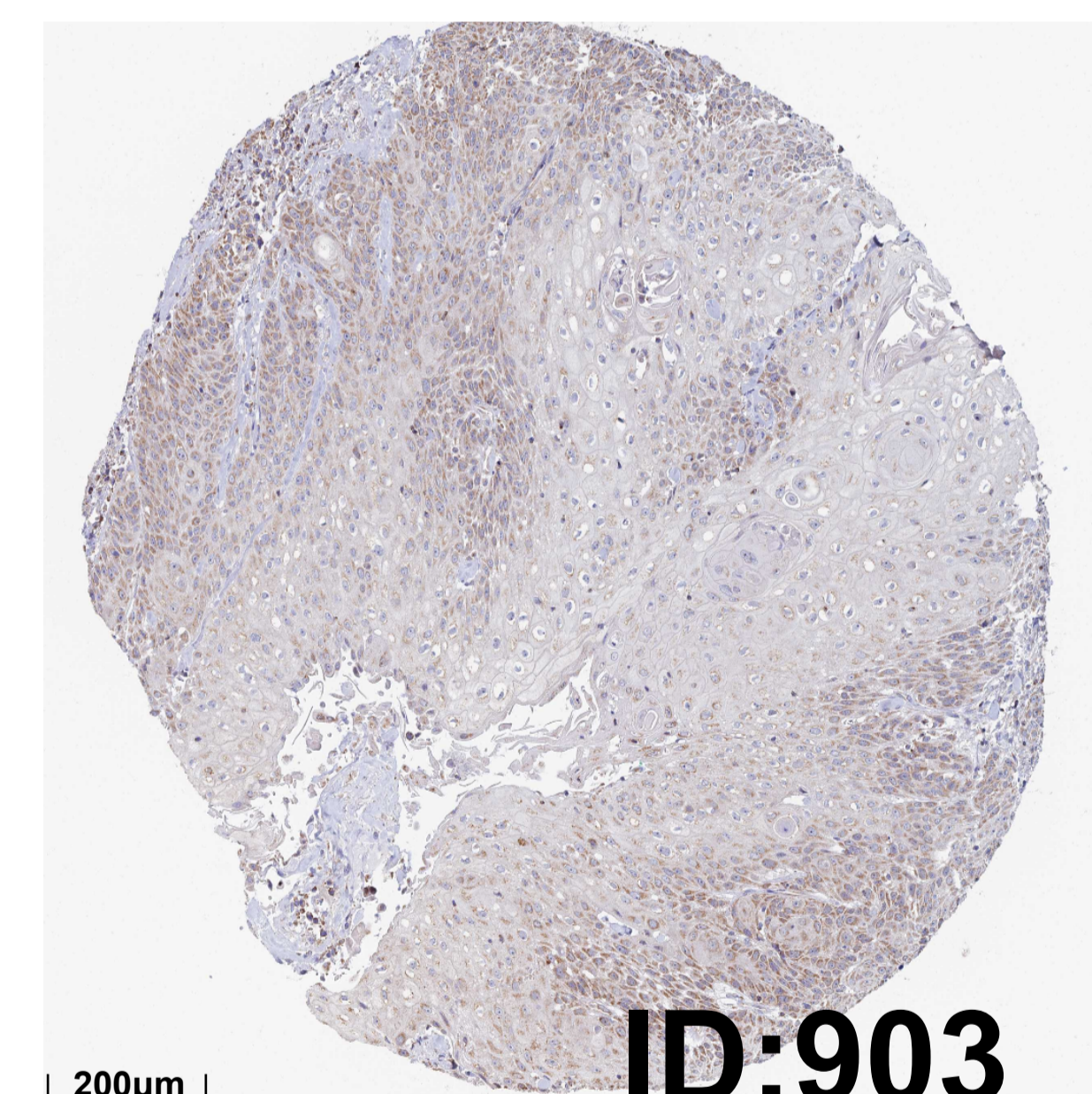

Normal

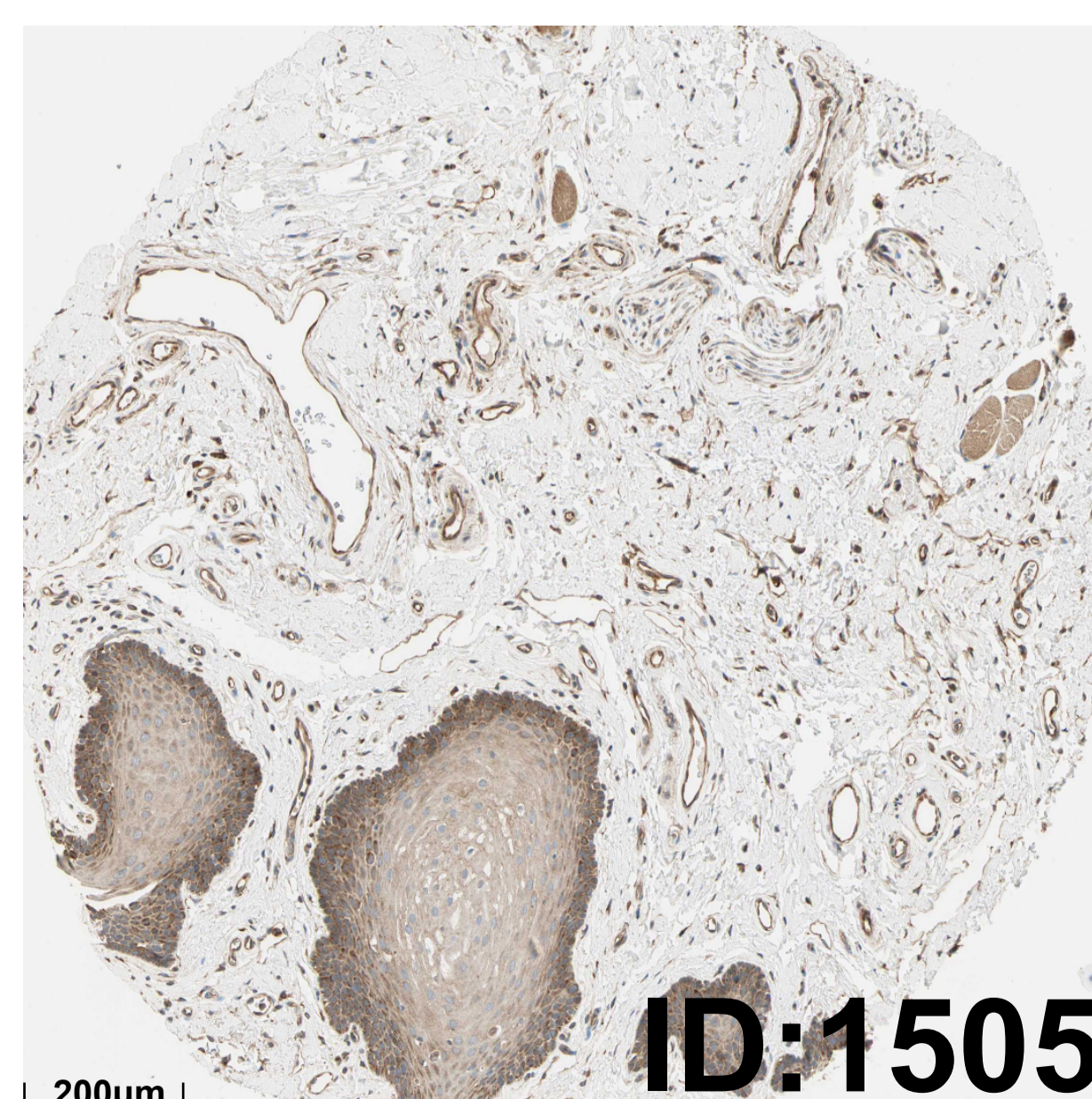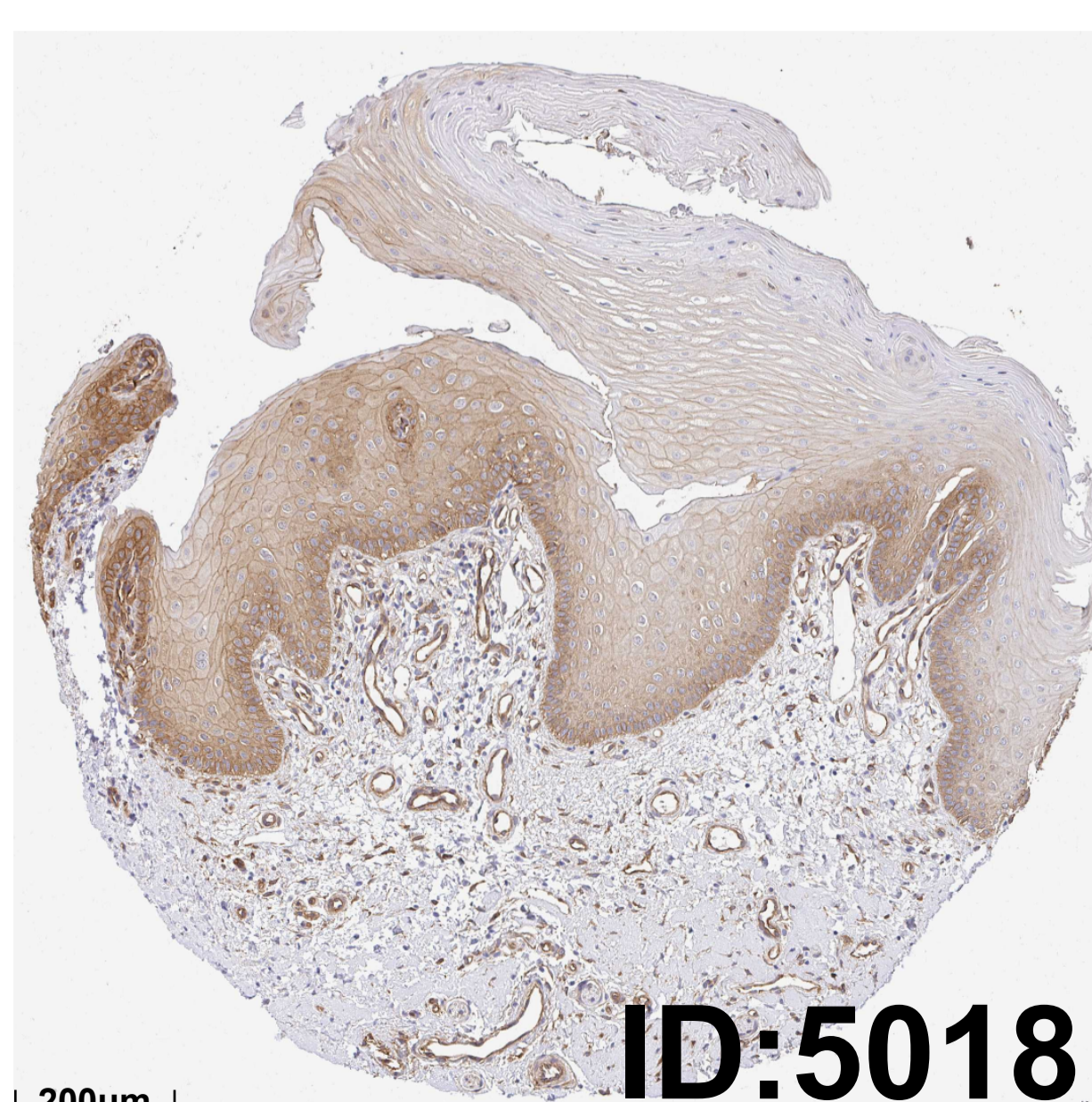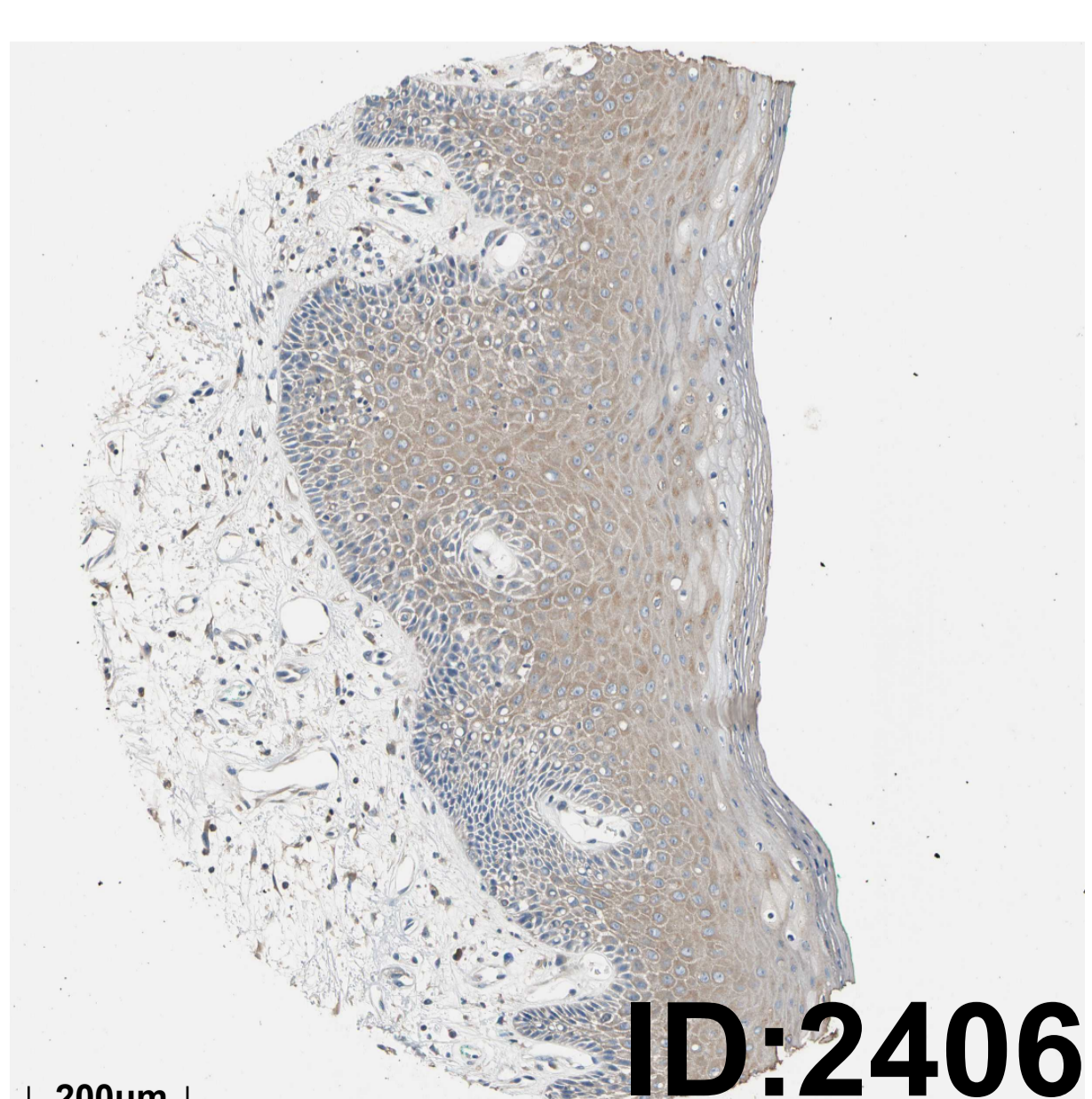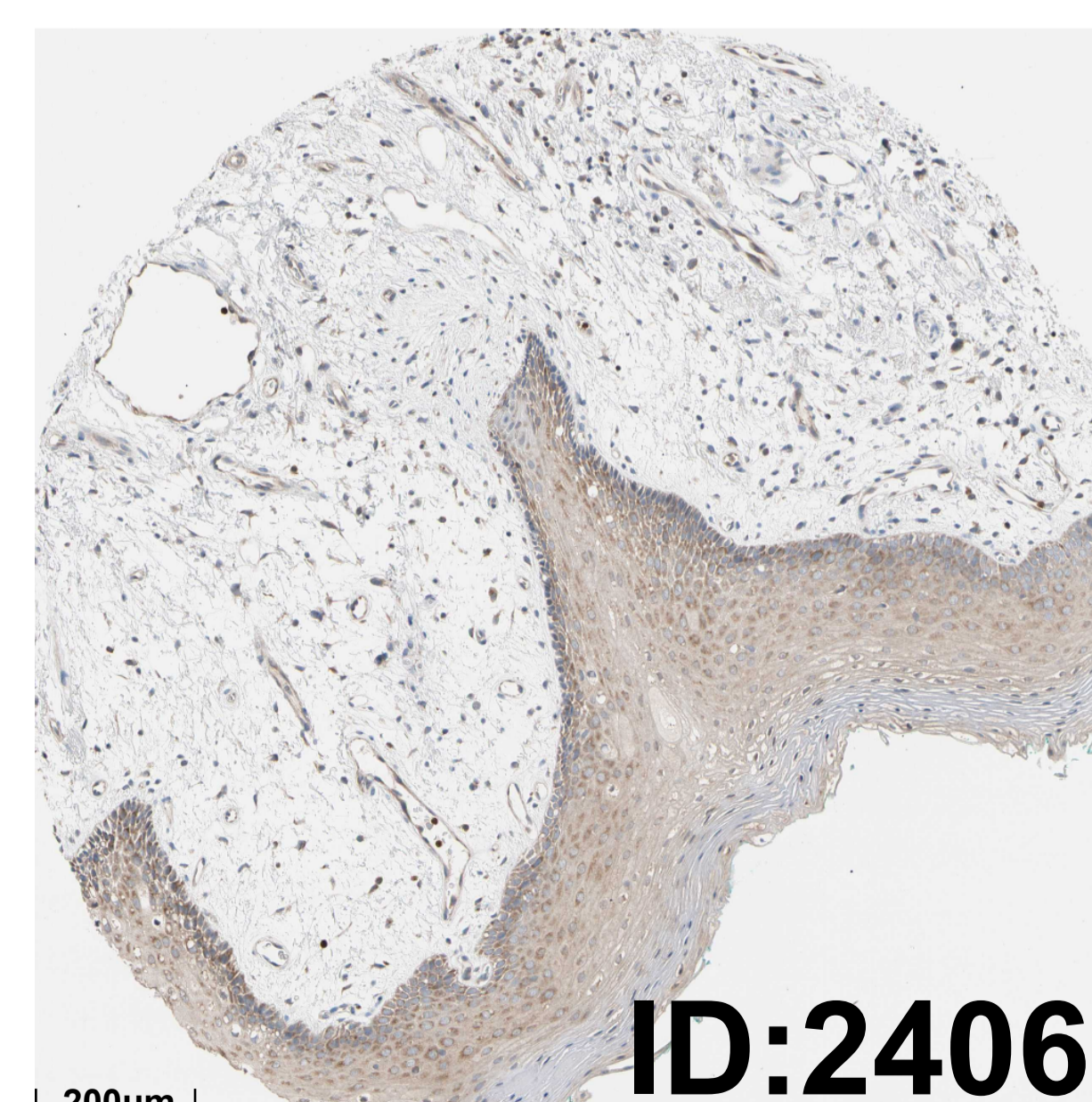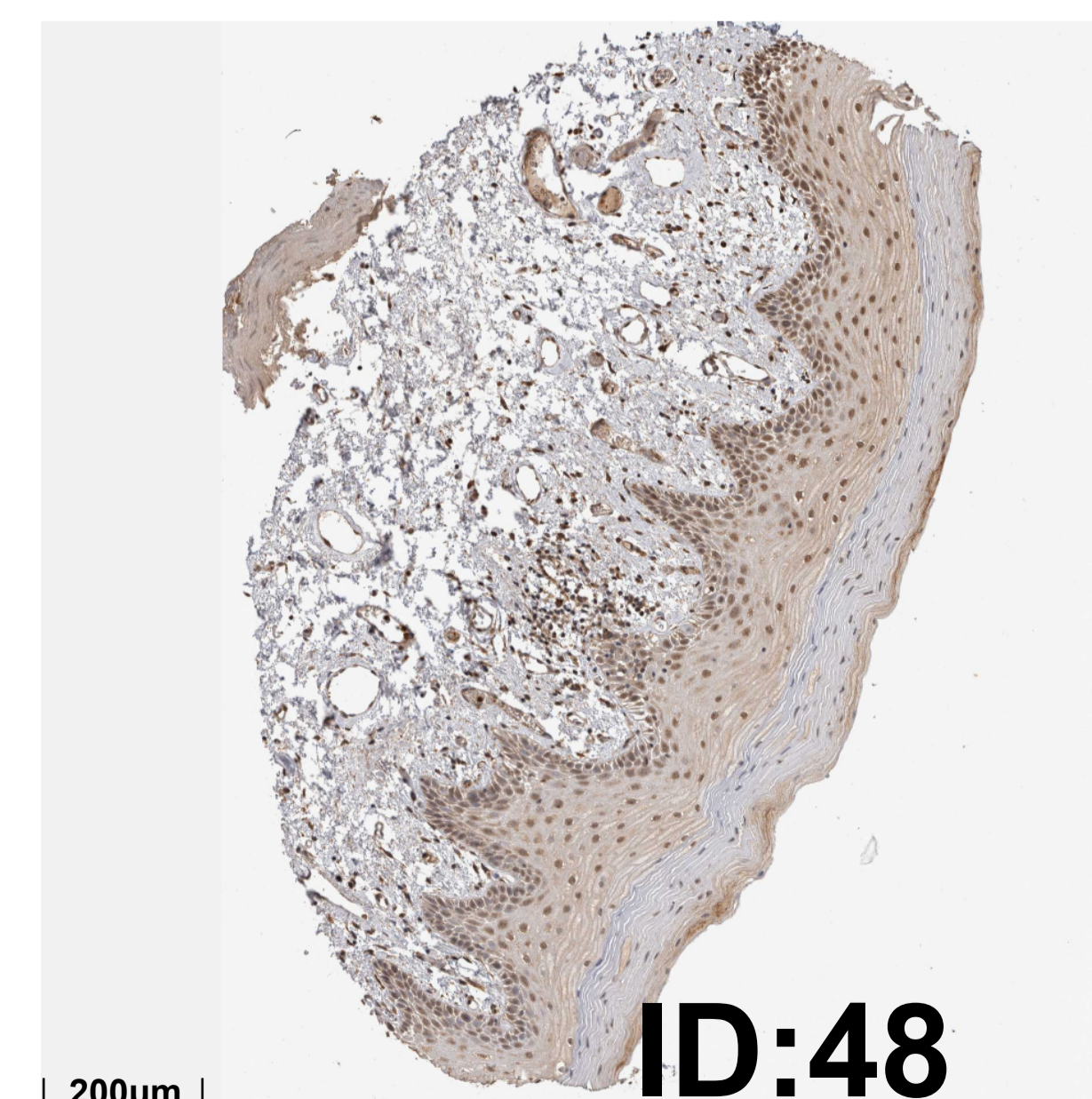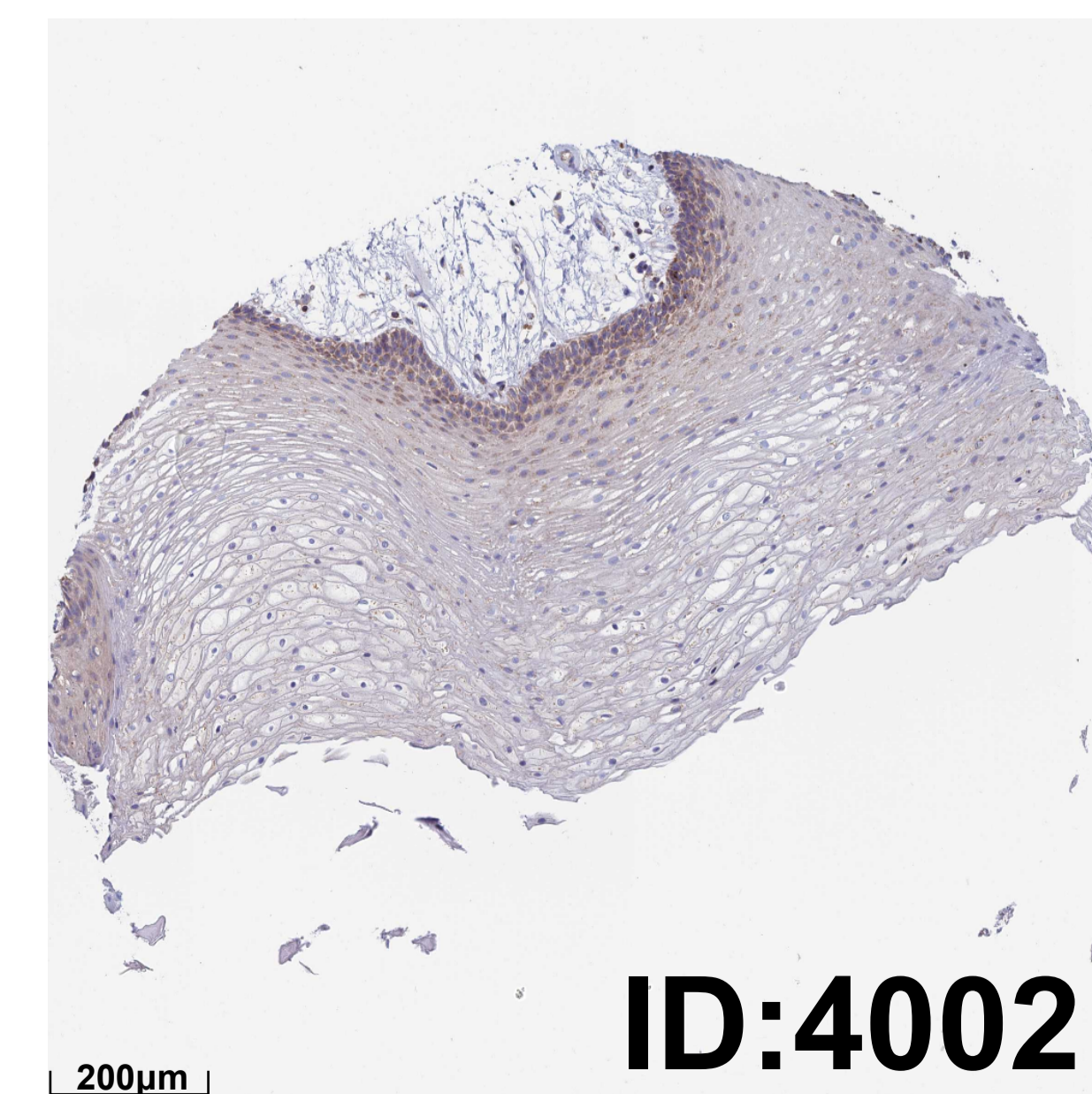

Supplement: Supplementary file 7 — Supplementary Material 7 [file 12967_2025_7336_MOESM7_ESM.pdf]

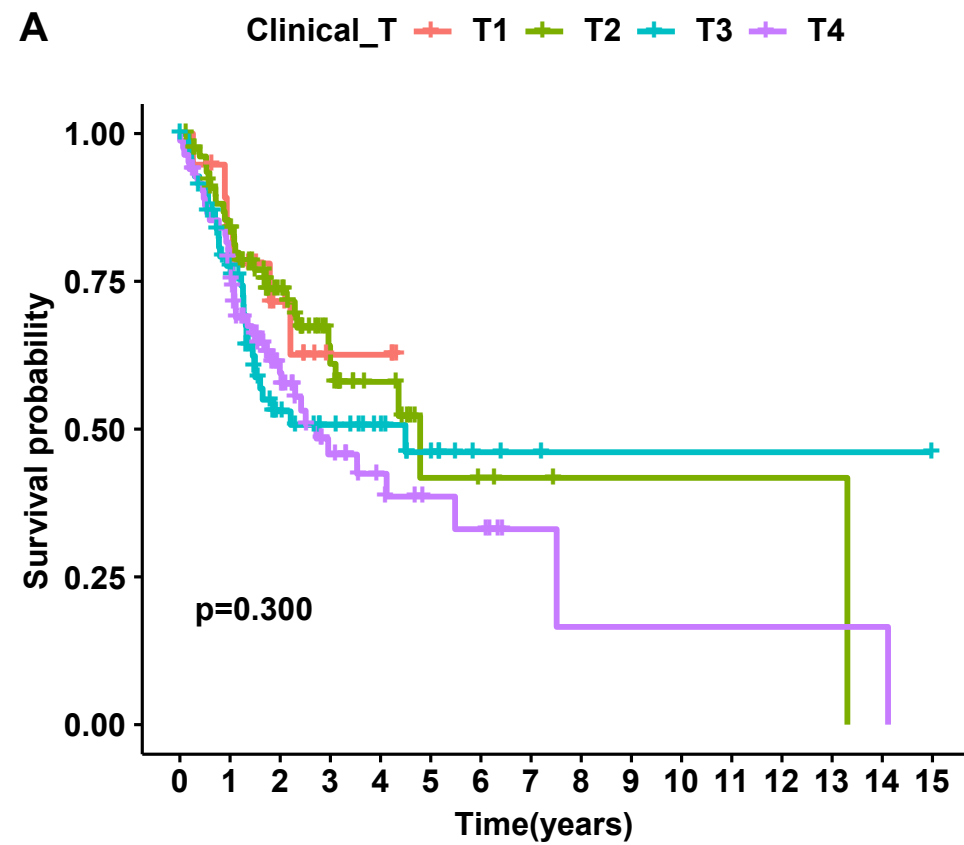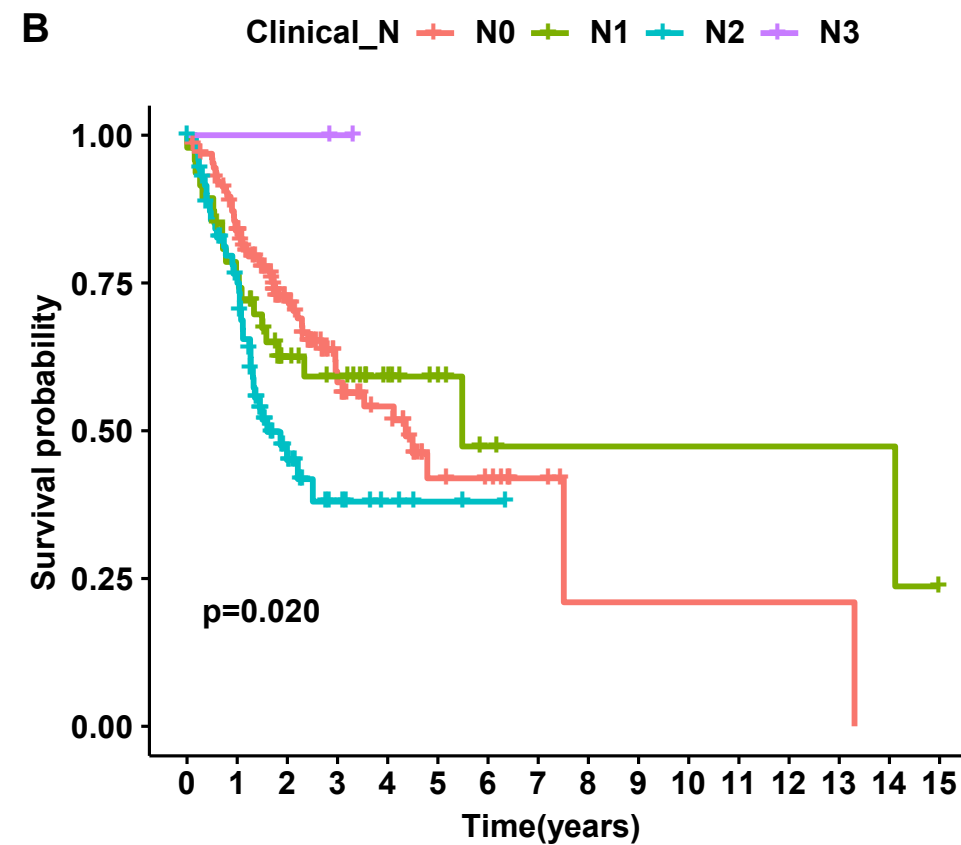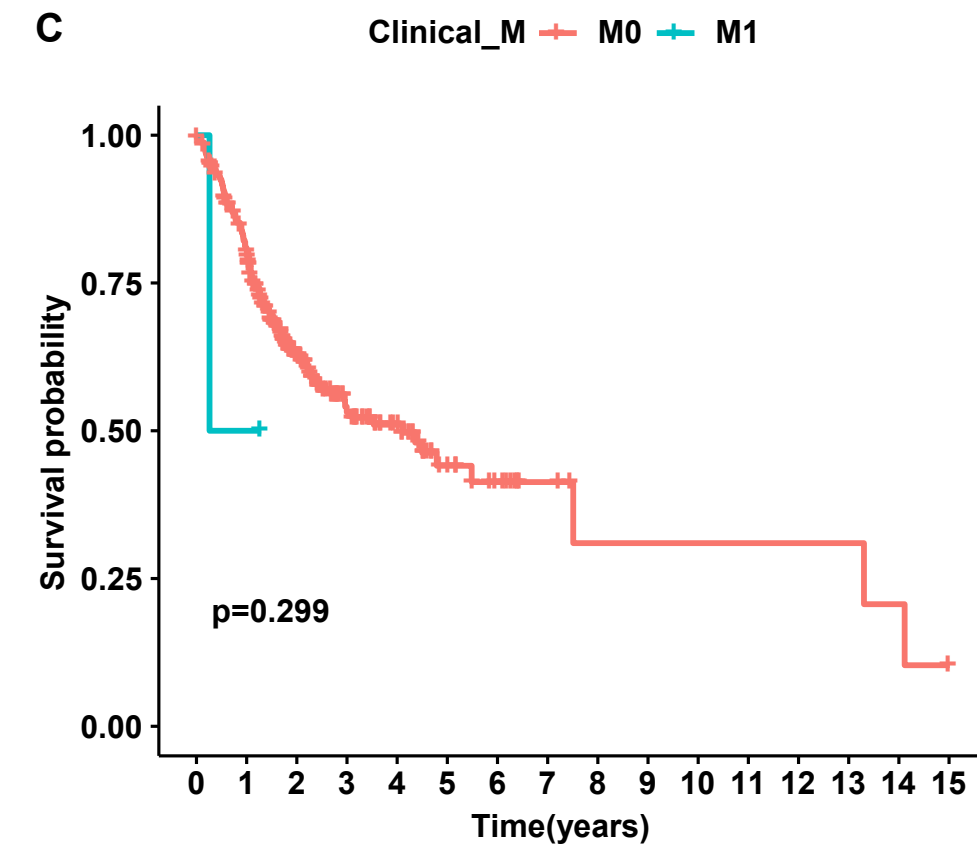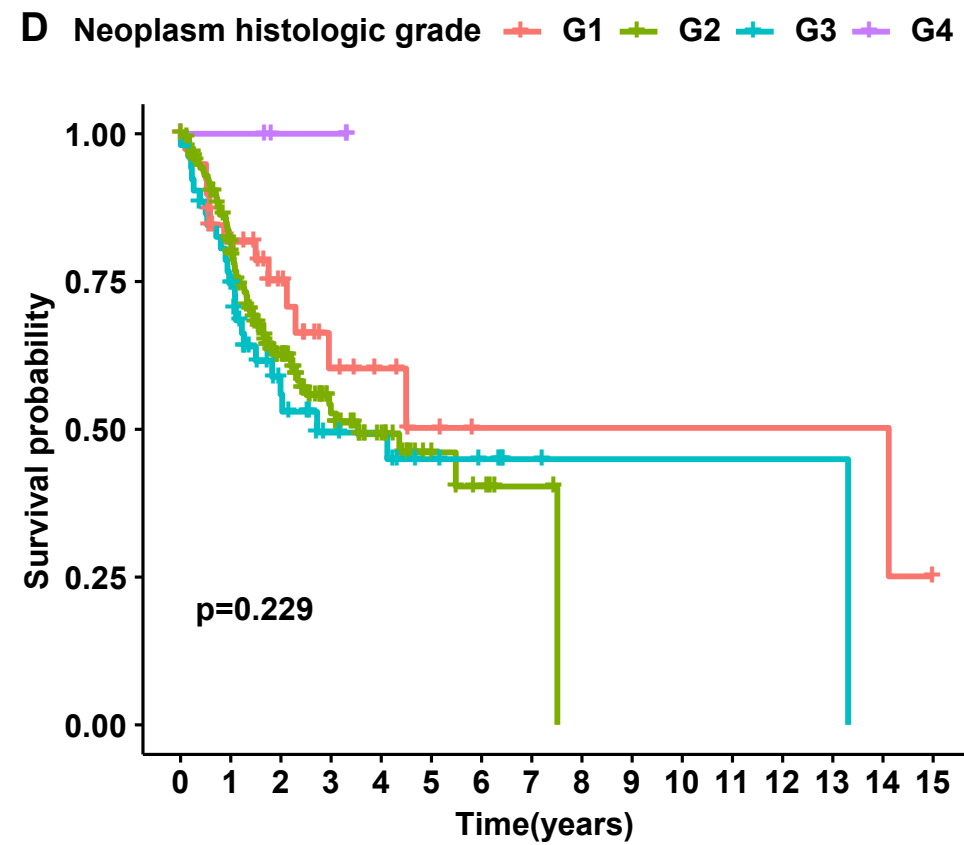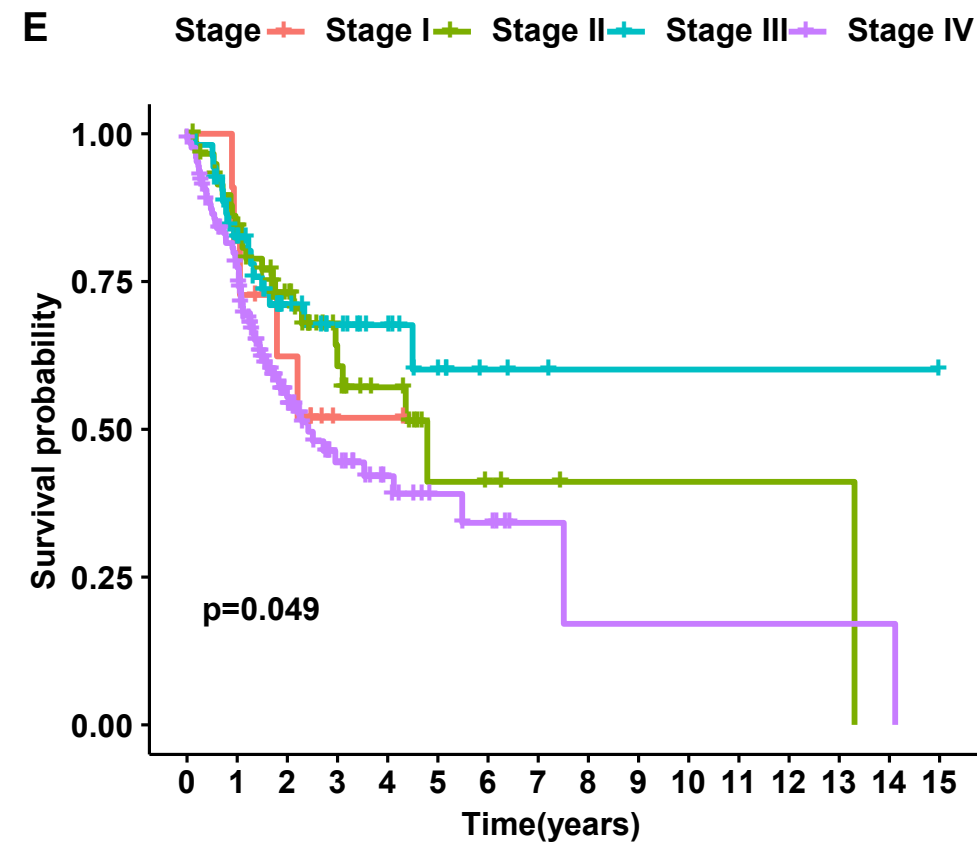

Supplement: Supplementary file 8 — Supplementary Material 8 [file 12967_2025_7336_MOESM8_ESM.pdf]

A

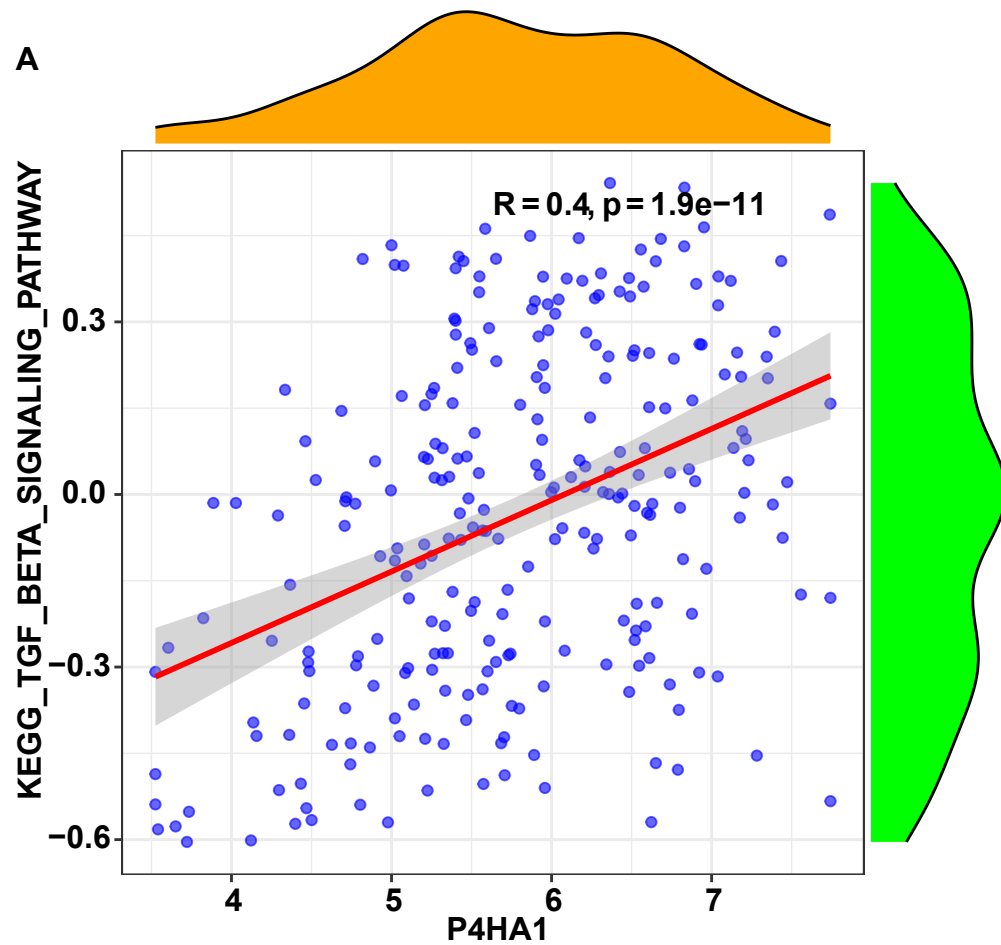

B

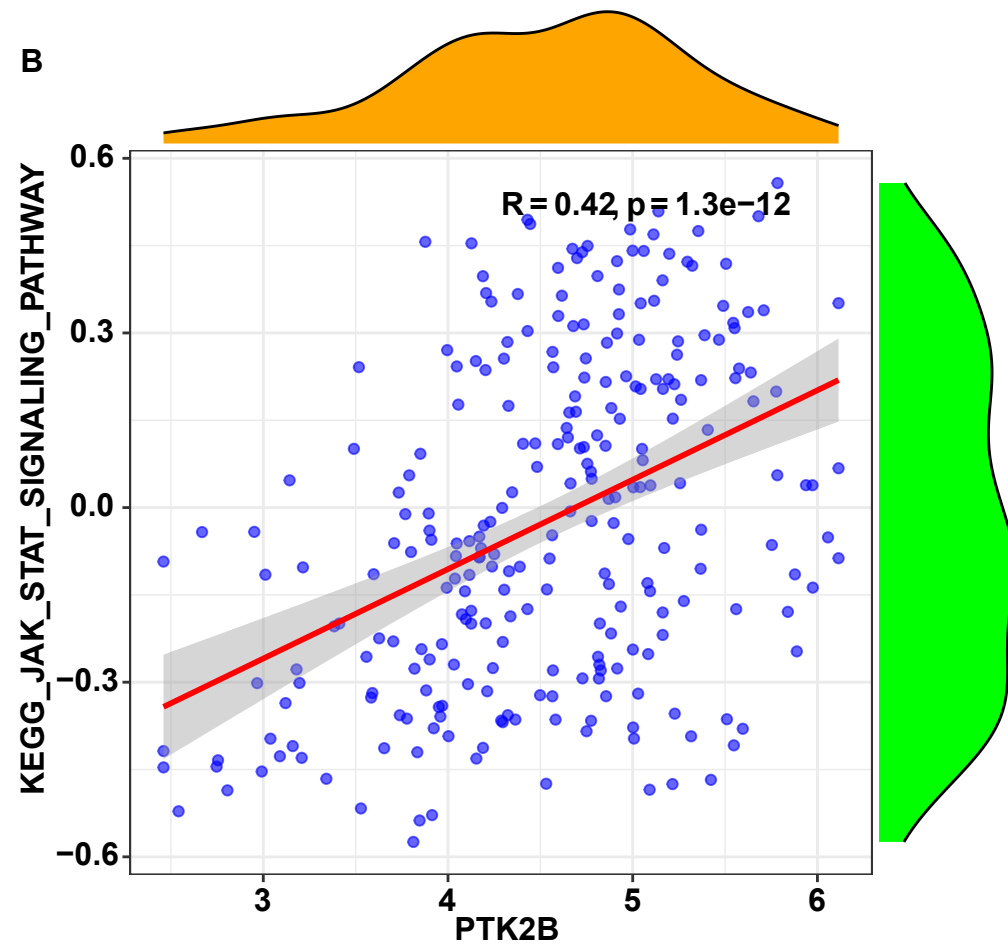

Supplement: Supplementary file 9 — Supplementary Material 9 [file 12967_2025_7336_MOESM9_ESM.pdf]

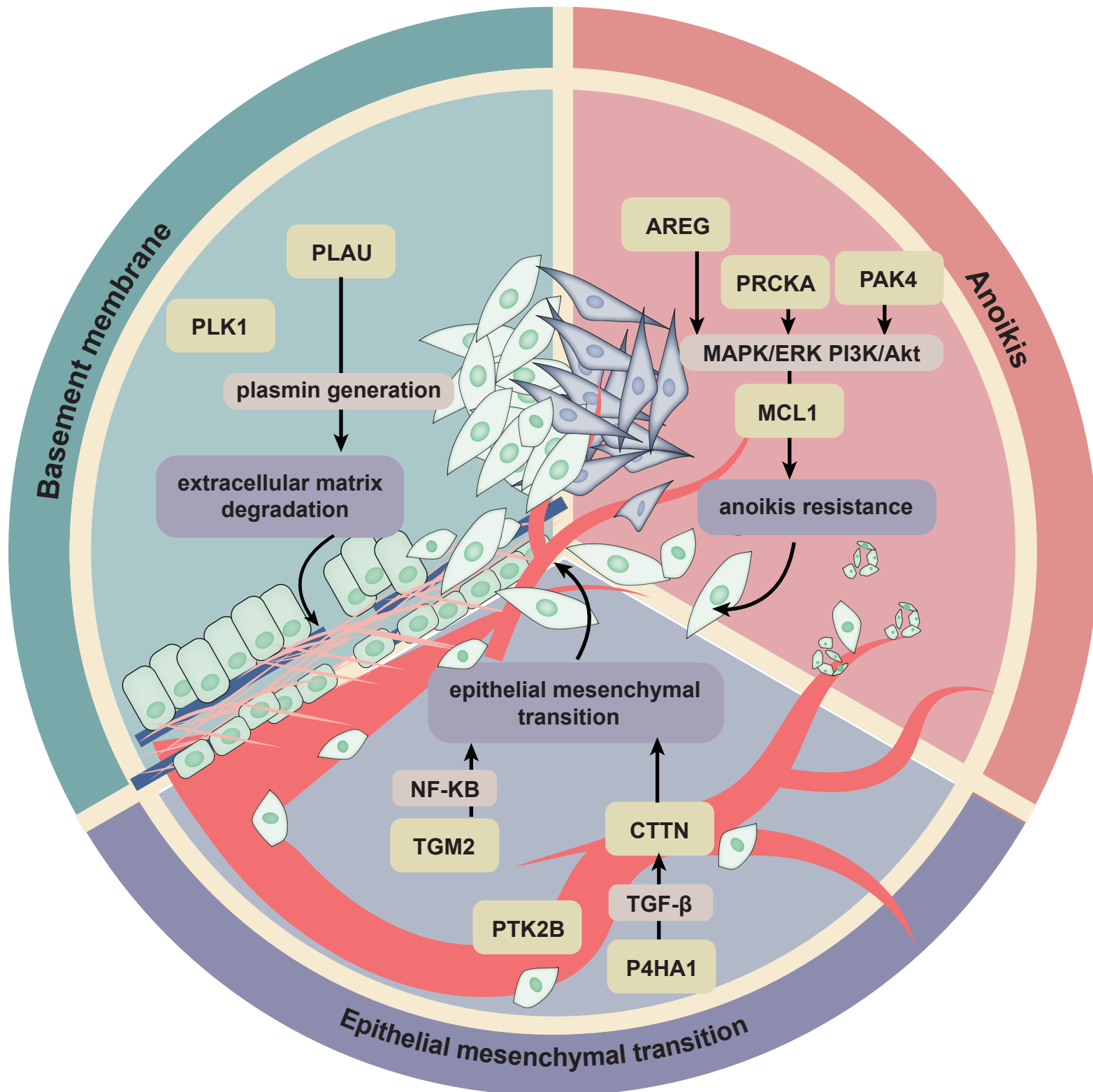

Gene product
  Biological Process
  Pathways or mechanism
 
●
 Primary
 
●
 Metastasis

Supplement: Supplementary file 10 — Supplementary Material 10 [file 12967_2025_7336_MOESM10_ESM.pdf]
