# Supplementary material for: Development of a cancer metastasis-associated risk model via multi-machine-learning algorithms for prognostic risk evaluation and clinical application in oral squamous cell carcinoma
Source: J Transl Med. 2025 Nov 24;23:1344. doi: 10.1186/s12967-025-07336-y (PMC12645686; doi:10.1186/s12967-025-07336-y)
Supplement: Supplementary file 15 — Supplementary Material 15 [file 12967_2025_7336_MOESM15_ESM.docx]

# **Supplementary figures**

**Figure S1. ssGSEA analysis revealed the enrichment of tumor metastasis-related genes in normal and OSCC tissues.** (A) The enrichment score of the tumor metastasis-related genes was significantly increased (Wilcoxon test) in OSCC compared to normal samples. (B) ROC analysis indicated that high enrichment of tumor metastasis-related genes contributes to the diagnosis of OSCC.

**Figure S2. Detailed model selection process.** Two machine learning algorithms, GBM and STEPCOX [forward], respectively exhibited the highest C-index value (0.7335) (Figure 2B), but both models included all 24 genes, which did not demonstrate proper screening capability and may be affected by overfitting. Thus, these models were not selected. Another machine learning algorithm, Enet, was used to optimize the L1 regularization ratio by adjusting the penalty parameter alpha. When alpha = 0, it corresponds to Ridge Regression, and when alpha = 1, it corresponds to Lasso Regression. Comparing Enet [alpha = 0.9] with Lasso (alpha = 1), although both models included the same 13 genes, the best predictive performance was achieved at alpha = 0.9. Therefore, alpha = 0.9 was considered the key parameter in model tuning, with Enet [alpha = 0.9] outperforming Lasso. When STEPCOX [forward], GBM and COXBOOST were applied individually, each model included more than 13 genes, and the average C-index was below 0.7015. Combining these algorithms with Enet [alpha = 0.9] enhanced the predictive ability, yielding results equivalent to those of Enet [alpha = 0.9] alone. Similarly, COXBOOST + Enet [alpha = 0.8] or COXBOOST + Enet [alpha = 0.9] yielded identical results. This is because COXBOOST have already performed gene selection in advance, making alpha = 0.8 sufficient for optimal predictive performance. However, both of these combinations were less straightforward and accurate compared to directly using Enet [alpha = 0.9]. Therefore, the model derived from Enet [alpha = 0.9] algorithm was deemed the optimal model.

**Figure S3. The stemness index analysis (Wilcoxon test) showed the distribution of stemness indices assessed by mRNAsi in High-TMI and Low-TMI groups.**

**Figure S4. Analysis of the GSE275870 dataset revealed higher TMI level in metastatic lymph node tissues compared to primary tumors.**

**Figure S5. GO enrichment analysis of TCGA cohort.**

**Figure S6. Immune infiltration analysis of the validation cohort GSE41613.** (A) Boxplot showing differences in the infiltration levels of 16 immune cell types between High-TMI and Low-TMI groups based on ssGSEA analysis. (B) Correlation analysis of signature genes, TMI, 16 immune cell types and 13 immune functions. (C) The type II IFN response was inversely correlated with TMI. (D) The infiltration level of mast cells was inversely correlated with TMI. (E) TIDE analysis results of TCGA cohort. (F) TIDE analysis results of GSE41613 cohort. Wilcoxon test (A) and Pearson correlation (B-D) were used; p values are shown.

**Figure S7. HPA-based verification of differential expression patterns of signature gene between OSCC and normal tissues.**

**Figure S8. Kaplan Meier survival curves stratified by clinicopathological features.**

(A-E) Survival analysis based on Clinical T stage, Clinical_ N, Clinical_ M, neoplasm histologic grade and Stage.

**Figure S9. Correlation analysis between gene-encoded proteins and signaling pathways.**

(A) Correlation of P4HA1 with the TGF-β signaling pathway. (B) Correlation of PTK2B with the JAK-STAT signaling pathway.

**Figure S10. Mechanism diagram of the signature genes promoting metastasis hypothesis.**
